# Temporal dynamics of miRNAs in human DLPFC and its association with miRNA dysregulation in schizophrenia

**DOI:** 10.1038/s41398-019-0538-y

**Published:** 2019-08-20

**Authors:** Zhonghua Hu, Shouguo Gao, Daniel Lindberg, Debabrata Panja, Yoshi Wakabayashi, Keshen Li, Joel E. Kleinman, Jun Zhu, Zheng Li

**Affiliations:** 1 Section on Synapse Development and Plasticity, National Institute of Mental Health, National Institutes of Health, Bethesda, MD 20892 USA; 2 Systems Biology Center, National Heart Lung and Blood Institute, National Institutes of Health, Bethesda, MD 20892 USA; 3 Institute of Neurology, Affiliated Hospital of Guangdong Medical University, Zhanjiang, Guangdong, China; 4 Clinical Neuroscience Institute of Jinan University, Guangzhou, China; 5 Lieber Institute for Brain Development, Baltimore, MD 21205 USA

**Keywords:** Neuroscience, Psychiatric disorders

## Abstract

Brain development is dependent on programmed gene expression, which is both genetically and epigenetically regulated. Post-transcriptional regulation of gene expression by microRNAs (miRNAs) is essential for brain development. As abnormal brain development is hypothesized to be associated with schizophrenia, miRNAs are an intriguing target for this disorder. The aims of this study were to determine the temporal dynamics of miRNA expression in the human dorsolateral prefrontal cortex (DLPFC), and the relationship between miRNA’s temporal expression pattern and dysregulation in schizophrenia. This study used next-generation sequencing to characterize the temporal dynamics of miRNA expression in the DLPFC of 109 normal subjects (second trimester–74 years of age) and miRNA expression changes in 34 schizophrenia patients. Unlike mRNAs, the majority of which exhibits a wave of change in fetuses, most miRNAs are preferentially expressed during a certain period before puberty. It is noted that in schizophrenia patients, miRNAs normally enriched in infants tend to be upregulated, while those normally enriched in prepuberty tend to be downregulated, and the targets of these miRNAs are enriched for genes encoding synaptic proteins and those associated with schizophrenia. In addition, miR-936 and miR-3162 were found to be increased in the DLPFC of patients with schizophrenia. These findings reveal the temporal dynamics of miRNAs in the human DLPFC, implicate the importance of miRNAs in DLPFC development, and suggest a possible link between schizophrenia and dysregulation of miRNAs enriched in infancy and prepuberty.

## Introduction

Brain development is profoundly influenced by gene expression, which is dynamically regulated across the lifespan^1–3^. The temporal dynamics of gene expression are conferred by transcriptional and post-transcriptional regulation. Among the post-translational regulators, microRNAs (miRNAs) have emerged as important players in brain development and the pathophysiology of psychiatric disorders. miRNAs are small (~16–25 nt) non-coding RNAs that bind to the 3′ UTR of target mRNAs through imperfect complementarity to destabilize mRNAs or inhibit translation^4–6^. miRNA expression in the brain is developmentally regulated and correlates with cortical maturation^7–10^. A microarray and TaqMan-based expression study shows that 312 miRNAs in the DLPFC change between at least two of the broad age groups: fetal, early postnatal (1–4 years of age), and adult (based on one 81-year-old adult brain and an RNA sample pooled from adults with an average age of 68 years)^9^. A later miRNA microarray study using only postnatal brains shows that global miRNA expression in the DLPFC is relatively high during the early years and declines after adolescence^8^. However, these previous studies are limited by incomplete coverage of the human lifespan. A comprehensive temporal dynamics of miRNA expression throughout the fetal to senior life has yet to be delineated.

miRNAs function in the differentiation, development, and plasticity of neurons^11–16^. Global miRNA loss in mice caused by the deletion of genes involved in miRNA biogenesis influences synaptic protein expression, synaptic transmission, dendritic spines, learning, and memory^5,17,18^. Despite the mounting evidence supporting the importance of miRNAs in neurons, only a few miRNAs have been studied in neural development and synaptic function. These miRNAs include miR-134 which restricts the size of dendritic spines through translational inhibition of Limk1^19^ and mediates depolarization- and BDNF- induced dendritic growth by downregulating Pum2 (an RNA-binding protein that controls dendritic morphology)^20^; miR-132 which promotes dendritic growth through repression of p250GAP (a Rho family GTPase-activating protein)^21^; and miR-191, −384-5p, −135, −137, −26a, −124, −92, −132, and −212 which regulate synaptic plasticity^22^. In addition, genes encoding miRNAs or components in the miRNA biogenesis machinery have been implicated in psychiatric disorders such as schizophrenia^23–27^. Analysis of CNVs (copy number variants) shows that 22q11.2 deletion confers an increased risk for schizophrenia^28,29^. The 22q11.2 locus contains seven miRNA genes and *DGCR8*, a double-stranded-RNA-binding protein involved in miRNA biogenesis^30–32^. Moreover, a genome-wide survey of miRNAs in rare CNVs shows that the schizophrenia group is enriched with individuals having rare CNVs overlapping a miRNA gene^33^. GWAS (genome-wide association study) has also implicated miRNAs in schizophrenia. The 108 genomic loci associated with schizophrenia identified by Psychiatric Genomics Consortium contain 22 miRNA genes, and the *MIR137* gene, one of the 22 miRNA genes, is among the top genomic loci associated with schizophrenia in two other GWAS with >10,000 samples^34–38^. miR-9 is dysregulated in neural progenitor cells derived from schizophrenia patients, and the target gene sets of miR-137 and miR-9-5p are top miRNA targetomes enriched with schizophrenia risk genes^34,39,40^.

In addition to genetic associations between miRNA genes and schizophrenia, many groups have detected miRNA expression changes in the postmortem brains of people with schizophrenia using microarray and quantitative PCR^23^. The reported findings, however, are inconsistent^41–44^. The inconsistency is attributable, at least in part, to biological and technical variations including sample demographics, array platforms, and data analysis methods, as well as antemortem treatments and substance abuse which are common confounds of postmortem human brain studies.

Given the implication of miRNAs in brain development and schizophrenia, in this study, we delineate the temporal dynamics of miRNAs expressed in the human DLPFC across the majority of lifespan and identified miRNAs that are altered in the brains of schizophrenia patients. Our study shows that in normal brains, most miRNAs are expressed preferentially during certain periods before puberty. In the DLPFC of schizophrenia patients, miRNAs normally enriched in infants increase, while those in prepuberty decrease, and the targets of these miRNAs are enriched for synaptic proteins and genes associated with schizophrenia. These findings reveal a tight temporal control of miRNA expression in the human DLPFC before puberty. They also suggest a connection between dysregulated miRNA expression and synaptic dysfunction in schizophrenia.

## Materials and methods

### Postmortem human brains

All postmortem human brains were obtained from the Offices of the Chief Medical Examiner of the District of Columbia, and of the Commonwealth of Virginia, Northern District, all with informed consent from the legal next of kin (protocol 90-M-0142 approved by the NIMH/NIH Institutional Review Board). Diagnoses, macro- and microscopic neuropathological examinations and toxicological analysis were performed on all cases. The controls for the analysis of miRNA differential expression in schizophrenia cases are the normal subjects ≥18-year-old used in the miRNA temporal profiling study. The demographics of brain donors are summarized below.

**Table:** 

Analysis	Controls			Schizophrenia cases		
	Mean/range	Ethnicity	Gender	Mean/range	Ethnicity	Gender
	(Age in year)			(Age in year)		
miRNA/mRNA temporal dynamics	29.9/−0.44–73.3	62 AA	62 M 46 F	N/A	N/A	N/A
		1 AS				
		45 CAUC				
Differential expression of miRNAs in schizophrenia patients and controls	43.3/18.2–73.3	64 AA	62 M	50.2/18.6–71.9	22 AA	20 M
		38 CAUC	40 F		12 CAUC	14 F

*AA* African American, *AS* Asian, *CAUC* Caucasian, *M* Male, *F* Female, *N/A* not applicable

Fetal brain specimens were provided by Drs. Ronald Zielke, Robert D. Vigorito, and Robert M. Johnson of the National Institute of Child Health and Human Development Brain and Tissue Bank for Developmental Disorders at the University of Maryland.

### Next-generation sequencing, real-time PCR, and data analysis

The preparation of miRNA libraries for next-generation sequencing (NGS) was performed as previously described^14^. In brief, miRNAs were isolated from tissues using *mir*Vana™ miRNA Isolation Kit followed by gel electrophoresis to collect 17–27 nucleotide fragments. Purified miRNAs were ligated to 3′ and 5′ adapters which contain unique index sequences and then reverse-transcribed and PCR amplified with Small RNA Sample Prep kit (Illumina). The PCR products were purified using polyacrylamide gel electrophoresis and sequenced with the Illumina HiSeq-2000 platform. NGS data were analyzed with our custom script. The raw sequence reads were consolidated by clustering identical sequence reads, trimmed to remove the adapter and then mapped to reference miRNA hairpin sequences in miRbase (http://www.mirbase.org) using the Bowtie alignment tool (bowtie-0.12.5)^45^. The mapped reads were assigned to specific miRNAs only if their sequences have an ≥80% overlap with a mature miRNA. The read count of individual miRNAs in each library was converted to count per million (CPM) to normalize for sequencing depth and CPM was used to represent miRNA expression level. CPM was log-transformed and imported into Partek Genomic Suite (version 6.6; Partek Inc., St. Louis, MO) for principal component analysis (PCA, a mathematical algorithm that reduces the dimensionality of the data so that each sample can be represented by a small number of variables). In the resulting PCA plots, each sample was colored based on its diagnosis, race, age, gender, pH, batch, or PMI to visually inspect for potential sample segregation. To identify miRNAs differentially expressed in schizophrenia cases, generalized linear model (GLM, a flexible generalization of ordinary linear regression that allows for variables that have distribution patterns other than a normal distribution) in the software package edgeR (Empirical Analysis of DGE in R)^46^ was employed to calculate *p*-values for schizophrenia vs. control samples by taking into account the potential contribution of race, age, and gender. *P*-values were adjusted with the Benjamini–Hochberg false discovery rate (FDR) procedure.

The effect of psychiatric medications was analyzed in schizophrenia cases with GLM using lifetime, daily and last chlorpromazine (CPZE) dose, antidepressant, and smoking (determined by toxicology) as variables. The effect of smoking in control samples was analyzed with GLM using age (young adult: 18–39 years; adult: 40–60 years; senior: >60 years), sex and race as variables in adult samples, and cotinine as the variable in fetal samples. *P*-values were adjusted with the Benjamini–Hochberg FDR procedure.

Real-time PCR was performed by using the TaqMan Advanced miRNA assay kit and Applied Biosystems 7900HT Fast Real-Time PCR System following manufacturer’s instructions. The expression level (RE) of each miRNA relative to the small non-coding RNA U6 was calculated using the delta Ct (cycle threshold) method (RE_miRNA = _2^-(Ct miRNA-Ct U6)^) and used to compare control and schizophrenia samples. As the PCR data did not pass the normality and equal variance tests, Mann–Whitney *U*-test was used for statistical analysis.

### Temporal expression patterns of miRNAs and mRNAs

mRNA expression data of our non-schizophrenia samples were downloaded from http://braincloud.jhmi.edu. To obtain each miRNA and mRNA’s temporal expression profiles, all samples were ordered by post-conception age, expressed as log_2_(age in days + 280). The expression levels of miRNAs and mRNAs of each sample were smoothed by averaging samples within 0.3 logarithmic change of age, and then transformed to *z*-scores. The *z*-score was plotted against age. The resulting temporal profiles were used to categorize miRNAs and mRNAs with hierarchical clustering analysis (a method of grouping similar objects into clusters). For clustering by correlation distance, Pearson correlation coefficients between miRNAs or mRNAs were used for clustering with the statistical software R by using the “ward.D2” method (1-pearson coefficient) of the hclust function. For clustering with dynamic time warping (DTW, another method for measuring similarity between two temporal sequences), the dynamic time warping distance was calculated with the R package dwclust and used for hierarchical clustering analysis with the hclust function in R. The number of clusters was selected with the gap statistic (a method to estimate optimal cluster number)^47^. To compare the concordance between the clusters obtained with the correlation-based method and those with the DTW-based method, the clustComp package was used and a weighted bipartite graph was generated to visualize the distribution of miRNAs in the clusters generated by correlation-based and DTW-based method^48,49^. In this graph, the nodes represent clusters and the weight of the edge represents the number of miRNAs in the intersection between any two, connected clusters. The concordance between the clustering patterns obtained from the correlation-based and the DTW-based method was assessed by randomly reordering the expression level of miRNAs across age and then using this new dataset for DTW-based clustering. This procedure was repeated to generate 5000 permutations. Index of Agreement, i.e. the ratio of the number of non-crossing edges to that of crossing edges in the weighted bipartite graph^48,49^, was calculated for the DTW-based clustering of each permutation vs. correlation-based clustering.

### Gene set enrichment analysis

The miRNA groups with >50 members were used to compile Gene Matrix Transposed (GMT) files. All miRNAs expressed in the DLPFC were ranked by their fold changes between schizophrenia and control samples. The GMT and ranking files were uploaded to the online GSEA JNLP Application to test whether each temporally distinct miRNA group is enriched for up- or downregulated miRNAs.

### Correlation between miRNA and mRNA expression

The mRNA targets of each miRNA were obtained from TargetScan (http://www.targetscan.org). The expression levels of identified target mRNAs in the same samples used for the analysis of miRNA temporal dynamics were downloaded from http://braincloud.jhmi.edu/. Pearson’s correlation coefficients for the expression level of each miRNA and target mRNA pair were calculated across all samples.

### Analysis of miRNA target genes

#### Target enrichment analysis

To identify genes co-regulated by a group of miRNAs (CoR genes), the miRNA-target pairs were retrieved from TargetScan (http://www.targetscan.org). Fisher’s exact test using the following equation was performed to identify protein-coding genes enriched with miRNA binding sites (miRNA response elements; MREs) for the miRNAs in the group (CoR genes; *p*-values were adjusted with the Benjamini-Hochberg FDR procedure; adjusted *p* < 0.05 considered significant). $$P_{{\mathrm{gene}}} = \frac{{\left( {\begin{array}{*{20}{c}} {n_{11} + n_{21}} \\ {n_{11}} \end{array}} \right)\left( {\begin{array}{*{20}{c}} {n_{12} + n_{22}} \\ {n_{12}} \end{array}} \right)}}{{\left( {\begin{array}{*{20}{c}} {n_{11} + n_{12} + n_{21} + n_{22}} \\ {n_{11} + n_{12}} \end{array}} \right)}}$$, where

$n_{11} =$ the number of sites binding to miRNAs belonging to the group in the gene being tested;$n_{12} =$ the number of sites binding to miRNAs not belonging to the group in the gene being tested; $n_{21} =$ the number of sites binding to miRNAs belonging to the group in all genes that are not being tested; $n_{22} =$ number of sites binding to miRNAs not belonging to the group in all genes that are not being tested.

For genes with multiple transcripts, each transcript was subjected to the test individually. A gene was considered a CoR gene if one or more of its transcripts was enriched with MREs for the miRNAs in a group and it is targeted by at least 2 of these miRNAs in the same group.

#### Assignment of genes associated with schizophrenia and synapses

The following gene sets were compiled from published studies:

1. Genes associated with schizophrenia by GWAS. We identified these genes from the genome-wide association studies of the Psychiatric Genomics Consortium (PGC) which identified 108 genomic loci met genome-wide significance and genes in these loci were identified^34^. We removed genes encoding non-coding RNAs from the identified genes.
2. Genes in copy number variation (CNV) regions associated with schizophrenia. We identified genes in CNVs associated with schizophrenia from a series of genome-wide association studies from 2008 to the present^50–60^. These studies used standardized diagnosis, genome-wide detection, confirmation of structural variations using at least two different methods for discovery and validation, and provided sufficient information to identify duplicate samples. Identified CNVs were rare (<1% frequency in the total samples) and regardless of size. We identified the CNV regions associated with schizophrenia reported in these studies, and downloaded RefSeq genes in these genomic regions from the UCSC genome browser using the NCBI36/hg18 or the GRCH37/hg19 assembly as used in the original study. Protein-coding genes identified from ≥2 studies were included in our gene set.
3. Genes containing rare de novo single nucleotide variants (SNVs) associated with schizophrenia. Rare de novo mutations in patients were identified from exome sequencing studies of families with schizophrenia patients (trios and/or quads)^61–64^ that had validated the putative mutations with independent methods. We selected only the de novo mutations from patients and excluded synonymous and silent mutations. Only protein-coding genes harboring the selected SNVs were used to build our gene set.
4. Synaptic genes. Human PSD gene set^65^, mouse PSD-95 complex^66^, mouse NMDAR complex^50^, mouse ARC complex^50^, rat mGluR5 complex^67^, rat presynaptic vesicle proteins and rat presynaptic plasma membrane proteins^68^ were compiled from published studies. Rat and mouse genes were matched to human orthologs using the HOM_AllOrganism.rpt file downloaded from the Mouse Genome Informatics database. The GI numbers of the human orthologs were converted to Entrez gene IDs using the “Retrieve/ID mapping tool” at uniport (http://www.uniprot.org/uploadlists/).

#### Enrichment of CoR genes in schizophrenia risk genes and synaptic genes

The *p*-values derived from the target enrichment analysis were used to generate cumulative probability plots for all target genes of miRNAs expressed in the DLPFC, and genes in each schizophrenia risk and synaptic protein gene set. The cumulative probability plot of each gene set was compared to that of all genes targeted by miRNAs expressed in the DLPFC using Kolmogorov–Smirnov test and *p* < 0.05 was considered significant.

#### GO terms and signal transduction pathways enriched by CoR genes

GO terms and signal transduction pathways associated with genes enriched in the target genes of miRNAs were retrieved from the Genomatix Genome Analyzer (GGA) database (Genomatix, Germany). All human genes containing miRNA binding sites predicted by TargetScan were used as background in the enrichment analysis. *P*-values were adjusted from the results of 1000 simulated null hypothesis queries, and *p* < 0.01 was considered significant.

## Results

### Temporal dynamics of miRNA expression in the human DLPFC

To characterize the temporal dynamics of miRNA expression in the human brain, we prepared miRNAs from the Brodmann area 46 of the DLPFC of subjects without mental illnesses (second trimester to 74 years of age, demographics in Supplementary Table 1), and analyzed miRNAs using NGS. Because the small RNA fraction that we isolated for miRNA analysis has very low levels of RNAs ≥200 bases, the RNA integrity number (RIN, an index of RNA quality based on the degradation of ribosomal RNAs) could not be obtained. However, because the mean post-mortem interval (PMI) of our brain samples is 26.9 ± 17.9 h (Supplementary Table 1), which is within the 96-h window when miRNAs exhibit high stability due to their superior resistance to low and high pH, extended storage, freeze-thaw cycles, and RNase^69–72^, our miRNA samples should have sufficient quality for NGS analysis.

An average of 13.9 million quality-filtered reads was obtained from each sample. The length distribution of the reads had a major peak around 22 nt (Supplementary Fig. 1a). This distribution pattern is in line with that of known human miRNAs (Supplementary Fig. 1b). 73.8% of the reads from our libraries were mapped to mature human miRNAs, and a total of 1074 mature miRNAs with ≥10 mapped reads in each sample were identified as miRNAs expressed in the DLPFC. This miRNA number is consistent with those reported by previous human brain studies^10,73,74^. The outliers in mapped reads and mapping rate (Supplementary Fig. 1c, d) still had >410,000 mapped reads (>400 times of detected miRNA species) and human postmortem samples are precious, we therefore included these samples for further analysis.

**Fig. 1:**
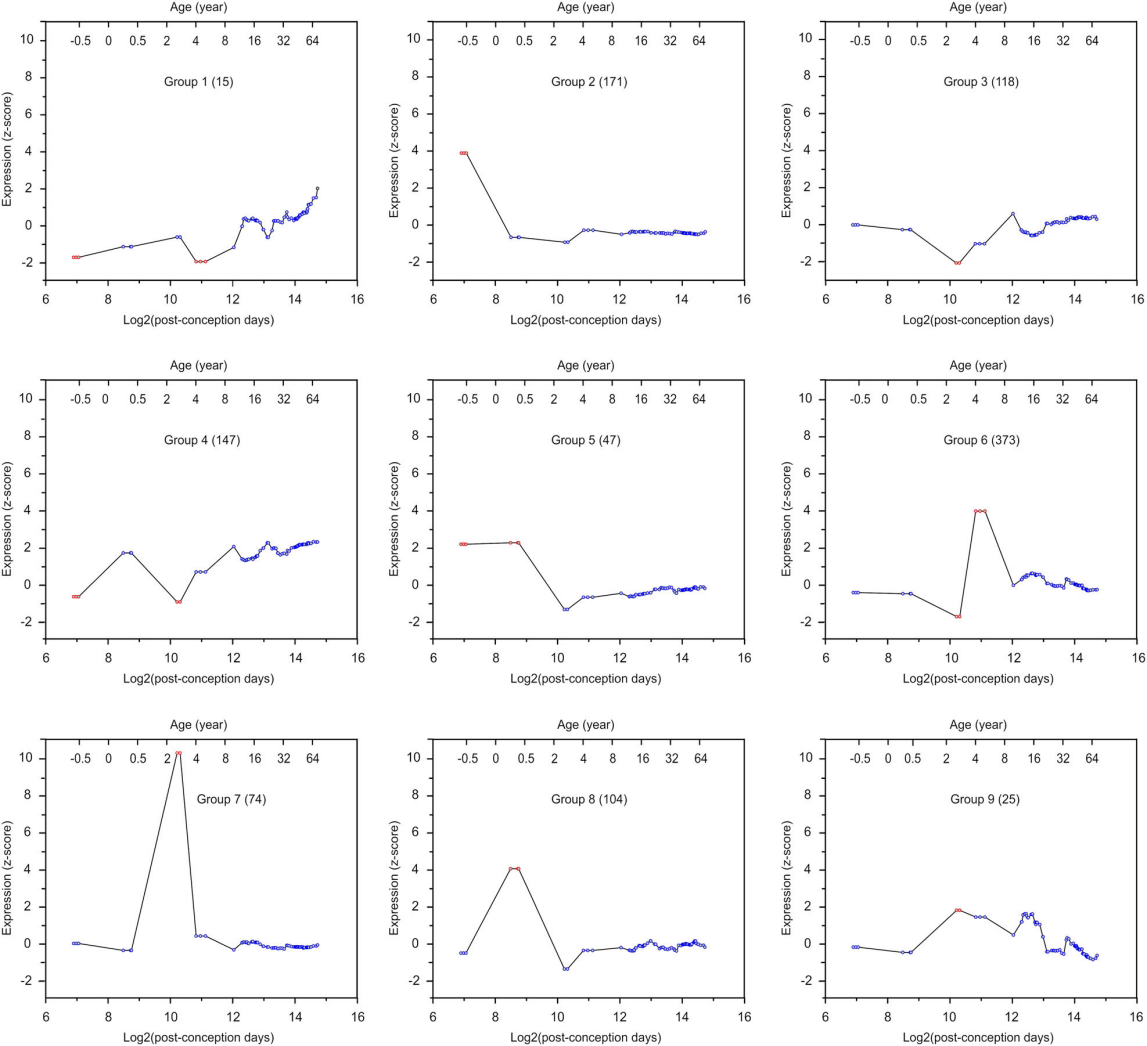
The temporal dynamics of miRNAs expressed in the DLPFC. Histograms show each miRNA group’s mean miRNA expression vs. age. Each circle represents individual samples, and red circles indicate samples with significantly different expression (*p* < 0.05 and with ≥2 samples at the same developmental stage; *p*-values were converted from *z*-scores). The title of the histogram indicates the name of each miRNA group and the number of miRNAs in each group

We derived each miRNA’s temporal expression profile by plotting all samples’ miRNA expression levels against post-conception age. To classify miRNAs by their temporal expression patterns, we used correlation-based hierarchical clustering analysis (see Materials and Methods for details). This analysis identified nine miRNA groups (Fig. 1, Supplementary Fig. 2). To identify time points with significantly different miRNA expression within each group, we converted the *z*-scores of miRNA expression levels to *p*-values. Only developmental stages with *p* < 0.05 and ≥2 samples were considered significant (depicted by red circles in Fig. 1, Supplementary Fig. 2). All nine miRNA groups had significant expression changes before puberty: Group 1 had lower expression in fetuses and preschoolers, Group 2 had higher expression in fetuses; Group 3 were expressed at a lower level in toddlers; Group 4 had lower expression in fetuses and toddlers; Group 5 had stable and higher expression in fetuses and infants, Group 6 had a wave of expression between infants and preschoolers; Groups 7 and 9 had a peak of expression in toddlers; Group 8 had a peak of expression in infants (Fig. 1 and Supplementary Fig. 2).

To validate the pattern obtained with the correlation-based hierarchical clustering, we re-clustered miRNAs by temporal dynamics using dynamic time warping (DTW) and generated a weighted bipartite graph to visualize the relationship between clusters obtained from the two clustering methods^48,49^ (Supplementary Fig. 3a, b). In this graph, the nodes represent clusters and the weight of the edge represents the number of miRNAs in the intersection between any two, connected clusters. The proportion of genes overlapping between correlation-based and DTW-based clusters was illustrated in Supplementary Fig. 3c. The concordance between the two clustering patterns was assessed by randomly reordering the expression level of miRNAs across age and then using this new dataset for DTW-based clustering. This procedure was repeated to generate 5,000 permutations. Index of Agreement, i.e. the ratio of the number of non-crossing edges to that of crossing edges in the weighted bipartite graph, was calculated for the DTW-based clustering of each permutation vs. correlation-based clustering. The Index of Agreement for the correlation-based and the DTW-based clusters was significantly higher than that for permutations (Supplementary Fig. 3d), suggesting consistency between the two clustering methods.

### Temporal dynamics of mRNAs targeted by miRNAs

**Fig. 2:**
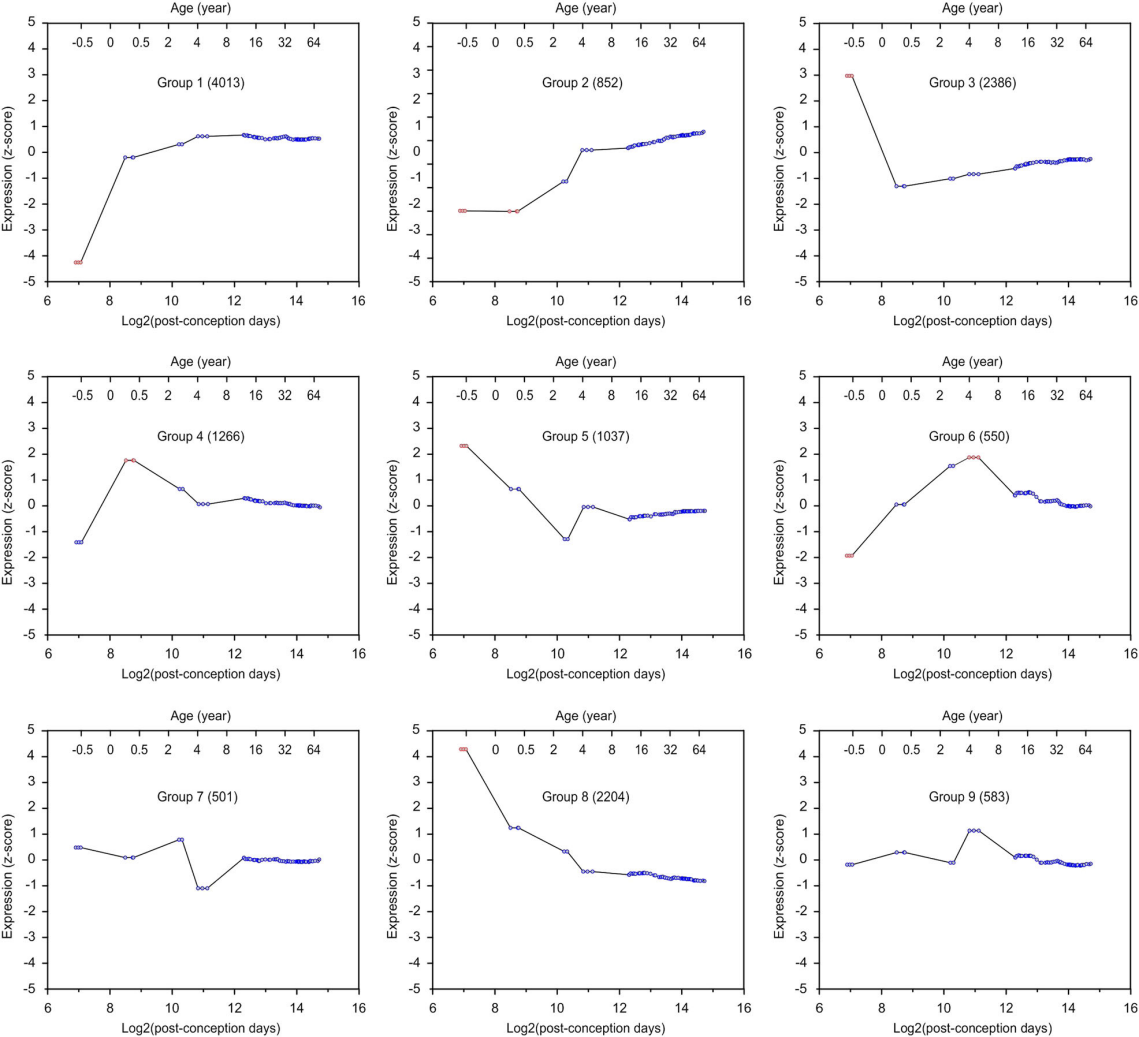
The temporal dynamics of mRNAs expressed in the DLPFC and with miRNA binding sites. Histograms show each mRNA group’s mean mRNA expression vs. age. Each circle represents individual samples, and red circles indicate samples with significantly different expression (*p* < 0.05 and with ≥2 samples at the same developmental stage; *p*-values were converted from *z*-scores). The title of the histogram indicates the name of each mRNA group and the number of mRNAs in each group

**Fig. 3:**
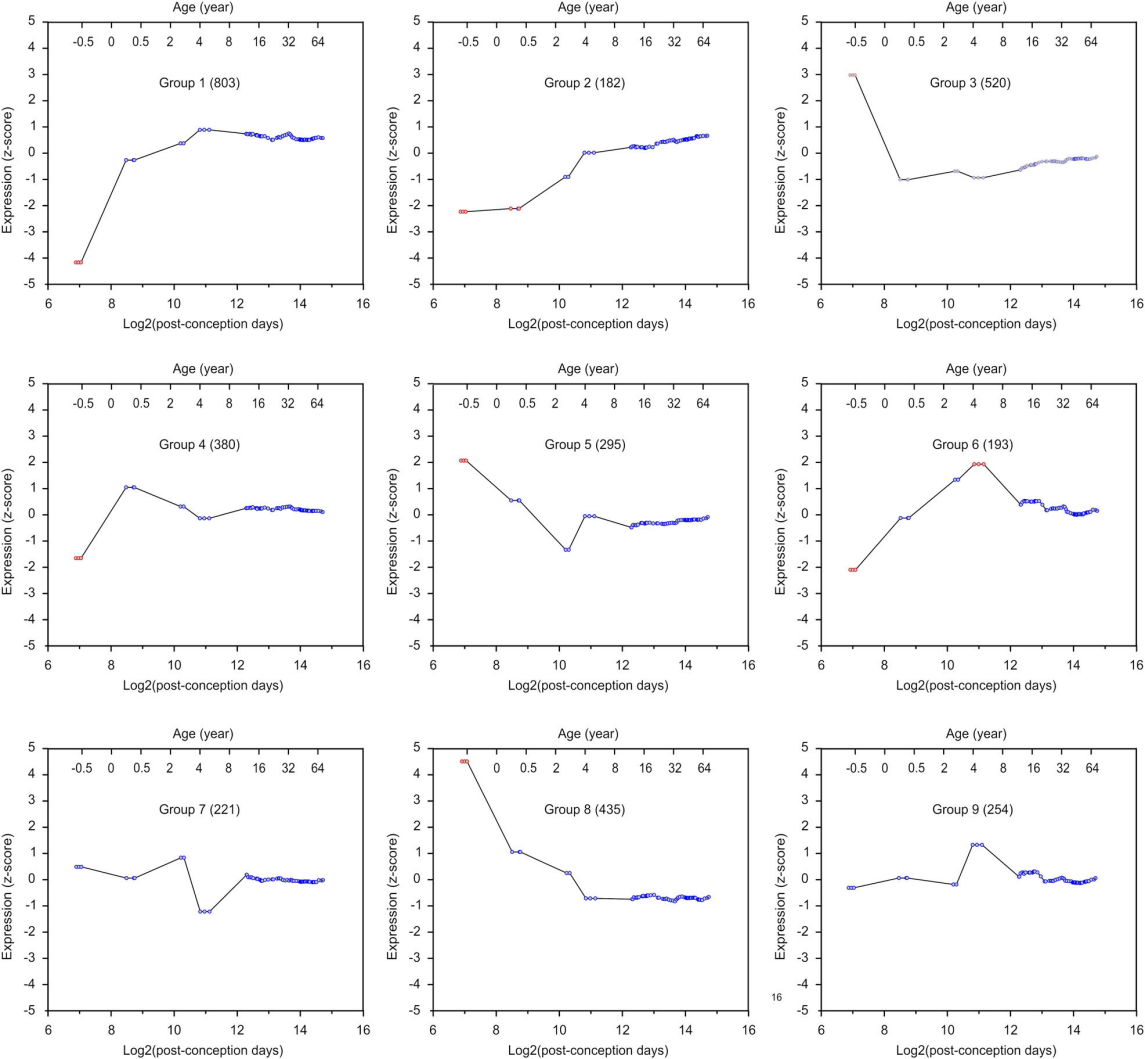
The temporal dynamics of mRNAs expressed in the DLPFC and without miRNA binding sites. Histograms show each mRNA group’s mean mRNA expression vs. age. Each circle represents individual samples, and red circles indicate samples with significantly different expression (*p* < 0.05 and with ≥2 samples at the same developmental stage; *p*-values were converted from *z*-scores). The title of the histogram indicates the name of each mRNA group and the number of mRNAs in each group

Since miRNAs regulate mRNA expression, we examined whether mRNAs have similar temporal dynamics as miRNAs. mRNA expression in the same DLPFC samples used in the above miRNA analysis has been previously measured^2^. We downloaded this dataset (http://braincloud.jhmi.edu/) to determine the temporal profile of mRNA expression using hierarchical clustering. mRNAs with and without computationally predicted miRNA binding sites were separately analyzed. mRNAs containing computationally predicted miRNA binding sites were classified into 9 groups: 73.7% mRNAs (Groups 1, 3, 4, and 8) exhibited the biggest expression change during the transition from fetal to postnatal development, Group 2 increased from birth to toddler age, and Group 5 decreased from fetal to toddler stage, Group 6 increased expression until reaching the peak in preschoolers (Fig. 2, Supplementary Fig. 4). mRNAs without predictive miRNA binding sites exhibited the same temporal expression profiles and distribution across groups (Fig. 3, Supplementary Fig. 5). This analysis showed that mRNA expression is also coupled to age as miRNAs, and most mRNAs changed their expression during the perinatal period.

It is known that miRNAs affect the protein production of their targets by translation inhibition or mRNA cleavage. The latter mechanism is relatively modest and has a subtle effect on the mRNA level^4^, which in fact is predominantly regulated by transcription and mRNA degradation. The expression levels of miRNAs and mRNAs, therefore, do not necessarily correlate. Nevertheless, we examined the correlation between miRNAs and their target mRNAs in the same brain samples using Pearson’s correlation coefficient analysis.

Of all miRNA–target mRNA pairs, 0.40% of them were positively correlated (*r* > 0.49, FDR < 0.05; Supplementary Table 2), and 0.29% of them were negatively correlated (*r* < −0.49, FDR < 0.05; Supplementary Table 2). To test whether these low percentages are true or due to chance, we randomly reshuffled the label of our samples and conducted the correlation test again. The resulting percentage of miRNA–target mRNA pairs that were correlated was ~2 orders of magnitude lower than that from the original analysis (Supplementary Table 3), indicating that the observed low percentage of correlation between miRNAs and mRNAs is unlikely due to chance. This finding is indeed consistent with previous reports that endogenous miRNAs have only subtle effects on mRNA levels and only a small fraction of miRNA–target mRNA pairs are correlated in expression levels^75–77^.

It is noted that 20 of the 24 miRNA–target mRNA pairs with *r* < −0.7 are miR-206–target pairs (Supplementary Table 2). The mRNAs in these pairs are involved in cellular processes essential for brain development and function such as transcription, cytoskeleton remodeling, neurotransmitter release, and neuropeptide signaling. VAMP2, one of the mRNAs highly correlated with miR-206 (*r* = −0.71, FDR = 3.42 × 10^−11^) is indeed a validated miR-206 targets^78,79^. Taken together, these findings indicate that mRNAs and miRNAs have different temporal expression profiles and that only a small proportion of miRNAs correlate with their target mRNAs at expression levels.

### miRNA expression changes in the DLPFC of schizophrenia patients

Having identified the temporal dynamics of miRNAs, we proceeded to test whether miRNAs’ expression changes in schizophrenia patients are related to their temporal features. To this end, we extracted miRNAs from the Brodmann area 46 of 34 schizophrenia patients (demographics in Supplementary Table 4), and used the ≥18-year-old subjects in the above developmental study as controls.

**Fig. 4:**
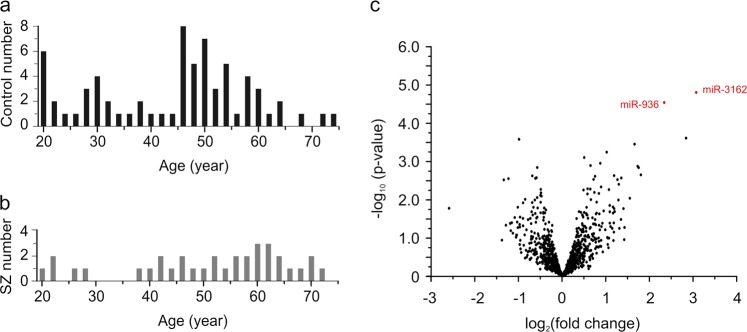
miRNA expression changes in the DLPFC of schizophrenia cases. miRNAs extracted from the DLPFC of control and schizophrenia cases were analyzed using NGS. **a**, **b** Subject numbers. **c** The fold change (case vs. control) of each miRNA is plotted against its *p*-value; red circles represent miRNAs with FDR < 0.05

Our brain samples were comprised of males, females, Caucasians and African Americans, and varied in age (18–74 years), pH (5.9–7.1), and post-mortem interval (PMI; 34.2 ± 21.8 hours on average) (Supplementary Table 4). We, therefore, tested whether any of these variables confound miRNA expression using principal component analysis (PCA). PCA, however, did not reveal clear segregation (Supplementary Fig. 6), suggesting that none of these variables dominated our samples’ miRNA expression profiles. It is not a surprise that the brain samples also did not cluster by diagnosis (Supplementary Fig. 6) since schizophrenia has complex symptomatology (e.g. positive and negative symptoms) and etiology (attributable to both genes and environments). The lack of clear segregation by demographic factors, however, does not exclude their influence on miRNA expression. Hence, we employed the general linear model (GLM), adjusting for diagnostics, race, gender, and age (young adult: 18–39 years; adult: 40–60 years; senior:>60 years), to search for miRNAs differentially expressed in schizophrenia cases. In addition, since our samples were prepared as two batches during the preparation of miRNA libraries for NGS, and the first PC of the control samples was strongly associated with batch (*p* < 6.38 × 10^−6^), we also adjusted for batch in the GLM. GLM analysis identified two miRNAs, miR-3162 and miR-936, that passed the statistical test, and both increased in schizophrenia cases (*p* < 3 × 10^−5^, FDR < 0.03; Fig. 4, Table 1).

**Table 1:** miRNAs differentially expressed in schizophrenia cases and the effect of psychiatric medications on their expression

			hsa-miR-3162	hsa-miR-936
Temporal group			5	3
SZ vs. control		Log_2_ (fold change)	2.89	2.27
		*p*-value	0.000023	0.000042
		FDR	0.022	0.022
CPZE	Lifetime CPZE	Log_2_ (fold change, high vs. low dose)	−1.63	−0.01
		Log_2_ (fold change, high vs. medium dose)	−2.76	−2.61
		*p*-value	0.12	0.030
		FDR	0.42	0.25
	Last CPZE	Log_2_(fold change, high vs. low dose)	1.41	0.32
		Log_2_(fold change, high vs. medium dose)	0.17	2.55
		*p*-value	0.35	0.02
		FDR	0.87	0.62
	Daily CPZE	Log_2_ (fold change, high vs. low dose)	−1.69	−2.84
		Log_2_ (fold change, high vs. medium dose)	−3.15	−3.74
		*p*-value	0.016	0.0014
		FDR	0.68	0.26
SSRI		Log_2_ (fold change)	0.24	−0.69
		*p*-value	0.79	0.57
		FDR	0.95	0.88

**Fig. 5:**
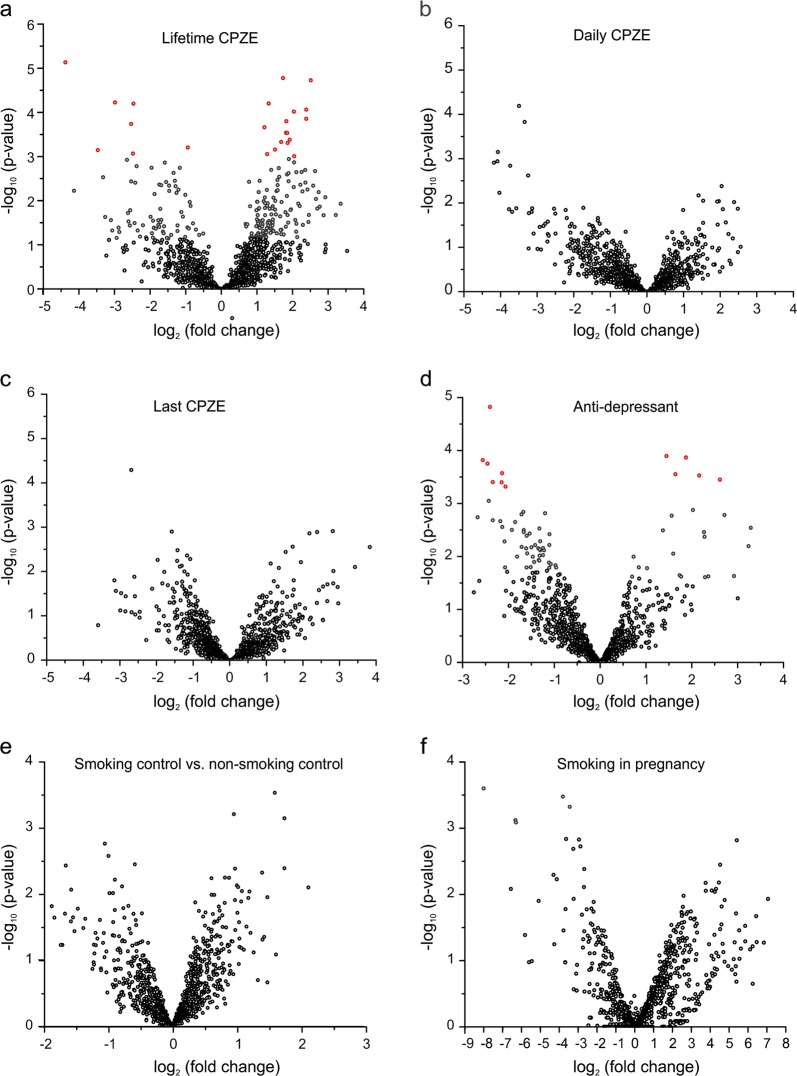
The effects of psychiatric medications and smoking on miRNA expression in the DLPFC. miRNAs extracted from the DLPFC of control and schizophrenia cases were analyzed using NGS. The fold change (the greater one of high vs. low dose and high vs. median dose in **a**–**c**, medicated vs. non-medicated in **d**, smoking vs non-smoking in **e**, **f** of each miRNA is plotted against its *p*-value. Red circles represent miRNAs with FDR < 0.05

All of our patients have taken psychiatric medications such as antidepressants and antipsychotics, and tobacco smoking is more prevalent in schizophrenia patients than in the general population^80^. To test whether these factors affect miRNA expression, we compared the effect size of miRNA expression change calculated from the original GLM model (adjusting for age, gender, race, and batch) to that including antipsychotics, antidepressants, or smoking. From this sensitivity analysis, it appears that miRNA expression is affected by psychiatric medications (Supplementary Fig. 7). We, therefore, analyzed for drug effects using GLM. Only schizophrenia samples were included in this analysis because control subjects did not take psychiatric medications. To test for the antipsychotics effect, patients were divided into three groups by lifetime, daily or last dose of chlorpromazine (CPZE) equivalents: high (on the top one-third of the dose range taken by our patients), medium (on the middle one-third dose) and low (on the bottom one-third dose). This analysis showed that miR-3162 and miR-936 were not affected by antidepressants or the dose of antipsychotics (Table 1, Fig. 5a–d). We used real-time PCR to confirm the expression change of miR-3162 and miR-936 in schizophrenia samples. In 5 control and 5 schizophrenia samples matched in age, gender and race (Supplementary Table 5), both miR-3162 and miR-936 increased, while miR-124 was unchanged (Supplementary Fig. 8).

The effect of smoking on miRNA expression was analyzed in control, adult samples (>18 years of age, adjusting for age, sex, and race) and fetal samples (using cotinine, a nicotine metabolite, as the variable). Smoking had no significant effect on miRNA expression in adult or fetal brains (Fig. 5e, f).

Taken together, these findings indicate that miR-3162 and miR-936 are upregulated in schizophrenia patients. miR-3162 belongs to Group 5 miRNAs which are enriched in fetuses and infants, while miR-936 is a member of Group 3 which is expressed at a lower level in toddlers (Table 1, Fig. 1).

### Infant-enriched miRNAs show a concordant increase while prepuberty-enriched miRNAs show a concordant decrease in schizophrenia patients

The traditional miRNA differential analysis we used is focused on individual genes. This single-gene approach is successful in identifying genes with large fold changes and modest variability among samples. However, for genes with modest fold changes relative to the noise of the gene expression detection technology, the high biological variations between individuals and the limited sample size inherent to postmortem human brain studies make this approach difficult to distinguish true differences from noise^81^. Using the single-gene approach, after correction for multiple hypotheses testing, there are often no or only a few genes that meet the threshold for statistical significance, which may cause poor overlap between different studies^82,83^. In addition, since cellular processes often have a modest effect on sets of genes acting in concert rather than a strong effect on a single gene, the single-gene analysis may miss the biological relevance of gene expression change.

**Fig. 6:**
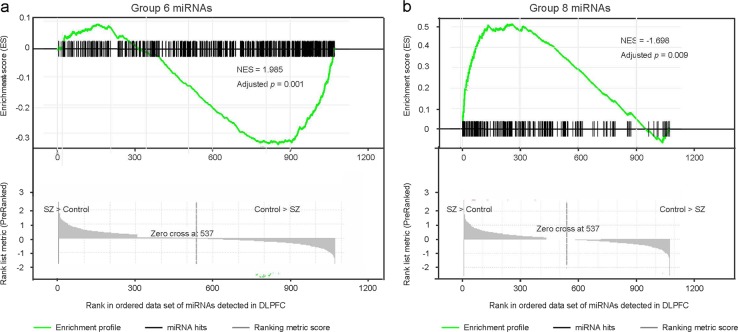
miRNAs preferentially expressed during infancy are enriched for miRNAs upregulated in schizophrenia patients, while miRNAs preferentially expressed during prepuberty are enriched for those downregulated in schizophrenia patients. miRNAs preferentially expressed during prepuberty (Group 6; **a**) and infancy (Group 8; **b**) are ranked by their fold changes in schizophrenia cases (from increase to decrease) for gene set enrichment analysis. NES, normalized enrichment score

Because of these considerations, methods of analyzing expression at the gene-set level have been developed. Gene set enrichment analysis (GSEA) is such a method that is widely used. It evaluates whether a priori defined set of genes shows statistically significant, concordant expression changes between two biological conditions^81,84^. For GSEA, a list of genes is ranked by their fold changes regardless of the *p*-value for individual genes and an enrichment score is calculated to determine whether a gene set is enriched at the top or bottom of the gene list. To complement our miRNA differential expression analysis, we used GSEA to examine whether miRNAs with similar temporal dynamics have concordant alterations in schizophrenia patients. To this end, we considered each miRNA group as a gene set. Because miRNA groups with a small number of members (≤50) can have high variation across different cohorts, we focused on groups with >50 miRNAs in this analysis. A rank list of miRNAs was generated by ordering all miRNAs expressed in the DLPFC with their fold changes (from positive to negative). GESA showed that Group 6 miRNAs (peaking during prepuberty) were enriched for miRNAs downregulated in schizophrenia cases (normalized enrichment score = −1.70, *p* = 0.012, FDR = 0.009; Fig. 6a), while Group 8 miRNAs (peaking during infancy) were enriched for miRNAs upregulated in schizophrenia patients (normalized enrichment score = 1.99, *p* = 0, FDR = 0.001; Fig. 6b). The other groups were not significantly enriched on either side of the rank list. These findings indicate that the DLPFC of schizophrenia patients tend to up-regulate miRNAs preferentially expressed in normal infant brains and down-regulate those enriched in normal prepubescent brains.

### Genes co-regulated by Group 6 and Group 8 miRNAs

miRNAs regulate their target genes’ expression via translational repression or mRNA destabilization. Each mRNA can be targeted by multiple miRNAs. While the effect of one miRNA on gene expression is moderate, different miRNAs targeting the same mRNA exert synergistic effects^6^. To gain a comprehensive perspective on the genes regulated by Group 6 and Group 8 miRNAs, we searched for target genes that are enriched by each of the two groups. To this end, we retrieved the target genes of all miRNAs expressed in the DLPFC from the TargetScan database (http://www.targetscan.org/). TargetScan was selected because it offers the best balance of sensitivity and precision. Although using the intersection of multiple target prediction algorithms might improve the precision, this approach usually performs worse than a single algorithm due to the higher price paid for the sensitivity^85–87^. After target identification, we used the hypergeometric test to search for genes that were co-targeted by at least two of miRNAs in the same group (Group 6 or Group 8) and over-represented by the targets of miRNAs in each group. These genes were named co-regulated (CoR) genes (Supplementary Tables 6 and 7). Because individual miRNAs have a moderate effect on gene expression, genes co-targeted by ≥2 miRNAs of the same group are more likely to reflect the biological relevance of miRNAs in one temporal group. Group 6 and Group 8 had one CoR gene in common. The overlap between CoR genes of the 9 miRNA groups is illustrated in Supplementary Fig. 9a.

To systematically annotate the CoR gene’s function, we used the Genomatix database to search for Gene Ontology (GO) terms and signal transduction networks enriched by CoR genes. CoR genes of Group 6 and Group 8 miRNAs were subjected to the enrichment analysis separately, and all human genes with miRNA binding sites predicted by TargetScan were used as background. This analysis showed that the CoR genes are over-represented in GO terms associated with cellular activities and signal transduction networks involved in neuronal development and function, such as glycogen synthase kinase, dendritic development, ion channel activity, synaptic vesicle exocytosis, and neurotransmitter activity (Supplementary Tables 8–13). Since Group 6 and Group 8 miRNAs can be expressed in both neuronal and non-neuronal cells, and they are not necessarily only expressed in infants or prepubescents, it is not a surprise that their CoR genes are also enriched in GO terms and signal transduction networks associated with cellular processes not specific to neurons or the infant and prepubescent periods, such as transcription, translation, neurogenesis, and neural differentiation.

GO terms and signal transduction networks are broadly defined, and therefore can be targeted by multiple miRNA groups. The overlap of enriched GO terms and signal transduction networks between miRNA groups are shown in Supplementary Fig. 9b–d. The gene list associated with GO terms and signal transduction networks is still evolving to incorporate new biological discoveries and knowledge. The GO term enrichment analysis, therefore, offers a preliminary insight into the biological processes potentially regulated by the temporal miRNA groups.

### Targets of Group 6 and Group 8 miRNAs are enriched for genes associated with schizophrenia

Many genes have been linked to the risk of schizophrenia. Having found that Group 6 and Group 8 miRNAs tend to be dysregulated in schizophrenia patients, we tested whether these miRNAs’ target genes are associated with schizophrenia. To this end, we identified genes associated with schizophrenia by reviewing the literature and assigned them into three gene sets: genome-wide association study (GWAS), copy number variant (CNV), and de novo single-nucleotide variant (SNV).

For each gene set, a hypergeometric test was used to calculate its probability of being targeted by Group 6 and Group 8 miRNAs. This analysis revealed that these miRNAs more likely target the GWAS and SNV gene sets (Table 2). These findings suggest that Group 6 and Group 8 miRNAs regulate the expression of genes associated with schizophrenia.

**Table 2:** Enrichment of genes targeted by Group 6 and Group 8 miRNAs in gene sets associated with schizophrenia

Gene Set	Gene # in the gene set	Genes targeted by all miRNAs	*P*-value for Group 6 miRNAs	*P*-value for Group 8 miRNAs
SNV	240	193	0.0480	0.00315
CNV	64	56	0.84	1.00
GWAS (PGC)	281	209	0.042	0.0076

### Targets of Group 6 and Group 8 miRNAs are enriched for synaptic proteins

Synaptic connections in the brain undergo maturational and refinement processes during the infant and prepubescent periods. Synaptic proteins are essential for these developmental processes and have been implicated in the pathophysiology of schizophrenia. For instance, recent genetic studies show that de novo CNVs and genetic mutations in schizophrenia patients are enriched for proteins in postsynaptic signaling complexes^50,88,89^. To test for the potential impact of Group 6 and Group 8 miRNA dysregulation on synapses, we obtained the following synaptic gene sets from reported proteomic studies: postsynaptic density (PSD) complex^65^, PSD-95 complex^66^, *N*-methyl-d-aspartate receptor (NMDAR) complex^50^, activity-regulated cytoskeleton-associated protein (Arc) complex^50^, metabotropic glutamate receptor 5 (mGluR5) complex^67^, presynaptic plasma membrane proteins and synaptic vesicle proteins^68^. Using the same enrichment analysis as for the schizophrenia-associated gene sets, we found that the targets of Group 6 and Group 8 miRNAs are enriched for the PSD, NMDA receptor, mGluR5, Arc, and synaptic vesicle complexes (Table 3). Hence, miRNAs preferentially expressed during infancy and prepuberty regulate genes encoding synaptic proteins.

**Table 3:** Enrichment of genes targeted by Group 6 and Group 8 miRNAs in synaptic proteins

Gene set	Gene # in the gene set	Genes targeted by all miRNAs	*P*-value for Group 6 miRNAs	*P*-value for Group 8 miRNAs
mGluR5 complex	45	45	0.01	0.04
PSD-95 complex	60	56	0.22	0.11
PSD	748	676	5.10E-11	1.02E-08
NMDAR complex	61	60	0.004	0.002
ARC complex	29	27	0.002	0.01
Presynaptic plasma membrane	85	81	0.05	0.99
Synaptic vesicle	75	70	3.95E-06	2.42E-04

## Discussion

In this study, we characterized the temporal dynamics of miRNA expression in the human DLPFC and identified miRNAs altered in schizophrenia subjects. Our analyses show that most miRNAs in the DLPFC exhibit expression peaks or waves before puberty and stay at relatively stable levels afterward. This temporal feature is different from that of mRNAs which has a wave of change during the perinatal period^2,90^.

Being important fine tuners in the gene expression network, miRNAs are not only essential for normal brain development but also related to the pathophysiology of schizophrenia^5,24,91^. Using microarray and quantitative PCR, while a number of groups have detected miRNA expression changes in the postmortem brains of schizophrenia patients^23^, their findings are largely non-overlapping. The discrepancies can arise from technical variations in RNA isolation, amplification, and array platforms, as wells as from differences in data analysis, such as selection of internal references for normalization (global mean normalization vs. normalization to small-nucleolar RNAs), study design (cohort vs. case-control study), and stringency of statistical analysis (whether or not adjusting *p*-values for multiple comparisons). In addition, biological variations including differences in brain regions, demographics, disease etiology, symptomology, and medical history of brain donors can have a significant impact on the results.

NGS technology is more sensitive and quantifiable compared to microarray^92^. A recent study using NGS to analyze miRNA expression in the anterior cingulate cortex of schizophrenia patients, however, does not find alterations of miRNA expression^93^. Here, we used NGS to delineate miRNA expression changes in schizophrenia and the effects of psychiatric medications on miRNA expression. Although our analysis was limited by the lack of antipsychotics-free cases, comparing cases taking different doses enabled us to identify, at least some of, the miRNAs that respond to antipsychotics. After considering the drug effect, we found that miR-3162 and miR-936 increased in schizophrenia cases. Neither genetic association nor expression changes related to schizophrenia have been previously reported for these miRNAs. This is not a surprise given the poor overlap of published results from genetic and expression studies of schizophrenia^94,95^. Since we did not find effects of psychiatric medications on the expression of miR-3162 and miR-936, their overexpression in schizophrenia patients is likely caused by the disease. However, we cannot exclude potential drug effects due to the limited sample size and lack of neuroleptics-free samples. A direct test for the effect of neuroleptics on miR-3162 and miR-936 in human brains is not feasible. Since miR-3162 and miR-936 are expressed only in primates, future studies with more postmortem human brains and non-human primates can be used to address this question and elucidate the underlying mechanism.

It is noted that Group 8 miRNAs, miR-3162, and miR-936 which normally are enriched in infant, fetal, or toddler brains are upregulated, while Group 6 miRNAs, which normally have a peak of expression during prepuberty and are at higher levels in adults than in toddlers, are downregulated in the DLPFC of adult, schizophrenia patients. These alterations could be due to a defective progression of brain development in infants, which gives rise to an infant-like pattern of miRNA expression in adulthood. It is also possible that miRNAs in the DLPFC revert to their infant or toddler pattern of expression after the onset of schizophrenia. Both scenarios suggest that the brains of adult schizophrenia patients and normal immature subjects are similar in miRNA expression. This feature implicates anomalous brain development in the pathogenesis and/or pathophysiology of schizophrenia, which is consistent with the developmental theory of schizophrenia^96,97^. This notion is further supported by the enrichment of schizophrenia-associated genes in Group 6 and Group 8 miRNAs, a finding consistent with a recent report that schizophrenia risk genes are more likely to be targeted by miRNAs^39^.

Our temporal profiling shows that in Brodmann’s area 46 of human DLPFC, most miRNAs are predominantly expressed and exhibit expression changes before adolescence and become relatively stable afterward. This is in line with the previous findings that global miRNA expression in Brodmann’s area 46 is highest before adolescence and that a number of miRNAs are altered in expression during the early years^8,9,98^. Of the nine temporal groups of miRNAs identified in our study, Group 6 which has the greatest number of miRNAs has an expression peak in preschoolers. This finding is distinct from that in Brodmann’s area 11 which shows that the greatest number of differentially expressed miRNAs is between infants and toddlers^98^. The different findings in Brodmann’s area 11 and 46 may result from the region-specific developmental schedule and gene expression pattern in the brain.

The miRNA–target mRNA pair analysis has several limitations. First, the target mRNAs are based on computational prediction. Although this is a powerful and comprehensive discovery method, the predicted targets need experimental validation. Second, although miRNAs can destabilize mRNAs, because this mechanism has a subtle effect on the mRNA level^4^, and one mRNA can be targeted by multiple miRNA species, the expression levels of miRNAs and mRNAs do not necessarily correlate. Third, miRNAs and mRNAs were isolated from brain samples containing a mixture of different cell types, which may use different mechanisms to control miRNA and mRNA expression and have different relationships between miRNA and target mRNA expression. It is impossible to have the same cell type composition in all brain samples. Even when we compared miRNAs and mRNAs isolated from the same brain donor, the heterogeneity of cell type composition in brain samples contributes to the noise of the miRNA–target mRNA pair analysis.

The analysis of GO terms and signal transduction networks associated with miRNAs are also limited by the miRNA target identification method. Additionally, the assignment of genes to GO terms and signal transduction networks is based on biological knowledge which is incomplete and constantly evolving. The biological functions associated with individual miRNAs based on GO term and signal transduction network analysis, therefore, is only the first step towards understanding miRNA functions.

A single miRNA can target numerous genes and multiple miRNAs can regulate gene expression in concert. The miRNAome change, therefore, often has pleiotropic effects on cell physiology and behavior^17,18^. We found that the targets of Group 6 and Group 8 miRNAs are enriched for several synaptic protein complexes, which are known to be dysregulated in schizophrenia patients^99^. They also co-target many cellular processes and signal transduction pathways associated with neuronal development and synaptic function. Because Group 6 miRNAs are preferentially expressed during prepuberty and Group 8 miRNAs are enriched in infants, these findings are consistent with the fact that infancy and prepuberty are within the period when synaptic connections are formed and refined. Hence, it is likely that dysregulation of the cellular processes targeted by miRNAs preferentially expressed during brain development can contribute to the structural and functional alterations of patient brains, such as decreased brain volume, abnormal DLPFC activation during working memory, altered glutamatergic and GABAergic neurotransmission and disturbance of the dopaminergic system^100–104^.

In sum, analyzing miRNA expression in postmortem human brains, we identified the temporal features of the miRNAome in normal brains and miRNAs differentially expressed in schizophrenia patients. By comparing the miRNAomes of normal, developing brains with those of schizophrenic brains, our study suggests that the postnatal period before puberty is critical in the development of schizophrenia and that the DLPFC of adult schizophrenia patients remains at or has reverted to immature states. Our study is limited by the small number of samples. A high temporal resolution of miRNA expression profiles and unambiguous conclusions about the effect of neuroleptics on miRNA expression can be determined by future studies with more postmortem human brains.

## Supplementary information

Supplementary figure legend.

Supplementary figure and supplementary table.
