# Correction: Temporal dynamics of miRNAs in human DLPFC and its association with miRNA dysregulation in schizophrenia

**DOI:** 10.1038/s41398-019-0572-9

**Published:** 2019-09-23

**Authors:** Zhonghua Hu, Shouguo Gao, Daniel Lindberg, Debabrata Panja, Yoshi Wakabayashi, Keshen Li, Joel E. Kleinman, Jun Zhu, Zheng Li

**Affiliations:** 1 Section on Synapse Development and Plasticity, National Institute of Mental Health, National Institutes of Health, Bethesda, MD 20892 USA; 2 Systems Biology Center, National Heart Lung and Blood Institute, National Institutes of Health, Bethesda, MD 20892 USA; 3 Institute of Neurology, Affiliated Hospital of Guangdong Medical University, Zhanjiang, Guangdong China; 4 Clinical Neuroscience Institute of Jinan University, Guangzhou, China; 5 Lieber Institute for Brain Development, Baltimore, MD 21205 USA

**Keywords:** Neuroscience, Psychiatric disorders

**Correction to:** **Translational Psychiatry**

10.1038/s41398-019-0538-y published online 20 August 2019

In the original article, author Shouguo Gao’s name was incorrectly stated as “Shuoguo Gao.” This has been updated in the XML, HTML, and PDF versions of this article.

