# Supplementary material for: Temporal dynamics of miRNAs in human DLPFC and its association with miRNA dysregulation in schizophrenia
Source: Transl Psychiatry. 2019 Aug 20;9:196. doi: 10.1038/s41398-019-0538-y (PMC6702224; doi:10.1038/s41398-019-0538-y)
Supplement: Supplementary file 1 — Supplementary figure legend. [file 41398_2019_538_MOESM1_ESM.docx]

**Supplementary Figure Legends**

Supplementary Figure 1. The length, mapped reads and mappability distribution of NGS reads. (a) The proportion of NGS reads obtained from our miRNA libraries at designated length. (b) The proportion of all human miRNAs in miRbase at designated length. (c) Distribution of mapped NGS reads shown as a boxplot. The box spanning the interquartile range covers the second and third quantile, the line in the center of the box is the median, the whiskers indicate 1.5 times the interquartile range, and the open circle indicates samples outside the 1.5 times interquartile range. (d) Distribution of mappability shown as a boxplot.

Supplementary Figure 2. The temporal dynamics of miRNAs expressed in the DLPFC. Histograms show each miRNA group’s mean miRNA expression (black lines) and all miRNAs in the designated group (grey lines) vs. age. Each circle represents individual samples, and red circles indicate samples with significantly different expression (*p* < 0.05 and with ≥ two samples at the same developmental stage; *p*-values were converted from z-scores). The title of the histogram indicates the name of each miRNA group and the number of miRNAs in each group.

Supplementary Figure 3. Classification of miRNAs by temporal dynamics. (a) Classification of miRNAs detected in the human DLPFC using DTW-based hierarchical clustering. (b) Comparison of correlation-based and DTW-based clustering. The nodes represent clusters and the weight of the edge represents the number of miRNAs in the intersection between any two, connected clusters. (c) Overlap between correlation-based and DTW-based clusters. The number in each cell is the percentage of miRNAs in each correlation-based cluster that overlaps with a DTW-based cluster, and the cell color indicates the degree of overlap. (d) Index of Agreement which is the ratio of the number of non-crossing edges to that of crossing edges in C. Index of Agreement for the correlation-based and the DTW-based clustering is illustrated by the blue arrow on the right and that for 5,000 permutations is shown as a distribution plot on the left.

Supplementary Figure 4. The temporal dynamics of mRNAs expressed in the DLPFC and containing miRNA binding sites. Histograms show each mRNA group’s mean mRNA expression (black lines) and all mRNAs in the designated group (grey lines) vs. age. Each circle represents individual samples, and red circles indicate samples with significantly different expression (*p* < 0.05 and with ≥ two samples at the same developmental stage; *p*-values were converted from z-scores). The title of the histogram indicates the name of each mRNA group and the number of mRNAs in each group.

Supplementary Figure 5. The temporal dynamics of mRNAs expressed in the DLPFC and without miRNA binding sites. Histograms show each mRNA group’s mean mRNA expression (black lines) and all mRNAs in the designated group (grey lines) vs. age. Each circle represents individual samples, and red circles indicate samples with significantly different expression (*p* < 0.05 and with ≥ two samples at the same developmental stage; *p*-values were converted from z-scores). The title of the histogram indicates the name of each mRNA group and the number of mRNAs in each group.

Supplementary Figure 6. Principal component analysis (PCA). In each PCA plot, samples are colored based on diagnosis, race, age, gender, batch, pH and PMI. Young adult, 18–39 years; Adult, 40–60 years; senior, > 60 years; AA: African American; CAUC: Caucasian.

Supplementary Figure 7. Sensitivity analysis. The size of miRNA expression changes in schizophrenia cases computed from GLM (adjusting for age, gender, race, and batch as variables (model) was plotted against that including antipsychotics (a), antidepressants (b), and smoking (c).

Supplementary Figure 8. Expression of miR-3162 and miR-936 in matched control and schizophrenia samples. Real-time PCR was used to measure miRNA expression in 5 control and 5 schizophrenia samples. Data are show as mean ± SEM; Mann-Whitney U-test was used to compare control vs. schizophrenia; ** p < 0.01.

Supplementary Figure 9. Overlap between CoR genes, signal transduction networks and GO terms of miRNA groups. Numbers outside the table indicate miRNA groups. The number in each cell represents the percentage of CoR genes, signal transduction networks or GO terms for the miRNA group on the top of the table overlapping with those for the miRNA group to the left of the table. The cell color indicates the degree of overlap.
