# Supplementary material for: Temporal dynamics of miRNAs in human DLPFC and its association with miRNA dysregulation in schizophrenia
Source: Transl Psychiatry. 2019 Aug 20;9:196. doi: 10.1038/s41398-019-0538-y (PMC6702224; doi:10.1038/s41398-019-0538-y)
Supplement: Supplementary file 2 — Supplementary figure and supplementary table. [file 41398_2019_538_MOESM2_ESM.pdf]

Supplementary Figure 1

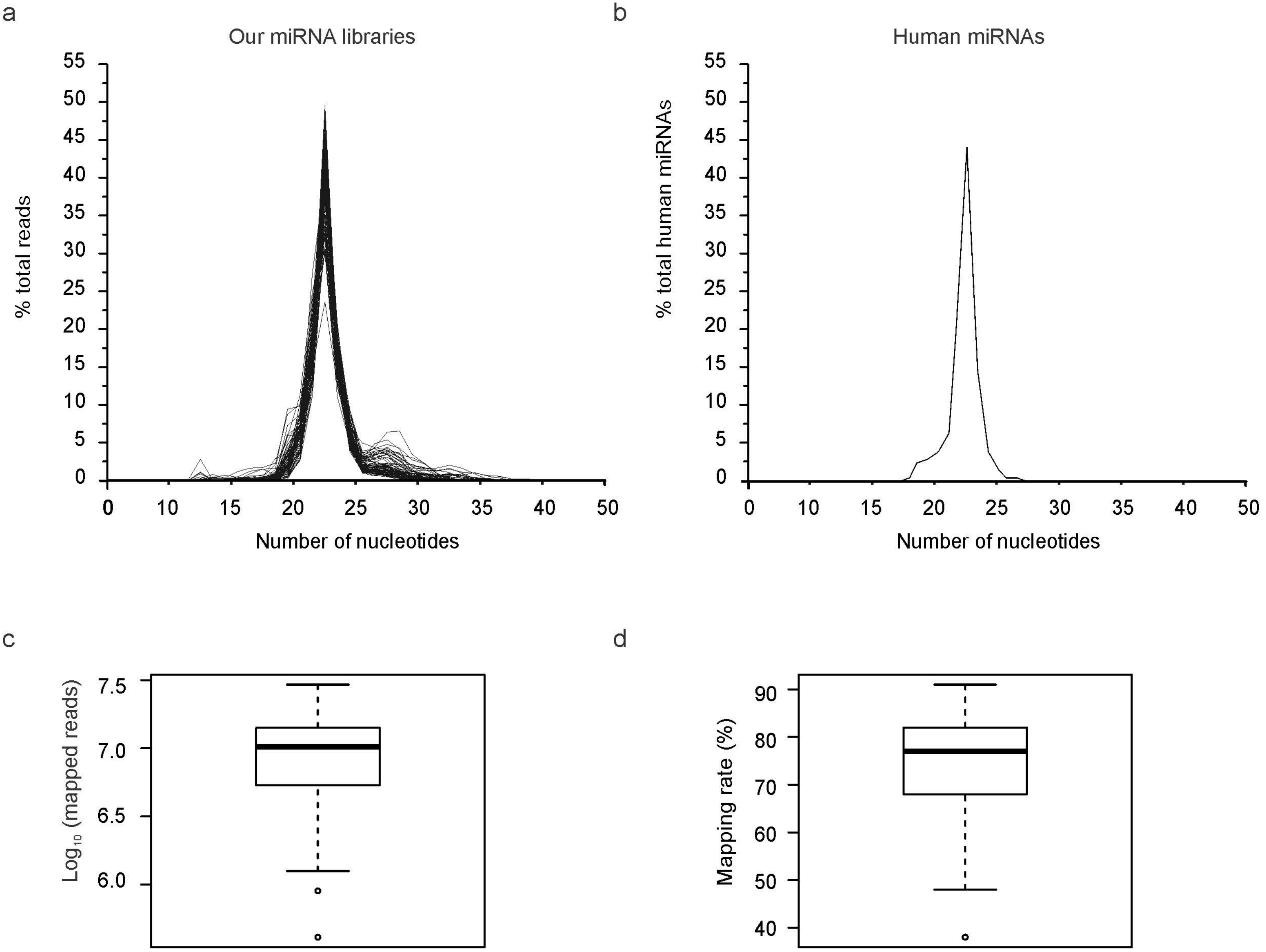

Supplementary Figure 2

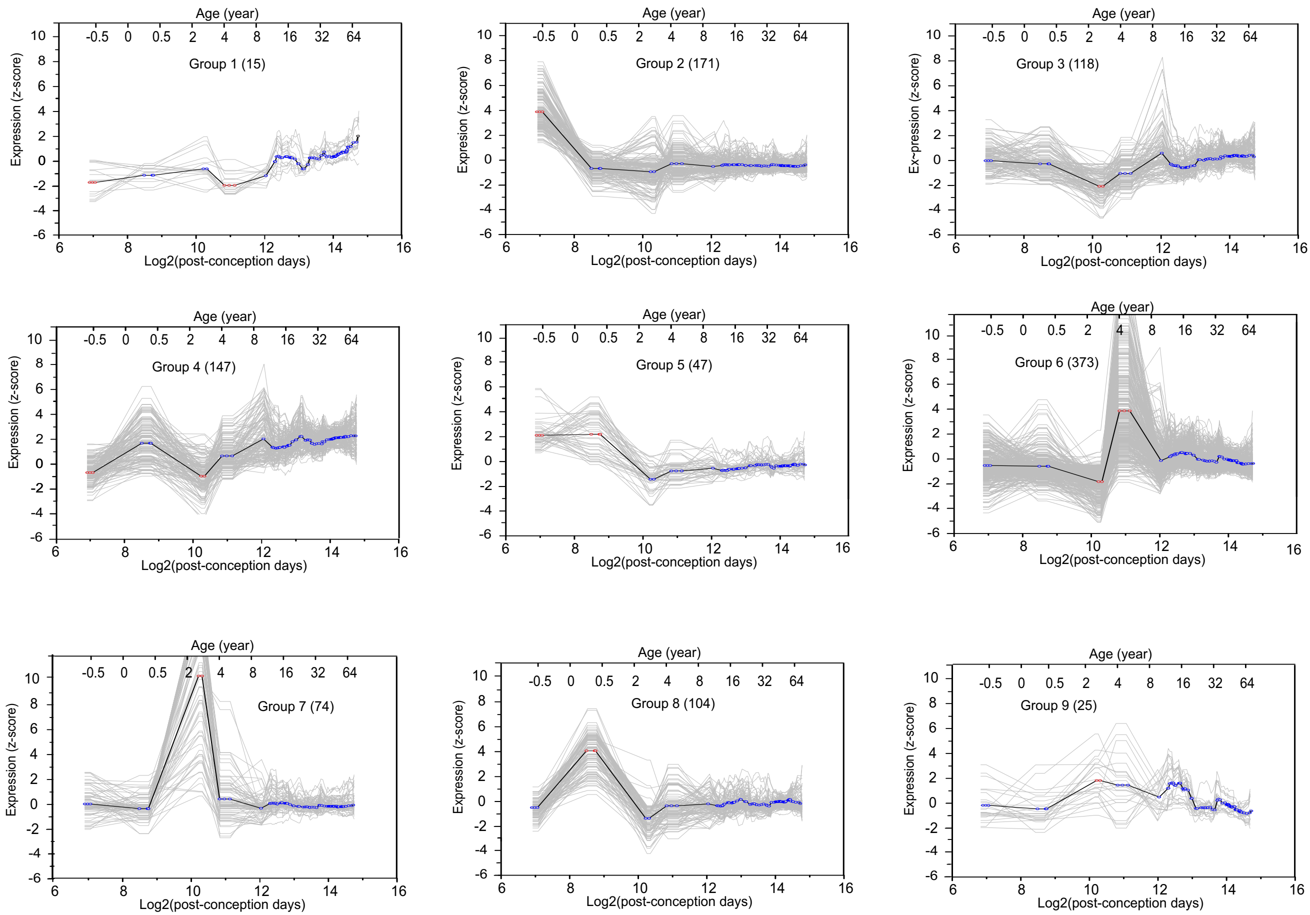

Supplementary Figure 3

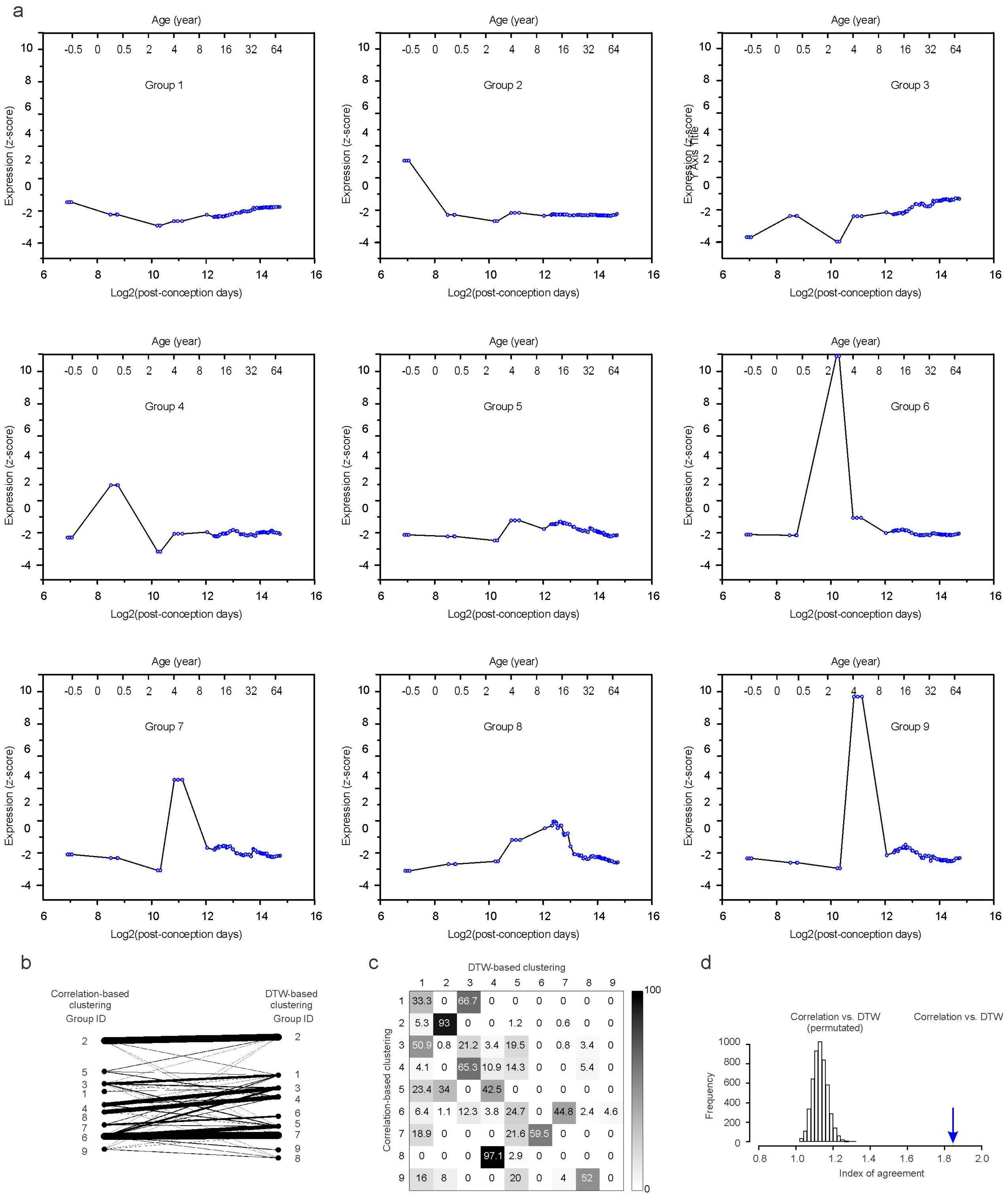

Supplementary Figure 4

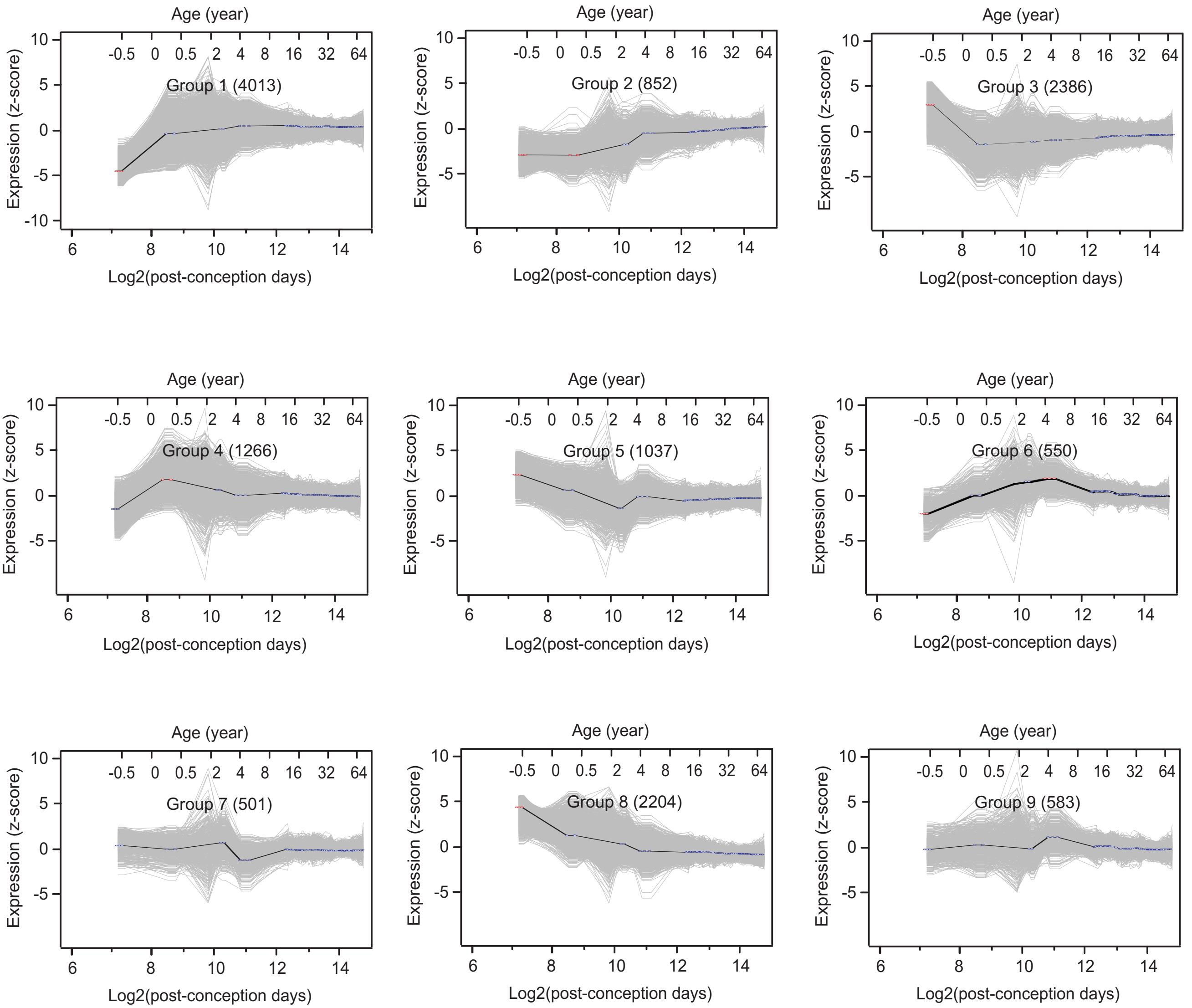

Supplementary Figure 5

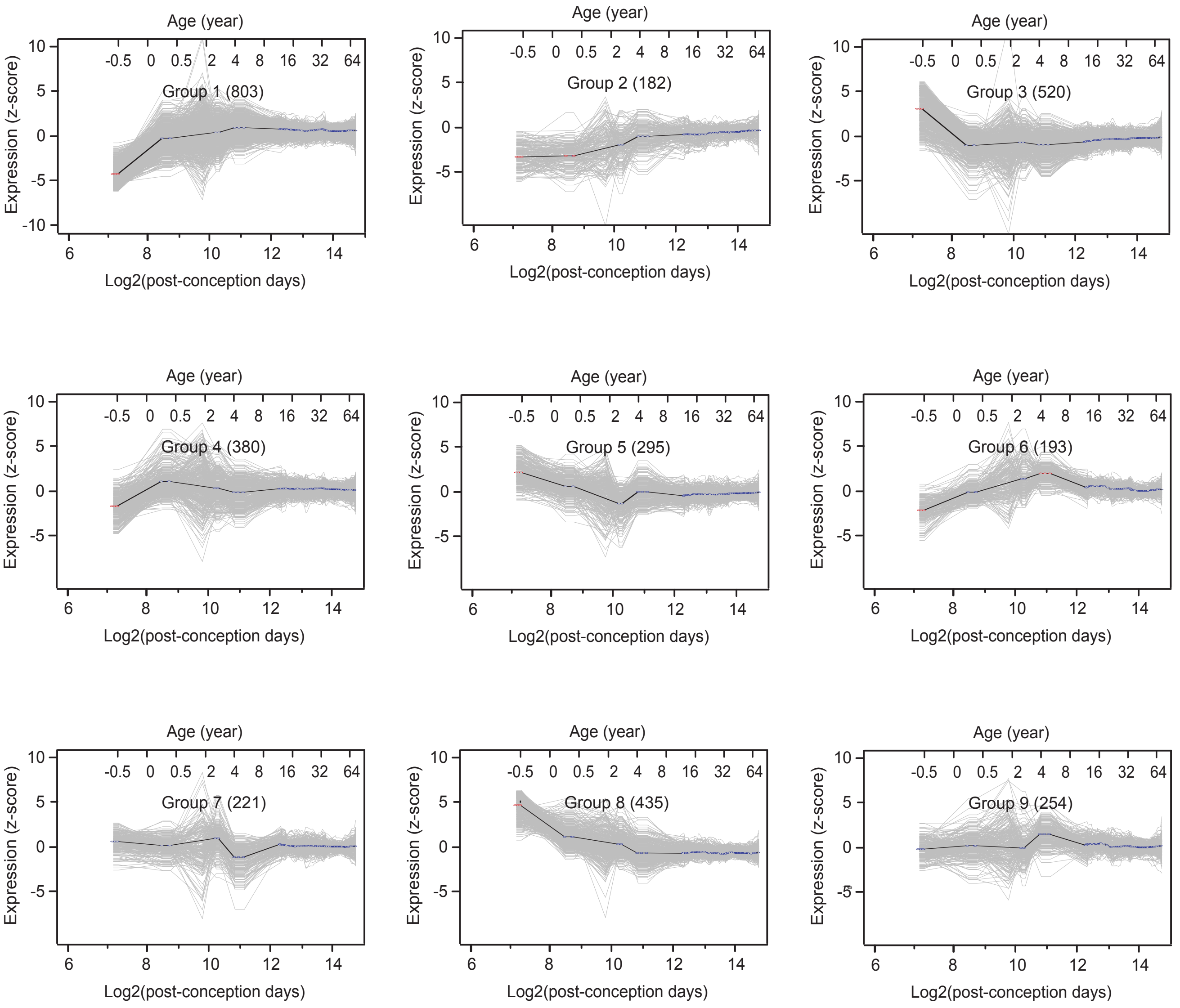

Supplementary Figure 6

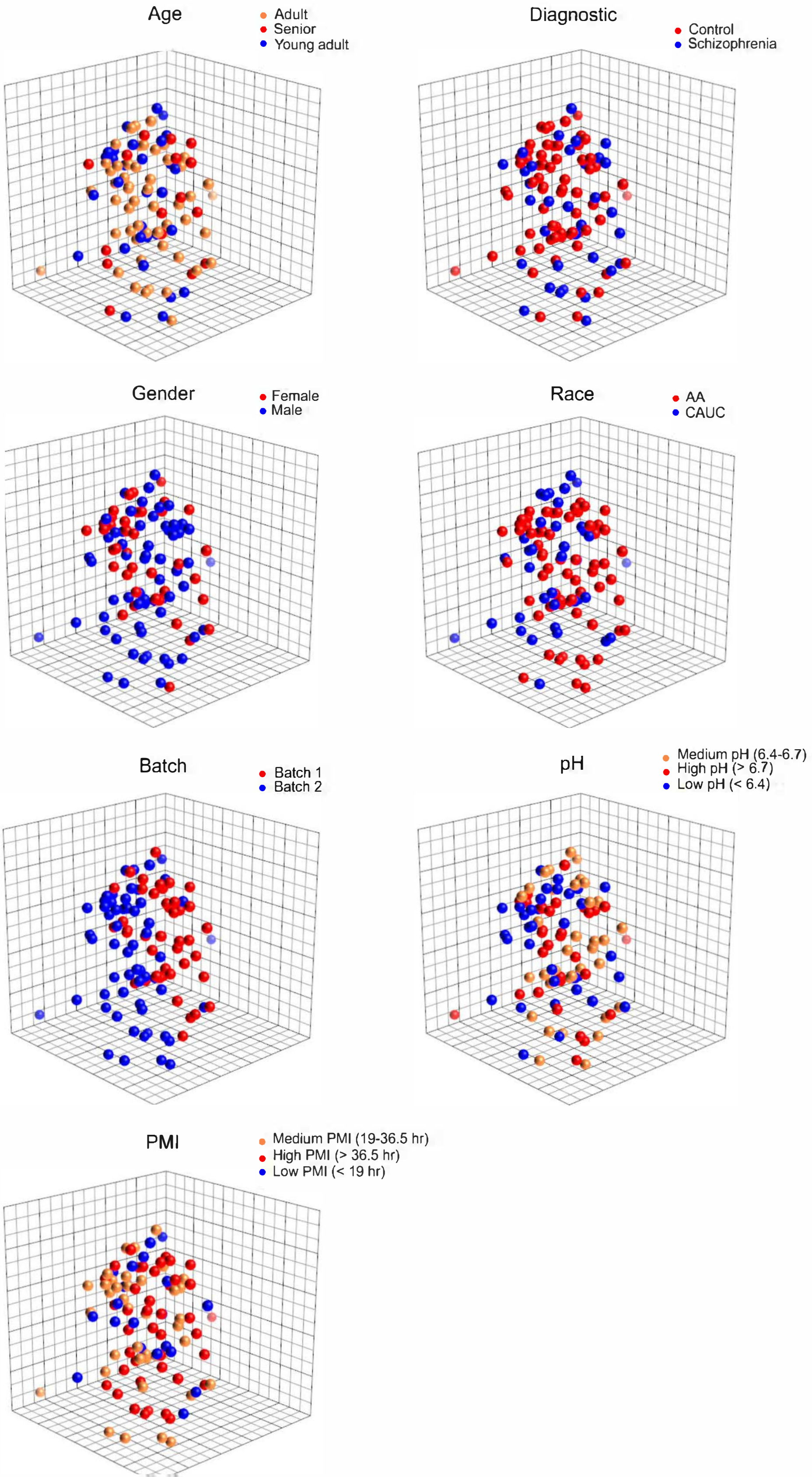

Supplementary Figure 7

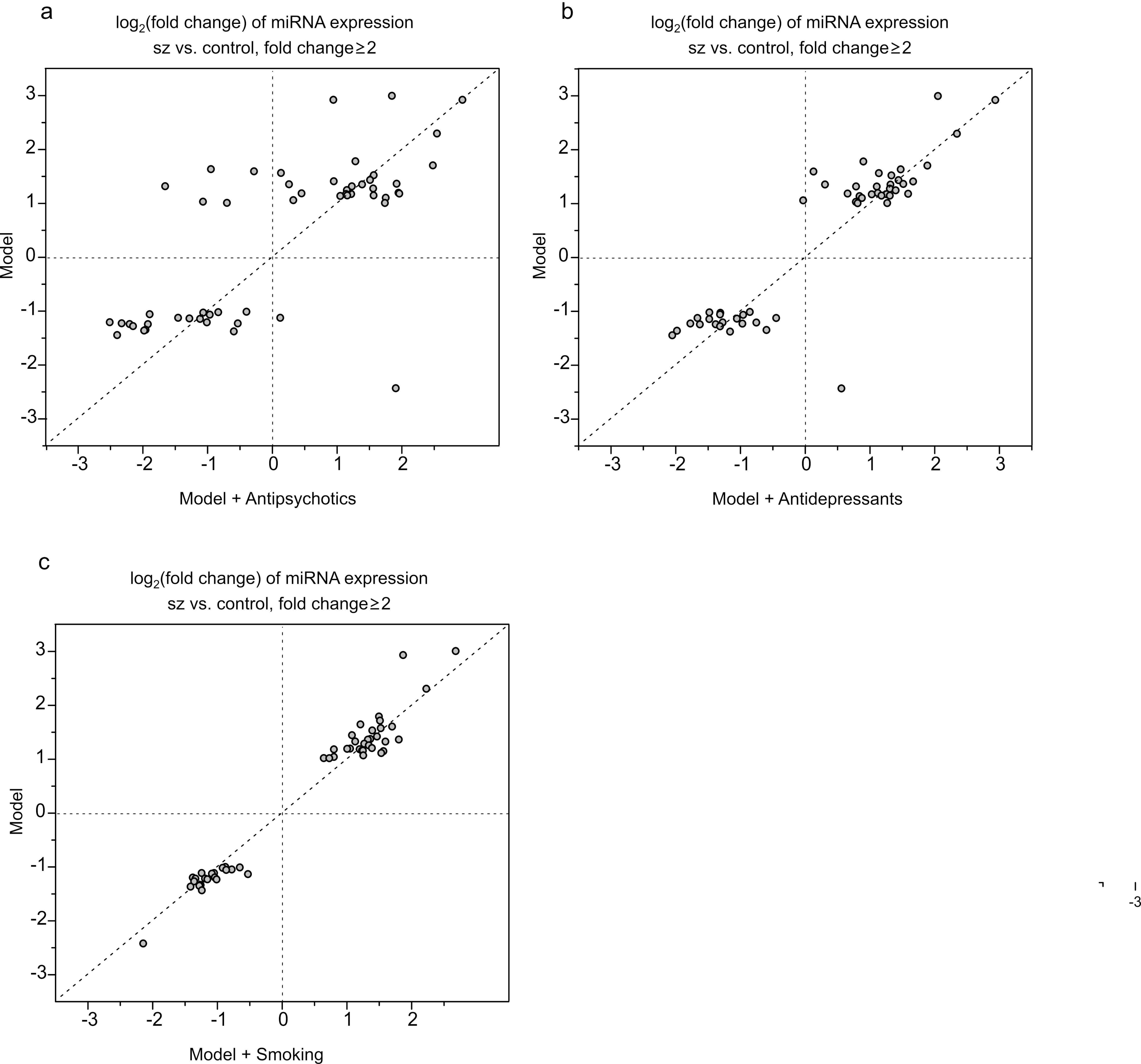

Supplementary Figure 8

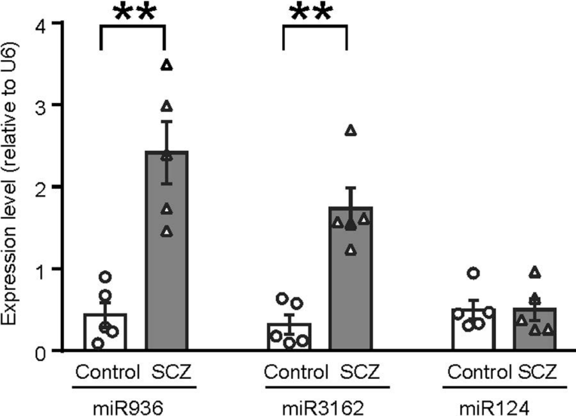

Supplementary Figure 9

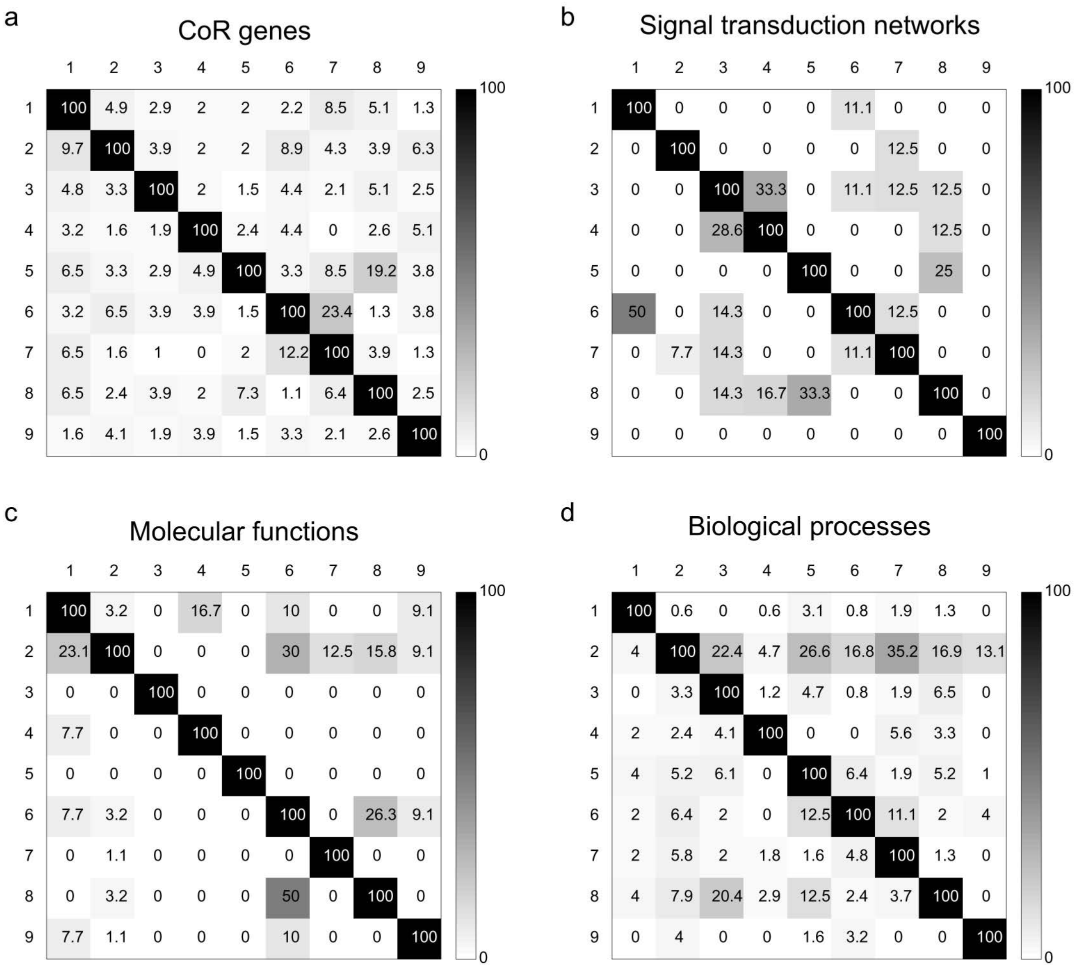

Supplementary Table 1. Demographic characteristics of brain donors used in the temporal profiling of miRNA expression

AA: African American; AS: Asian; CAUC: Caucasian

Brain ID	Age (year)	Gender	Race	Post-portem Interval (hour)	pH
1809	-0.44	F	AA	1	N/A
1790	-0.42	F	AA	1	N/A
1793	-0.42	F	AA	2	N/A
1780	-0.42	F	AA	3	N/A
1781	-0.42	M	AA	6	N/A
1782	-0.40	F	AA	1	N/A
1791	-0.40	F	AA	1	N/A
2047	-0.40	F	AA	2	6.09
1824	-0.40	F	AA	8	N/A
1826	-0.40	F	CAUC	3	N/A
1802	-0.40	M	AA	2	N/A
1789	-0.40	M	CAUC	1	N/A
1190	0.22	M	AA	61	6.48
847	0.39	F	AA	40.5	6.39
847	0.39	F	AA	40.5	6.39
1490	0.39	M	CAUC	29	6.33
957	0.41	M	AA	59.5	6.27
1091	0.41	M	AA	38	6.4
1380	1.6	F	CAUC	18	6.68
1872	2.5	F	CAUC	8	6.25
1866	2.7	F	CAUC	44	6.06
1218	4.2	M	AA	20	6.52
1855	4.7	M	CAUC	17	6.27
1870	5.3	M	CAUC	18	6.67
1975	10.7	F	AA	22	N/A
1553	13.0	F	CAUC	26	6.18
1670	13.1	F	CAUC	34.5	6.7
1435	13.5	F	AA	19.5	6.34
1966	14.0	M	CAUC	13	5.85
1848	14.1	M	CAUC	16	6.25
1878	14.6	M	CAUC	16	6.73
1847	14.6	M	CAUC	10	6.86
1875	15.1	F	CAUC	5	6.89
1412	15.2	F	CAUC	26	6.64
1964	15.2	M	AA	16	6.01
1876	16.4	M	CAUC	16	6.68
1856	16.7	F	CAUC	16	6.72
1881	16.8	M	CAUC	15	6.73
1859	16.8	M	CAUC	25	6.53

1292	17.8	M	AA	14	6.69
890	18.2	M	AA	36.5	6.7
1407	18.3	M	AA	14	6.37
1143	18.5	M	AA	11.5	6.62
1861	18.7	M	CAUC	9	6.12
1865	18.8	F	CAUC	15	6.67
885	19.9	M	AA	21.5	6.93
1563	21.3	F	AA	18	6.81
1306	21.5	M	AA	15	6.49
1558	23.4	F	CAUC	17.5	6.06
1518	24.0	M	CAUC	20.5	6.98
1340	26.2	F	AA	42	6.92
1604	27.2	M	AA	14	6.37
1204	27.5	M	CAUC	19.5	6.59
1389	28.4	M	CAUC	19	6.29
1469	28.6	M	CAUC	7.5	6.85
1410	29.8	M	AA	29.5	6.91
1556	30.0	F	AA	37.5	6.85
1761	30.0	M	CAUC	22	6.08
1405	31.5	F	AA	33.5	6.91
1142	33.0	M	AA	43	6.62
1446	34.5	M	CAUC	29.5	6.71
1191	36.6	M	AA	36.5	6.55
1408	37.2	M	AA	13	6.37
1324	38.6	F	AA	38.5	6.58
1409	40.6	F	CAUC	28.5	6.1
1674	42.8	M	CAUC	24.5	6.7
1105	44.2	F	AA	89.5	6.22
1473	44.5	M	AA	29	6.77
1517	44.8	F	AA	62.5	6.93
1644	45.3	M	AA	21.5	6.61
1179	45.4	F	AA	43	6.43
1432	45.7	M	CAUC	43.5	6.26
1325	45.8	F	AA	25	6.74
948	45.9	M	AA	38.5	6.66
1525	46.1	F	AA	13	7.1
1271	46.6	M	CAUC	16	6.93
1532	46.6	M	CAUC	68	6.59
1750	46.9	M	CAUC	26.5	6.16
1649	47.0	M	CAUC	67.5	6.84
1164	48.1	F	AA	16	6.62
1017	48.5	F	AA	23.5	6.25
1122	48.6	M	AA	23	6.67
1092	49.0	M	CAUC	18	6.13

1753	49.0	F	CAUC	36	6.47
1463	49.6	M	CAUC	18.5	6.57
1193	49.8	M	AA	51	6.83
1039	51.5	F	AA	24	6.79
1249	51.8	F	AA	28	6.13
1113	51.9	F	CAUC	51.5	6.53
859	52.2	M	AA	19.5	6.52
910	53.0	F	AA	10	6.66
1314	53.4	F	AA	50.5	6.5
946	54.0	M	AA	32.5	6.76
1351	54.0	M	CAUC	63	6.17
1539	54.6	F	AA	53.5	6.98
1007	57.1	M	AA	25	6.5
1034	57.6	M	CAUC	24	6.29
1006	57.9	M	AA	47.5	6.72
894	58.0	F	AA	24	6.06
1096	58.2	M	AS	37	6.27
1305	58.4	M	AA	50	5.96
1304	58.6	M	CAUC	64	6.87
1069	59.6	F	AA	37	6.64
1135	60.4	M	AA	44	6.59
1297	62.8	M	AA	37	6.79
1695	63.2	M	CAUC	26	6.74
925	66.7	F	CAUC	29.5	6.48
1756	71.1	F	CAUC	23.5	6.16
1160	73.3	M	AA	68.5	6.41

Supplementary Table 2. miRNA–mRNA target pairs correlated in expression

miRNA	mRNA gene ID	mRNA gene symbol	*r*	*p*- value	Adjusted *p* -value
hsa-miR-206	2908	NR3C1	-0.79	1.13E-21	1.90E-16
hsa-miR-206	64393	ZMAT3	-0.78	2.77E-21	4.69E-16
hsa-miR-206	5915	RARB	-0.77	2.51E-20	4.24E-15
hsa-miR-216b	55356	SLC22A15	-0.77	2.67E-20	4.52E-15
hsa-miR-206	4638	MYLK	-0.77	4.70E-20	7.95E-15
hsa-miR-206	4130	MAP1A	-0.76	1.89E-19	3.20E-14
hsa-miR-206	29953	TRHDE	-0.75	1.68E-18	2.84E-13
hsa-miR-206	10814	CPLX2	-0.74	2.81E-18	4.75E-13
hsa-miR-206	57446	NDRG3	-0.73	3.25E-17	5.49E-12
hsa-miR-206	30818	KCNIP3	-0.73	3.59E-17	6.07E-12
hsa-miR-206	4233	MET	-0.72	5.30E-17	8.97E-12
hsa-miR-216b	50484	RRM2B	-0.72	5.40E-17	9.14E-12
hsa-miR-206	5594	MAPK1	-0.72	6.62E-17	1.12E-11
hsa-miR-206	5218	CDK14	-0.72	1.20E-16	2.02E-11
hsa-miR-206	57496	MKL2	-0.72	1.42E-16	2.40E-11
hsa-miR-206	1390	CREM	-0.72	1.43E-16	2.42E-11
hsa-miR-216b	26038	CHD5	-0.72	1.63E-16	2.76E-11
hsa-miR-206	55742	PARVA	-0.71	2.38E-16	4.02E-11
hsa-miR-206	79161	TMEM243	-0.71	2.57E-16	4.35E-11
hsa-miR-206	6844	VAMP2	-0.71	3.00E-16	5.08E-11
hsa-miR-206	11076	TPPP	-0.71	3.01E-16	5.10E-11
hsa-miR-206	64398	MPP5	-0.71	3.59E-16	6.07E-11
hsa-miR-216b	3131	HLF	-0.71	5.79E-16	9.79E-11
hsa-miR-206	3727	JUND	-0.71	6.55E-16	1.11E-10
hsa-miR-206	64506	CPEB1	-0.70	1.26E-15	2.13E-10
hsa-miR-217	440279	UNC13C	-0.70	1.31E-15	2.22E-10
hsa-miR-206	4340	MOG	-0.70	1.62E-15	2.73E-10
hsa-miR-216b	5337	PLD1	-0.70	1.81E-15	3.06E-10
hsa-miR-216b	171425	CLYBL	-0.70	2.38E-15	4.03E-10
hsa-miR-206	4814	NINJ1	-0.70	2.46E-15	4.17E-10
hsa-miR-206	5595	MAPK3	-0.70	2.71E-15	4.58E-10
hsa-miR-216b	1408	CRY2	-0.70	2.73E-15	4.62E-10
hsa-miR-216b	6506	SLC1A2	-0.69	3.22E-15	5.44E-10
hsa-miR-206	23558	WBP2	-0.69	3.27E-15	5.53E-10
hsa-miR-216b	5027	P2RX7	-0.69	4.18E-15	7.07E-10
hsa-miR-206	54602	NDFIP2	-0.69	5.10E-15	8.63E-10
hsa-miR-206	143098	MPP7	-0.69	5.46E-15	9.23E-10
hsa-miR-206	10634	GAS2L1	-0.69	5.52E-15	9.33E-10
hsa-miR-206	2643	GCH1	-0.69	5.55E-15	9.39E-10
hsa-miR-206	91050	CCDC149	-0.69	5.64E-15	9.54E-10
hsa-miR-206	6238	RRBP1	-0.69	7.19E-15	1.22E-09
hsa-miR-206	266812	NAP1L5	-0.68	1.11E-14	1.87E-09
hsa-miR-206	307	ANXA4	-0.68	1.26E-14	2.13E-09
hsa-miR-206	523	ATP6V1A	-0.68	1.40E-14	2.37E-09

hsa-miR-216b	5813	PURA	-0.68	1.44E-14	2.43E-09
hsa-miR-206	140809	SRXN1	-0.68	2.66E-14	4.50E-09
hsa-miR-206	25994	HIGD1A	-0.68	2.85E-14	4.82E-09
hsa-miR-219-1-3p	1807	DPYS	-0.68	2.90E-14	4.90E-09
hsa-miR-206	5208	PFKFB2	-0.68	3.24E-14	5.47E-09
hsa-miR-206	6616	SNAP25	-0.67	3.48E-14	5.88E-09
hsa-miR-206	26580	BSCL2	-0.67	5.04E-14	8.53E-09
hsa-miR-206	55203	LGI2	-0.67	5.19E-14	8.77E-09
hsa-miR-217	5594	MAPK1	-0.67	5.27E-14	8.91E-09
hsa-miR-206	144699	FBXL14	-0.67	5.63E-14	9.53E-09
hsa-miR-206	388403	YPEL2	-0.67	5.83E-14	9.86E-09
hsa-miR-216b	54602	NDFIP2	-0.67	9.29E-14	1.57E-08
hsa-miR-216b	11176	BAZ2A	-0.67	9.53E-14	1.61E-08
hsa-miR-216b	7855	FZD5	-0.66	1.15E-13	1.94E-08
hsa-miR-217	10396	ATP8A1	-0.66	1.42E-13	2.40E-08
hsa-miR-206	348980	HCN1	-0.66	1.42E-13	2.41E-08
hsa-miR-206	10231	RCAN2	-0.66	1.89E-13	3.19E-08
hsa-miR-206	8525	DGKZ	-0.66	2.30E-13	3.89E-08
hsa-miR-206	10458	BAIAP2	-0.66	2.69E-13	4.55E-08
hsa-miR-206	377	ARF3	-0.66	3.02E-13	5.11E-08
hsa-miR-206	6095	RORA	-0.66	3.02E-13	5.11E-08
hsa-miR-206	6543	SLC8A2	-0.65	6.53E-13	1.10E-07
hsa-miR-216b	222223	KIAA1324L	-0.65	6.59E-13	1.11E-07
hsa-miR-216b	4038	LRP4	-0.65	9.06E-13	1.53E-07
hsa-miR-219-1-3p	222223	KIAA1324L	-0.65	9.10E-13	1.54E-07
hsa-miR-206	1213	CLTC	-0.65	9.13E-13	1.54E-07
hsa-miR-216b	64065	PERP	-0.65	9.95E-13	1.68E-07
hsa-miR-219-1-3p	23229	ARHGEF9	-0.64	1.02E-12	1.72E-07
hsa-miR-206	145567	TTC7B	-0.64	1.28E-12	2.16E-07
hsa-miR-296-3p	23092	ARHGAP26	-0.64	1.28E-12	2.17E-07
hsa-miR-216b	84333	PCGF5	-0.64	1.63E-12	2.75E-07
hsa-miR-217	6867	TACC1	-0.64	1.96E-12	3.32E-07
hsa-miR-206	9120	SLC16A6	-0.64	2.19E-12	3.71E-07
hsa-miR-216b	4088	SMAD3	-0.64	2.61E-12	4.41E-07
hsa-miR-216b	1831	TSC22D3	-0.64	2.82E-12	4.76E-07
hsa-miR-206	57659	ZBTB4	-0.63	2.95E-12	4.99E-07
hsa-miR-206	8927	BSN	-0.63	2.96E-12	5.01E-07
hsa-miR-216b	9882	TBC1D4	-0.63	3.95E-12	6.67E-07
hsa-miR-216b	5357	PLS1	-0.63	4.19E-12	7.08E-07
hsa-miR-217	8760	CDS2	-0.63	4.44E-12	7.51E-07
hsa-miR-206	147179	WIPF2	-0.63	6.02E-12	1.02E-06
hsa-miR-767-3p	116984	ARAP2	-0.63	6.12E-12	1.03E-06
hsa-miR-217	6400	SEL1L	-0.63	6.21E-12	1.05E-06
hsa-miR-301b	805	CALM2	-0.63	6.22E-12	1.05E-06
hsa-miR-301b	805	CALM3	-0.63	6.22E-12	1.05E-06
hsa-miR-301b	805	CALM1	-0.63	6.22E-12	1.05E-06
hsa-miR-767-3p	133522	PPARGC1B	-0.62	8.05E-12	1.36E-06
hsa-miR-301b	1960	EGR3	-0.62	8.06E-12	1.36E-06
hsa-miR-206	5581	PRKCE	-0.62	8.06E-12	1.36E-06

hsa-miR-301b	3131	HLF	-0.62	8.41E-12	1.42E-06
hsa-miR-206	55356	SLC22A15	-0.62	8.50E-12	1.44E-06
hsa-miR-219-1-3p	9722	NOS1AP	-0.62	8.77E-12	1.48E-06
hsa-miR-206	9568	GABBR2	-0.62	9.50E-12	1.61E-06
hsa-miR-296-3p	5354	PLP1	-0.62	1.00E-11	1.69E-06
hsa-miR-296-3p	55856	ACOT13	-0.62	1.10E-11	1.85E-06
hsa-miR-216b	4205	MEF2A	-0.62	1.14E-11	1.92E-06
hsa-miR-206	28964	GIT1	-0.62	1.22E-11	2.06E-06
hsa-miR-216b	25999	CLIP3	-0.62	1.27E-11	2.15E-06
hsa-miR-217	93664	CADPS2	-0.62	1.28E-11	2.17E-06
hsa-miR-206	1389	CREBL2	-0.62	1.49E-11	2.52E-06
hsa-miR-216a	2908	NR3C1	-0.62	1.90E-11	3.21E-06
hsa-miR-217	7447	VSNL1	-0.62	1.99E-11	3.37E-06
hsa-miR-767-3p	123041	SLC24A4	-0.61	2.17E-11	3.66E-06
hsa-miR-216b	4189	DNAJB9	-0.61	2.19E-11	3.70E-06
hsa-miR-216a	5915	RARB	-0.61	2.26E-11	3.82E-06
hsa-miR-206	57134	MAN1C1	-0.61	2.76E-11	4.67E-06
hsa-miR-206	79751	SLC25A22	-0.61	2.80E-11	4.73E-06
hsa-miR-206	10087	COL4A3BP	-0.61	2.88E-11	4.87E-06
hsa-miR-216a	133522	PPARGC1B	-0.61	3.17E-11	5.36E-06
hsa-miR-217	6854	SYN2	-0.61	3.39E-11	5.73E-06
hsa-miR-216b	6345	SRL	-0.61	3.67E-11	6.20E-06
hsa-miR-206	57514	ARHGAP31	-0.61	3.85E-11	6.51E-06
hsa-miR-206	26010	SPATS2L	-0.61	4.01E-11	6.77E-06
hsa-miR-767-5p	148641	SLC35F3	-0.61	4.02E-11	6.80E-06
hsa-miR-301b	11113	CIT	-0.61	4.75E-11	8.02E-06
hsa-miR-423-5p	6855	SYP	-0.60	5.75E-11	9.71E-06
hsa-miR-217	51660	MPC1	-0.60	6.40E-11	1.08E-05
hsa-miR-301b	3705	ITPK1	-0.60	6.60E-11	1.11E-05
hsa-miR-219-1-3p	8760	CDS2	-0.60	7.04E-11	1.19E-05
hsa-miR-767-3p	4130	MAP1A	-0.60	7.44E-11	1.26E-05
hsa-miR-296-3p	27351	DESI1	-0.60	7.46E-11	1.26E-05
hsa-miR-301b	57512	GPR158	-0.60	7.80E-11	1.32E-05
hsa-miR-206	79696	ZC2HC1C	-0.60	7.90E-11	1.33E-05
hsa-miR-296-3p	6376	CX3CL1	-0.60	8.75E-11	1.48E-05
hsa-miR-216a	5218	CDK14	-0.60	9.14E-11	1.54E-05
hsa-miR-217	4674	NAP1L2	-0.60	9.49E-11	1.60E-05
hsa-miR-216a	51701	NLK	-0.60	9.54E-11	1.61E-05
hsa-miR-206	84279	PRADC1	-0.60	9.69E-11	1.64E-05
hsa-miR-301b	51046	ST8SIA3	-0.60	9.73E-11	1.64E-05
hsa-miR-206	3759	KCNJ2	-0.60	9.81E-11	1.66E-05
hsa-miR-206	64759	TNS3	-0.60	1.15E-10	1.94E-05
hsa-miR-216a	81832	NETO1	-0.60	1.16E-10	1.95E-05
hsa-miR-217	56172	ANKH	-0.60	1.19E-10	2.01E-05
hsa-miR-301b	10955	SERINC3	-0.60	1.19E-10	2.01E-05
hsa-miR-423-5p	80023	NRSN2	-0.60	1.23E-10	2.08E-05
hsa-miR-423-5p	6560	SLC12A4	-0.60	1.25E-10	2.11E-05
hsa-miR-767-3p	490	ATP2B1	-0.60	1.29E-10	2.18E-05
hsa-miR-217	523	ATP6V1A	-0.59	1.35E-10	2.28E-05

hsa-miR-301b	9515	STXBP5L	-0.59	1.35E-10	2.28E-05
hsa-miR-216b	4303	FOXO4	-0.59	1.36E-10	2.29E-05
hsa-miR-423-5p	5886	RAD23A	-0.59	1.47E-10	2.47E-05
hsa-miR-301b	221002	RASGEF1A	-0.59	1.48E-10	2.51E-05
hsa-miR-301b	491	ATP2B2	-0.59	1.51E-10	2.54E-05
hsa-miR-301b	57468	SLC12A5	-0.59	1.52E-10	2.56E-05
hsa-miR-423-5p	6575	SLC20A2	-0.59	1.56E-10	2.64E-05
hsa-miR-206	55315	SLC29A3	-0.59	1.58E-10	2.66E-05
hsa-miR-216b	114134	SLC2A13	-0.59	1.58E-10	2.67E-05
hsa-miR-206	6717	SRI	-0.59	1.62E-10	2.73E-05
hsa-miR-219-1-3p	4185	ADAM11	-0.59	1.63E-10	2.75E-05
hsa-miR-206	9776	ATG13	-0.59	1.64E-10	2.76E-05
hsa-miR-423-5p	10814	CPLX2	-0.59	1.74E-10	2.93E-05
hsa-miR-301b	8437	RASAL1	-0.59	1.74E-10	2.94E-05
hsa-miR-767-3p	8646	CHRD	-0.59	1.75E-10	2.96E-05
hsa-miR-767-5p	9673	SLC25A44	-0.59	1.78E-10	3.00E-05
hsa-miR-216a	123041	SLC24A4	-0.59	1.78E-10	3.00E-05
hsa-miR-618	687	KLF9	-0.59	1.94E-10	3.27E-05
hsa-miR-217	1902	LPAR1	-0.59	2.07E-10	3.50E-05
hsa-miR-767-3p	132332	TMEM155	-0.59	2.22E-10	3.75E-05
hsa-miR-423-5p	7226	TRPM2	-0.59	2.51E-10	4.23E-05
hsa-miR-206	51361	HOOK1	-0.59	2.55E-10	4.30E-05
hsa-miR-217	9228	DLGAP2	-0.59	2.56E-10	4.31E-05
hsa-miR-125a-3p	55856	ACOT13	-0.59	2.78E-10	4.68E-05
hsa-miR-206	145376	PPP1R36	-0.59	2.82E-10	4.76E-05
hsa-miR-217	130733	TMEM178A	-0.59	3.11E-10	5.24E-05
hsa-miR-301b	130399	ACVR1C	-0.58	3.13E-10	5.29E-05
hsa-miR-301b	50649	ARHGEF4	-0.58	3.22E-10	5.43E-05
hsa-miR-423-5p	144404	TMEM120B	-0.58	3.22E-10	5.44E-05
hsa-miR-301b	6867	TACC1	-0.58	3.24E-10	5.46E-05
hsa-miR-216b	285440	CYP4V2	-0.58	3.27E-10	5.52E-05
hsa-miR-217	340554	ZC3H12B	-0.58	3.61E-10	6.09E-05
hsa-miR-206	57181	SLC39A10	-0.58	3.73E-10	6.29E-05
hsa-miR-206	8674	VAMP4	-0.58	3.86E-10	6.52E-05
hsa-miR-219-1-3p	56913	C1GALT1	-0.58	3.97E-10	6.70E-05
hsa-miR-484	55924	FAM212B	-0.58	4.02E-10	6.78E-05
hsa-miR-767-3p	3356	HTR2A	-0.58	4.17E-10	7.04E-05
hsa-miR-1275	28964	GIT1	-0.58	4.70E-10	7.93E-05
hsa-miR-767-5p	9802	DAZAP2	-0.58	4.82E-10	8.13E-05
hsa-miR-206	3140	MR1	-0.58	4.89E-10	8.25E-05
hsa-miR-301b	27020	NPTN	-0.58	5.37E-10	9.06E-05
hsa-miR-206	7049	TGFBR3	-0.58	5.79E-10	9.77E-05
hsa-miR-423-5p	1173	AP2M1	-0.58	5.81E-10	9.80E-05
hsa-miR-301b	116984	ARAP2	-0.58	6.10E-10	1.03E-04
hsa-miR-767-3p	3768	KCNJ12	-0.58	6.58E-10	1.11E-04
hsa-miR-423-5p	123041	SLC24A4	-0.58	6.99E-10	1.18E-04
hsa-miR-206	254170	FBXO33	-0.58	7.04E-10	1.19E-04
hsa-miR-423-5p	7425	VGF	-0.58	7.25E-10	1.22E-04
hsa-miR-206	79183	TTPAL	-0.57	7.58E-10	1.28E-04

hsa-miR-301b	11069	RAPGEF4	-0.57	7.74E-10	1.31E-04
hsa-miR-1275	2902	GRIN1	-0.57	7.94E-10	1.34E-04
hsa-miR-216a	2554	GABRA1	-0.57	8.13E-10	1.37E-04
hsa-miR-301b	10891	PPARGC1A	-0.57	8.32E-10	1.40E-04
hsa-miR-1276	25829	TMEM184B	-0.57	8.40E-10	1.42E-04
hsa-miR-301b	9053	MAP7	-0.57	8.44E-10	1.42E-04
hsa-miR-301b	120892	LRRK2	-0.57	8.60E-10	1.45E-04
hsa-miR-216b	159090	FAM122B	-0.57	8.69E-10	1.47E-04
hsa-miR-216b	80223	RAB11FIP1	-0.57	8.72E-10	1.47E-04
hsa-miR-301b	64061	TSPYL2	-0.57	9.28E-10	1.57E-04
hsa-miR-216a	23092	ARHGAP26	-0.57	9.54E-10	1.61E-04
hsa-miR-206	50862	RNF141	-0.57	9.76E-10	1.65E-04
hsa-miR-216a	146206	RLTPR	-0.57	9.81E-10	1.65E-04
hsa-miR-301b	133121	ENPP6	-0.57	9.82E-10	1.66E-04
hsa-miR-423-5p	57605	PITPNM2	-0.57	9.84E-10	1.66E-04
hsa-miR-423-5p	5476	CTSA	-0.57	1.00E-09	1.69E-04
hsa-miR-301b	64506	CPEB1	-0.57	1.01E-09	1.70E-04
hsa-miR-301b	4233	MET	-0.57	1.14E-09	1.93E-04
hsa-miR-206	50488	MINK1	-0.57	1.17E-09	1.97E-04
hsa-miR-301b	9615	GDA	-0.57	1.27E-09	2.15E-04
hsa-miR-206	11057	ABHD2	-0.57	1.37E-09	2.31E-04
hsa-miR-767-3p	6653	SORL1	-0.57	1.40E-09	2.35E-04
hsa-miR-1276	3749	KCNC4	-0.57	1.40E-09	2.37E-04
hsa-miR-125a-3p	5602	MAPK10	-0.57	1.52E-09	2.56E-04
hsa-miR-125a-3p	831	CAST	-0.57	1.52E-09	2.57E-04
hsa-miR-767-3p	783	CACNB2	-0.57	1.53E-09	2.59E-04
hsa-miR-301b	3766	KCNJ10	-0.57	1.58E-09	2.67E-04
hsa-miR-206	627	BDNF	-0.57	1.62E-09	2.74E-04
hsa-miR-330-3p	66000	TMEM108	-0.57	1.65E-09	2.78E-04
hsa-miR-216b	54502	RBM47	-0.56	1.65E-09	2.79E-04
hsa-miR-206	114134	SLC2A13	-0.56	1.71E-09	2.89E-04
hsa-miR-216b	1371	CPOX	-0.56	1.72E-09	2.90E-04
hsa-miR-217	11320	MGAT4A	-0.56	1.76E-09	2.97E-04
hsa-miR-767-5p	51701	NLK	-0.56	1.78E-09	3.00E-04
hsa-miR-296-3p	5578	PRKCA	-0.56	1.85E-09	3.12E-04
hsa-miR-206	2533	FYB	-0.56	1.95E-09	3.28E-04
hsa-miR-125a-3p	56894	AGPAT3	-0.56	2.09E-09	3.52E-04
hsa-miR-483-5p	57468	SLC12A5	-0.56	2.09E-09	3.53E-04
hsa-miR-217	6804	STX1A	-0.56	2.16E-09	3.65E-04
hsa-miR-1276	54414	SIAE	-0.56	2.32E-09	3.91E-04
hsa-miR-423-5p	9751	SNPH	-0.56	2.37E-09	4.00E-04
hsa-miR-206	80777	CYB5B	-0.56	2.50E-09	4.22E-04
hsa-miR-767-5p	84940	CORO6	-0.56	2.74E-09	4.62E-04
hsa-miR-767-3p	5909	RAP1GAP	-0.56	2.81E-09	4.73E-04
hsa-miR-301b	57605	PITPNM2	-0.56	2.89E-09	4.87E-04
hsa-miR-301b	1200	TPP1	-0.56	2.95E-09	4.98E-04
hsa-miR-767-5p	493	ATP2B4	-0.56	3.15E-09	5.31E-04
hsa-miR-216a	5581	PRKCE	-0.56	3.20E-09	5.40E-04
hsa-miR-219-1-3p	10564	ARFGEF2	-0.56	3.23E-09	5.44E-04

hsa-miR-217	10533	ATG7	-0.56	3.24E-09	5.47E-04
hsa-miR-217	6506	SLC1A2	-0.56	3.32E-09	5.60E-04
hsa-miR-423-5p	2077	ERF	-0.56	3.44E-09	5.79E-04
hsa-miR-206	90809	TMEM55B	-0.56	3.45E-09	5.82E-04
hsa-miR-301b	22903	BTBD3	-0.56	3.48E-09	5.86E-04
hsa-miR-767-3p	399665	FAM102A	-0.56	3.59E-09	6.06E-04
hsa-miR-767-3p	57185	NIPAL3	-0.56	3.62E-09	6.10E-04
hsa-miR-767-3p	9145	SYNGR1	-0.55	3.82E-09	6.44E-04
hsa-miR-301b	6616	SNAP25	-0.55	3.88E-09	6.54E-04
hsa-miR-216a	2107	ETF1	-0.55	3.94E-09	6.64E-04
hsa-miR-301b	64393	ZMAT3	-0.55	4.09E-09	6.90E-04
hsa-miR-618	9806	SPOCK2	-0.55	4.24E-09	7.14E-04
hsa-miR-423-5p	91608	RASL10B	-0.55	4.24E-09	7.15E-04
hsa-miR-301b	492	ATP2B3	-0.55	4.57E-09	7.70E-04
hsa-miR-301b	3251	HPRT1	-0.55	4.71E-09	7.93E-04
hsa-miR-330-3p	1525	CXADR	-0.55	4.93E-09	8.31E-04
hsa-miR-301b	9825	SPATA2	-0.55	4.96E-09	8.36E-04
hsa-miR-216b	55071	C9orf40	-0.55	5.11E-09	8.60E-04
hsa-miR-885-5p	114818	KLHL29	-0.55	5.25E-09	8.85E-04
hsa-miR-217	282996	RBM20	-0.55	5.27E-09	8.88E-04
hsa-miR-217	1958	EGR1	-0.55	5.27E-09	8.89E-04
hsa-miR-301b	29906	ST8SIA5	-0.55	5.42E-09	9.13E-04
hsa-miR-767-3p	9854	C2CD2L	-0.55	5.55E-09	9.36E-04
hsa-miR-767-5p	63908	NAPB	-0.55	5.75E-09	9.68E-04
hsa-miR-206	9530	BAG4	-0.55	6.08E-09	1.02E-03
hsa-miR-216a	57732	ZFYVE28	-0.55	6.09E-09	1.03E-03
hsa-miR-301b	23167	EFR3A	-0.55	6.35E-09	1.07E-03
hsa-miR-301b	9917	FAM20B	-0.55	6.36E-09	1.07E-03
hsa-miR-216b	1286	COL4A4	-0.55	6.45E-09	1.09E-03
hsa-miR-767-3p	55246	CCDC25	-0.55	6.53E-09	1.10E-03
hsa-miR-216a	79789	CLMN	-0.55	6.60E-09	1.11E-03
hsa-miR-618	29993	PACSIN1	-0.55	6.63E-09	1.12E-03
hsa-miR-216a	523	ATP6V1A	-0.55	6.89E-09	1.16E-03
hsa-miR-217	205564	SENP5	-0.55	6.91E-09	1.16E-03
hsa-miR-216a	2246	FGF1	-0.55	6.91E-09	1.16E-03
hsa-miR-219-1-3p	147179	WIPF2	-0.55	7.51E-09	1.27E-03
hsa-miR-301b	5611	DNAJC3	-0.55	7.71E-09	1.30E-03
hsa-miR-216a	4209	MEF2D	-0.55	7.73E-09	1.30E-03
hsa-miR-125a-3p	5027	P2RX7	-0.55	7.78E-09	1.31E-03
hsa-miR-301b	526	ATP6V1B2	-0.54	7.88E-09	1.33E-03
hsa-miR-301b	53344	CHIC1	-0.54	8.08E-09	1.36E-03
hsa-miR-1276	5611	DNAJC3	-0.54	8.09E-09	1.36E-03
hsa-miR-296-3p	11226	GALNT6	-0.54	8.44E-09	1.42E-03
hsa-miR-206	5784	PTPN14	-0.54	8.63E-09	1.45E-03
hsa-miR-217	85465	EPT1	-0.54	8.72E-09	1.47E-03
hsa-miR-330-3p	4330	MN1	-0.54	9.17E-09	1.55E-03
hsa-miR-423-5p	374659	HDDC3	-0.54	9.57E-09	1.61E-03
hsa-miR-216a	171425	CLYBL	-0.54	9.73E-09	1.64E-03
hsa-miR-767-5p	64718	UNKL	-0.54	9.91E-09	1.67E-03

hsa-miR-338-5p	7750	ZMYM2	-0.54	1.02E-08	1.72E-03
hsa-miR-125a-3p	5999	RGS4	-0.54	1.05E-08	1.76E-03
hsa-miR-767-5p	266812	NAP1L5	-0.54	1.08E-08	1.82E-03
hsa-miR-301b	1540	CYLD	-0.54	1.15E-08	1.93E-03
hsa-miR-301b	4306	NR3C2	-0.54	1.15E-08	1.93E-03
hsa-miR-1275	6844	VAMP2	-0.54	1.16E-08	1.95E-03
hsa-miR-301b	84159	ARID5B	-0.54	1.16E-08	1.96E-03
hsa-miR-423-5p	7008	TEF	-0.54	1.20E-08	2.02E-03
hsa-miR-125a-3p	2785	GNG3	-0.54	1.24E-08	2.09E-03
hsa-miR-618	4129	MAOB	-0.54	1.24E-08	2.10E-03
hsa-miR-125a-3p	91522	COL23A1	-0.54	1.30E-08	2.20E-03
hsa-miR-301b	90627	STARD13	-0.54	1.31E-08	2.21E-03
hsa-miR-767-5p	7456	WIPF1	-0.54	1.33E-08	2.24E-03
hsa-miR-301b	140735	DYNLL2	-0.54	1.36E-08	2.30E-03
hsa-miR-423-5p	79178	THTPA	-0.54	1.41E-08	2.37E-03
hsa-miR-206	57608	KIAA1462	-0.54	1.50E-08	2.52E-03
hsa-miR-767-3p	3423	IDS	-0.54	1.58E-08	2.66E-03
hsa-miR-301b	11138	TBC1D8	-0.54	1.61E-08	2.70E-03
hsa-miR-301b	8534	CHST1	-0.53	1.70E-08	2.87E-03
hsa-miR-217	4008	LMO7	-0.53	1.73E-08	2.91E-03
hsa-miR-1276	55203	LGI2	-0.53	1.76E-08	2.97E-03
hsa-miR-3126-5p	2821	GPI	-0.53	1.93E-08	3.25E-03
hsa-miR-217	27133	KCNH5	-0.53	1.95E-08	3.28E-03
hsa-miR-1275	1838	DTNB	-0.53	2.04E-08	3.43E-03
hsa-miR-206	114789	SLC25A25	-0.53	2.04E-08	3.43E-03
hsa-miR-423-5p	2011	MARK2	-0.53	2.05E-08	3.46E-03
hsa-miR-217	353116	RILPL1	-0.53	2.06E-08	3.46E-03
hsa-miR-767-3p	4128	MAOA	-0.53	2.07E-08	3.48E-03
hsa-miR-767-3p	6272	SORT1	-0.53	2.07E-08	3.49E-03
hsa-miR-217	9699	RIMS2	-0.53	2.30E-08	3.87E-03
hsa-miR-217	80331	DNAJC5	-0.53	2.30E-08	3.88E-03
hsa-miR-301b	152789	JAKMIP1	-0.53	2.33E-08	3.92E-03
hsa-miR-767-3p	488	ATP2A2	-0.53	2.34E-08	3.94E-03
hsa-miR-767-5p	2060	EPS15	-0.53	2.35E-08	3.96E-03
hsa-miR-301b	6875	TAF4B	-0.53	2.37E-08	3.99E-03
hsa-miR-767-5p	23507	LRRC8B	-0.53	2.40E-08	4.03E-03
hsa-miR-301b	146691	TOM1L2	-0.53	2.41E-08	4.05E-03
hsa-miR-1276	5913	RAPSN	-0.53	2.66E-08	4.48E-03
hsa-miR-767-3p	5525	PPP2R5A	-0.53	2.75E-08	4.64E-03
hsa-miR-767-3p	56135	PCDHAC1	-0.53	2.78E-08	4.68E-03
hsa-miR-767-5p	5354	PLP1	-0.53	2.87E-08	4.83E-03
hsa-miR-618	5165	PDK3	-0.53	2.89E-08	4.86E-03
hsa-miR-125a-3p	9066	SYT7	-0.53	2.89E-08	4.86E-03
hsa-miR-206	7078	TIMP3	-0.53	2.97E-08	5.00E-03
hsa-miR-216a	54664	TMEM106B	-0.53	3.11E-08	5.24E-03
hsa-miR-301b	22875	ENPP4	-0.53	3.13E-08	5.28E-03
hsa-miR-330-3p	89796	NAV1	-0.53	3.29E-08	5.53E-03
hsa-miR-206	2697	GJA1	-0.53	3.34E-08	5.62E-03
hsa-miR-217	9201	DCLK1	-0.52	3.39E-08	5.71E-03

hsa-miR-618	27115	PDE7B	-0.52	3.45E-08	5.80E-03
hsa-miR-216a	26249	KLHL3	-0.52	3.48E-08	5.85E-03
hsa-miR-423-5p	25946	ZNF385A	-0.52	3.53E-08	5.94E-03
hsa-miR-338-5p	57565	KLHL14	-0.52	3.55E-08	5.98E-03
hsa-miR-330-3p	57616	TSHZ3	-0.52	3.65E-08	6.15E-03
hsa-miR-767-5p	54566	EPB41L4B	-0.52	3.67E-08	6.17E-03
hsa-miR-1275	63876	PKNOX2	-0.52	3.71E-08	6.25E-03
hsa-miR-301b	64094	SMOC2	-0.52	3.73E-08	6.27E-03
hsa-miR-1276	4130	MAP1A	-0.52	3.83E-08	6.44E-03
hsa-miR-301b	374986	FAM73A	-0.52	3.93E-08	6.61E-03
hsa-miR-206	56848	SPHK2	-0.52	3.98E-08	6.71E-03
hsa-miR-767-3p	2902	GRIN1	-0.52	4.14E-08	6.97E-03
hsa-miR-423-5p	154790	CLEC2L	-0.52	4.20E-08	7.07E-03
hsa-miR-301b	9648	GCC2	-0.52	4.27E-08	7.18E-03
hsa-miR-618	4209	MEF2D	-0.52	4.34E-08	7.31E-03
hsa-miR-301b	22853	LMTK2	-0.52	4.46E-08	7.50E-03
hsa-miR-125a-3p	51701	NLK	-0.52	4.74E-08	7.98E-03
hsa-miR-3659	114815	SORCS1	-0.52	4.79E-08	8.06E-03
hsa-miR-206	9201	DCLK1	-0.52	4.93E-08	8.30E-03
hsa-miR-1276	10768	AHCYL1	-0.52	4.94E-08	8.31E-03
hsa-miR-618	5813	PURA	-0.52	4.94E-08	8.31E-03
hsa-miR-330-3p	79570	NKAIN1	-0.52	4.94E-08	8.32E-03
hsa-miR-484	4209	MEF2D	-0.52	5.01E-08	8.43E-03
hsa-miR-767-3p	282996	RBM20	-0.52	5.04E-08	8.48E-03
hsa-miR-217	6529	SLC6A1	-0.52	5.15E-08	8.66E-03
hsa-miR-618	9249	DHRS3	-0.52	5.15E-08	8.67E-03
hsa-miR-423-5p	23125	CAMTA2	-0.52	5.32E-08	8.95E-03
hsa-miR-330-3p	6664	SOX11	-0.52	5.34E-08	8.99E-03
hsa-miR-301b	9751	SNPH	-0.52	5.46E-08	9.19E-03
hsa-miR-301b	10040	TOM1L1	-0.52	5.47E-08	9.21E-03
hsa-miR-217	81565	NDEL1	-0.52	5.50E-08	9.25E-03
hsa-miR-301b	5594	MAPK1	-0.52	5.50E-08	9.26E-03
hsa-miR-423-5p	160	AP2A1	-0.52	5.61E-08	9.44E-03
hsa-miR-301b	51555	PEX5L	-0.52	5.69E-08	9.58E-03
hsa-miR-217	7905	REEP5	-0.52	5.75E-08	9.68E-03
hsa-miR-618	23258	DENND5A	-0.52	5.77E-08	9.72E-03
hsa-miR-217	10085	EDIL3	-0.52	5.87E-08	9.88E-03
hsa-miR-610	169026	SLC30A8	-0.52	5.89E-08	9.91E-03
hsa-miR-219-2-3p	84620	ST6GAL2	-0.52	6.18E-08	1.04E-02
hsa-miR-301b	22870	PPP6R1	-0.52	6.44E-08	1.08E-02
hsa-miR-1276	956	ENTPD3	-0.52	6.47E-08	1.09E-02
hsa-miR-217	84302	TMEM246	-0.52	6.71E-08	1.13E-02
hsa-miR-206	658	BMPR1B	-0.52	6.77E-08	1.14E-02
hsa-miR-330-3p	57182	ANKRD50	-0.51	6.84E-08	1.15E-02
hsa-miR-1270	26999	CYFIP2	-0.51	6.85E-08	1.15E-02
hsa-miR-1270	26999	CYFIP2	-0.51	6.85E-08	1.15E-02
hsa-miR-1275	8153	RND2	-0.51	7.02E-08	1.18E-02
hsa-miR-1275	9254	CACNA2D2	-0.51	7.11E-08	1.20E-02
hsa-miR-767-5p	55799	CACNA2D3	-0.51	7.20E-08	1.21E-02

hsa-miR-301b	7039	TGFA	-0.51	7.24E-08	1.22E-02
hsa-miR-618	10458	BAIAP2	-0.51	7.38E-08	1.24E-02
hsa-miR-301b	3739	KCNA4	-0.51	7.45E-08	1.25E-02
hsa-miR-217	481	ATP1B1	-0.51	7.72E-08	1.30E-02
hsa-miR-206	51495	PTPLAD1	-0.51	7.82E-08	1.31E-02
hsa-miR-885-5p	22978	NT5C2	-0.51	7.83E-08	1.32E-02
hsa-miR-3179	8672	EIF4G3	-0.51	7.88E-08	1.32E-02
hsa-miR-3179	8672	EIF4G3	-0.51	7.88E-08	1.32E-02
hsa-miR-3179	8672	EIF4G3	-0.51	7.88E-08	1.32E-02
hsa-miR-885-5p	80312	TET1	-0.51	8.23E-08	1.38E-02
hsa-miR-206	8526	DGKE	-0.51	8.46E-08	1.42E-02
hsa-miR-301b	26038	CHD5	-0.51	8.59E-08	1.44E-02
hsa-miR-206	1121	CHM	-0.51	8.61E-08	1.45E-02
hsa-miR-301b	4154	MBNL1	-0.51	8.86E-08	1.49E-02
hsa-miR-301b	23336	SYNM	-0.51	9.01E-08	1.52E-02
hsa-miR-767-3p	4779	NFE2L1	-0.51	9.05E-08	1.52E-02
hsa-miR-330-3p	7468	WHSC1	-0.51	9.05E-08	1.52E-02
hsa-miR-3179	6844	VAMP2	-0.51	9.51E-08	1.60E-02
hsa-miR-3179	6844	VAMP2	-0.51	9.51E-08	1.60E-02
hsa-miR-3179	6844	VAMP2	-0.51	9.51E-08	1.60E-02
hsa-miR-301b	10618	TGOLN2	-0.51	9.53E-08	1.60E-02
hsa-miR-206	2036	EPB41L1	-0.51	1.03E-07	1.73E-02
hsa-miR-654-5p	51673	TPPP3	-0.51	1.04E-07	1.75E-02
hsa-miR-3179	133522	PPARGC1B	-0.51	1.05E-07	1.76E-02
hsa-miR-3179	133522	PPARGC1B	-0.51	1.05E-07	1.76E-02
hsa-miR-3179	133522	PPARGC1B	-0.51	1.05E-07	1.76E-02
hsa-miR-330-3p	55752	11-Sep	-0.51	1.05E-07	1.77E-02
hsa-miR-206	11320	MGAT4A	-0.51	1.06E-07	1.79E-02
hsa-miR-206	6546	SLC8A1	-0.51	1.13E-07	1.90E-02
hsa-miR-1276	55349	CHDH	-0.51	1.13E-07	1.90E-02
hsa-miR-301b	2065	ERBB3	-0.51	1.13E-07	1.91E-02
hsa-miR-125a-3p	6612	SUMO3	-0.51	1.14E-07	1.91E-02
hsa-miR-1270	192683	SCAMP5	-0.51	1.15E-07	1.93E-02
hsa-miR-1270	192683	SCAMP5	-0.51	1.15E-07	1.93E-02
hsa-miR-217	5110	PCMT1	-0.51	1.17E-07	1.96E-02
hsa-miR-338-5p	57554	LRRC7	-0.51	1.17E-07	1.98E-02
hsa-miR-618	2557	GABRA4	-0.51	1.18E-07	1.98E-02
hsa-miR-301b	23052	ENDOD1	-0.51	1.19E-07	2.00E-02
hsa-miR-1276	3768	KCNJ12	-0.51	1.19E-07	2.01E-02
hsa-miR-206	1742	DLG4	-0.51	1.23E-07	2.07E-02
hsa-miR-1276	27347	STK39	-0.51	1.24E-07	2.08E-02
hsa-miR-125a-3p	4515	MTCP1	-0.51	1.25E-07	2.10E-02
hsa-miR-125a-3p	2011	MARK2	-0.51	1.29E-07	2.17E-02
hsa-miR-330-5p	81619	TSPAN14	-0.51	1.29E-07	2.17E-02
hsa-miR-219-1-3p	25946	ZNF385A	-0.50	1.33E-07	2.24E-02
hsa-miR-125a-3p	2734	GLG1	-0.50	1.34E-07	2.25E-02
hsa-miR-216a	10215	OLIG2	-0.50	1.36E-07	2.28E-02
hsa-miR-1276	121256	TMEM132D	-0.50	1.36E-07	2.29E-02
hsa-miR-885-5p	54934	KANSL2	-0.50	1.37E-07	2.30E-02

hsa-miR-206	221938	MMD2	-0.50	1.37E-07	2.30E-02
hsa-miR-206	9895	TECPR2	-0.50	1.37E-07	2.31E-02
hsa-miR-301b	54557	SGTB	-0.50	1.39E-07	2.34E-02
hsa-miR-206	2354	FOSB	-0.50	1.41E-07	2.38E-02
hsa-miR-330-5p	6624	FSCN1	-0.50	1.42E-07	2.39E-02
hsa-miR-301b	29982	NRBF2	-0.50	1.43E-07	2.41E-02
hsa-miR-330-3p	2059	EPS8	-0.50	1.44E-07	2.42E-02
hsa-miR-125a-3p	8563	THOC5	-0.50	1.47E-07	2.47E-02
hsa-miR-301b	1040	CDS1	-0.50	1.51E-07	2.54E-02
hsa-miR-301b	10928	RALBP1	-0.50	1.57E-07	2.64E-02
hsa-miR-1276	4836	NMT1	-0.50	1.60E-07	2.70E-02
hsa-miR-767-5p	3632	INPP5A	-0.50	1.63E-07	2.74E-02
hsa-miR-301b	85461	TANC1	-0.50	1.66E-07	2.78E-02
hsa-miR-338-5p	92	ACVR2A	-0.50	1.72E-07	2.90E-02
hsa-miR-296-3p	389072	PLEKHM3	-0.50	1.79E-07	3.01E-02
hsa-miR-3164	7068	THRB	-0.50	1.82E-07	3.06E-02
hsa-miR-330-3p	7976	FZD3	-0.50	1.84E-07	3.08E-02
hsa-miR-1275	9628	RGS6	-0.50	1.87E-07	3.14E-02
hsa-miR-301b	4675	NAP1L3	-0.50	1.89E-07	3.18E-02
hsa-miR-125a-3p	22930	RAB3GAP1	-0.50	1.89E-07	3.18E-02
hsa-miR-206	130733	TMEM178A	-0.50	1.92E-07	3.22E-02
hsa-miR-423-5p	9254	CACNA2D2	-0.50	1.96E-07	3.29E-02
hsa-miR-217	54765	TRIM44	-0.50	2.13E-07	3.58E-02
hsa-miR-885-5p	60468	BACH2	-0.50	2.14E-07	3.60E-02
hsa-miR-767-3p	9776	ATG13	-0.50	2.23E-07	3.75E-02
hsa-miR-338-5p	4330	MN1	-0.50	2.23E-07	3.76E-02
hsa-miR-206	6542	SLC7A2	-0.50	2.25E-07	3.77E-02
hsa-miR-301b	8976	WASL	-0.50	2.28E-07	3.83E-02
hsa-miR-216a	83941	TM2D1	-0.50	2.28E-07	3.84E-02
hsa-miR-206	7095	SEC62	-0.50	2.29E-07	3.85E-02
hsa-miR-618	440279	UNC13C	-0.50	2.31E-07	3.87E-02
hsa-miR-206	64327	LMBR1	-0.50	2.31E-07	3.89E-02
hsa-miR-206	9674	KIAA0040	-0.50	2.32E-07	3.90E-02
hsa-miR-3164	89857	KLHL6	-0.50	2.36E-07	3.97E-02
hsa-miR-423-5p	10636	RGS14	-0.50	2.36E-07	3.97E-02
hsa-miR-654-5p	56850	GRIPAP1	-0.50	2.37E-07	3.98E-02
hsa-miR-423-5p	8314	BAP1	-0.50	2.43E-07	4.08E-02
hsa-miR-301b	80262	C16orf70	-0.50	2.44E-07	4.09E-02
hsa-miR-330-3p	1809	DPYSL3	-0.50	2.46E-07	4.13E-02
hsa-miR-885-5p	1112	FOXN3	-0.50	2.46E-07	4.14E-02
hsa-miR-618	9743	ARHGAP32	-0.50	2.50E-07	4.19E-02
hsa-miR-885-5p	8573	CASK	-0.50	2.53E-07	4.25E-02
hsa-miR-767-5p	84641	HIATL1	-0.50	2.54E-07	4.26E-02
hsa-miR-219-1-3p	124817	CNTD1	-0.49	2.59E-07	4.35E-02
hsa-miR-501-3p	481	ATP1B1	-0.49	2.73E-07	4.58E-02
hsa-miR-206	5793	PTPRG	-0.49	2.79E-07	4.69E-02
hsa-miR-330-3p	84668	FAM126A	-0.49	2.82E-07	4.74E-02
hsa-miR-767-5p	57605	PITPNM2	-0.49	2.92E-07	4.90E-02
hsa-miR-767-3p	1850	DUSP8	-0.49	2.95E-07	4.96E-02

hsa-miR-767-5p	57620	STIM2	0.49	2.88E-07	4.83E-02
hsa-miR-1284	3148	HMGB2	0.49	2.86E-07	4.81E-02
hsa-miR-217	50515	CHST11	0.49	2.75E-07	4.62E-02
hsa-miR-216a	4883	NPR3	0.49	2.73E-07	4.59E-02
hsa-miR-767-5p	8202	NCOA3	0.50	2.53E-07	4.24E-02
hsa-miR-216a	64375	IKZF4	0.50	2.49E-07	4.19E-02
hsa-miR-330-3p	159090	FAM122B	0.50	2.49E-07	4.19E-02
hsa-miR-330-3p	83699	SH3BGRL2	0.50	2.45E-07	4.13E-02
hsa-miR-330-3p	5604	MAP2K1	0.50	2.41E-07	4.06E-02
hsa-miR-892a	1112	FOXN3	0.50	2.38E-07	4.00E-02
hsa-miR-301b	23253	ANKRD12	0.50	2.35E-07	3.95E-02
hsa-miR-618	29994	BAZ2B	0.50	2.35E-07	3.95E-02
hsa-miR-767-3p	5891	MOK	0.50	2.35E-07	3.94E-02
hsa-miR-330-3p	5813	PURA	0.50	2.33E-07	3.91E-02
hsa-miR-330-3p	2892	GRIA3	0.50	2.29E-07	3.86E-02
hsa-miR-301b	153830	RNF145	0.50	2.21E-07	3.71E-02
hsa-miR-3126-5p	10620	ARID3B	0.50	2.09E-07	3.52E-02
hsa-miR-125a-3p	55421	C17orf85	0.50	1.97E-07	3.30E-02
hsa-miR-301b	57146	TMEM159	0.50	1.96E-07	3.29E-02
hsa-miR-206	894	CCND2	0.50	1.92E-07	3.23E-02
hsa-miR-206	83892	KCTD10	0.50	1.91E-07	3.22E-02
hsa-miR-216b	55216	C11orf57	0.50	1.85E-07	3.12E-02
hsa-miR-206	360023	ZBTB41	0.50	1.82E-07	3.07E-02
hsa-miR-216a	55635	DEPDC1	0.50	1.81E-07	3.04E-02
hsa-miR-301b	80727	TTYH3	0.50	1.74E-07	2.93E-02
hsa-miR-140-3p	6385	SDC4	0.50	1.70E-07	2.86E-02
hsa-miR-1276	8336	HIST1H2AI	0.50	1.70E-07	2.85E-02
hsa-miR-1276	8336	HIST1H2AK	0.50	1.70E-07	2.85E-02
hsa-miR-1276	8336	HIST1H2AL	0.50	1.70E-07	2.85E-02
hsa-miR-1276	8336	HIST1H2AM	0.50	1.70E-07	2.85E-02
hsa-miR-1276	8336	HIST1H2AG	0.50	1.70E-07	2.85E-02
hsa-miR-330-3p	9320	TRIP12	0.50	1.69E-07	2.84E-02
hsa-miR-548y	253512	SLC25A30	0.50	1.67E-07	2.81E-02
hsa-miR-1276	58487	CREBZF	0.50	1.65E-07	2.78E-02
hsa-miR-296-3p	3151	HMGN2	0.50	1.63E-07	2.75E-02
hsa-miR-767-5p	89796	NAV1	0.50	1.59E-07	2.68E-02
hsa-miR-1276	55609	ZNF280C	0.50	1.58E-07	2.65E-02
hsa-miR-767-5p	84146	ZNF644	0.50	1.57E-07	2.64E-02
hsa-miR-1276	51111	SUV420H1	0.50	1.56E-07	2.63E-02
hsa-miR-301b	54617	INO80	0.50	1.55E-07	2.60E-02
hsa-miR-216b	55671	SMEK1	0.50	1.51E-07	2.55E-02
hsa-miR-301b	4952	OCRL	0.50	1.50E-07	2.52E-02
hsa-miR-618	144348	ZNF664	0.50	1.48E-07	2.49E-02
hsa-miR-206	7776	ZNF236	0.50	1.45E-07	2.44E-02
hsa-miR-767-3p	57727	NCOA5	0.50	1.43E-07	2.40E-02
hsa-miR-330-3p	2554	GABRA1	0.50	1.43E-07	2.40E-02
hsa-miR-296-3p	22834	ZNF652	0.50	1.42E-07	2.40E-02
hsa-miR-301b	64093	SMOC1	0.50	1.41E-07	2.37E-02
hsa-miR-330-3p	6653	SORL1	0.50	1.41E-07	2.37E-02

hsa-miR-767-5p	4893	NRAS	0.50	1.40E-07	2.35E-02
hsa-miR-216a	23211	ZC3H4	0.50	1.39E-07	2.34E-02
hsa-miR-301b	55667	DENND4C	0.50	1.34E-07	2.26E-02
hsa-miR-767-5p	340719	NANOS1	0.50	1.34E-07	2.25E-02
hsa-miR-1270	64919	BCL11B	0.50	1.34E-07	2.25E-02
hsa-miR-1270	64919	BCL11B	0.50	1.34E-07	2.25E-02
hsa-miR-301b	7046	TGFBR1	0.50	1.34E-07	2.25E-02
hsa-miR-216a	51	ACOX1	0.51	1.30E-07	2.18E-02
hsa-miR-330-3p	23167	EFR3A	0.51	1.24E-07	2.08E-02
hsa-miR-2113	9880	ZBTB39	0.51	1.24E-07	2.08E-02
hsa-miR-1276	25957	PNISR	0.51	1.23E-07	2.06E-02
hsa-miR-216a	55660	PRPF40A	0.51	1.22E-07	2.06E-02
hsa-miR-216a	63915	BLOC1S5	0.51	1.22E-07	2.05E-02
hsa-miR-301b	57561	ARRDC3	0.51	1.21E-07	2.04E-02
hsa-miR-330-3p	339983	NAT8L	0.51	1.21E-07	2.03E-02
hsa-miR-206	7057	THBS1	0.51	1.20E-07	2.03E-02
hsa-miR-2113	6938	TCF12	0.51	1.16E-07	1.95E-02
hsa-miR-216b	1874	E2F4	0.51	1.15E-07	1.93E-02
hsa-miR-301b	57649	PHF12	0.51	1.14E-07	1.91E-02
hsa-miR-618	11278	KLF12	0.51	1.12E-07	1.89E-02
hsa-miR-3663-3p	7003	TEAD1	0.51	1.11E-07	1.86E-02
hsa-miR-618	4214	MAP3K1	0.51	1.08E-07	1.81E-02
hsa-miR-216b	25932	CLIC4	0.51	1.06E-07	1.78E-02
hsa-miR-767-5p	421	ARVCF	0.51	1.05E-07	1.77E-02
hsa-miR-767-5p	5980	REV3L	0.51	1.04E-07	1.76E-02
hsa-miR-767-5p	4774	NFIA	0.51	1.04E-07	1.75E-02
hsa-miR-301b	28514	DLL1	0.51	1.04E-07	1.75E-02
hsa-miR-206	4686	NCBP1	0.51	1.03E-07	1.73E-02
hsa-miR-301b	29883	CNOT7	0.51	1.01E-07	1.70E-02
hsa-miR-217	167227	DCP2	0.51	9.83E-08	1.65E-02
hsa-miR-448	5015	OTX2	0.51	9.51E-08	1.60E-02
hsa-miR-654-5p	672	BRCA1	0.51	9.49E-08	1.60E-02
hsa-miR-767-3p	54861	SNRK	0.51	9.46E-08	1.59E-02
hsa-miR-618	4301	MLLT4	0.51	9.33E-08	1.57E-02
hsa-miR-206	10228	STX6	0.51	9.26E-08	1.56E-02
hsa-miR-301b	90	ACVR1	0.51	9.11E-08	1.53E-02
hsa-miR-767-5p	1112	FOXN3	0.51	9.02E-08	1.52E-02
hsa-miR-125a-3p	63027	SLC22A23	0.51	9.02E-08	1.52E-02
hsa-miR-767-5p	23366	KIAA0895	0.51	8.97E-08	1.51E-02
hsa-miR-216b	8697	CDC23	0.51	8.68E-08	1.46E-02
hsa-miR-767-3p	57510	XPO5	0.51	8.68E-08	1.46E-02
hsa-miR-885-5p	55742	PARVA	0.51	8.67E-08	1.46E-02
hsa-miR-1276	653604	HIST2H3A	0.51	8.66E-08	1.46E-02
hsa-miR-1276	653604	HIST2H3C	0.51	8.66E-08	1.46E-02
hsa-miR-1276	653604	HIST2H3D	0.51	8.66E-08	1.46E-02
hsa-miR-423-5p	23135	KDM6B	0.51	8.54E-08	1.44E-02
hsa-miR-1275	51029	DESI2	0.51	8.49E-08	1.43E-02
hsa-miR-330-3p	51517	NCKIPSD	0.51	8.29E-08	1.39E-02
hsa-miR-767-5p	9768	KIAA0101	0.51	8.15E-08	1.37E-02

hsa-miR-125a-3p	115825	WDFY2	0.51	8.10E-08	1.36E-02
hsa-miR-296-3p	23135	KDM6B	0.51	8.07E-08	1.36E-02
hsa-miR-3179	92312	MEX3A	0.51	7.58E-08	1.27E-02
hsa-miR-3179	92312	MEX3A	0.51	7.58E-08	1.27E-02
hsa-miR-3179	92312	MEX3A	0.51	7.58E-08	1.27E-02
hsa-miR-301b	546	ATRX	0.51	7.55E-08	1.27E-02
hsa-miR-206	23213	SULF1	0.51	7.29E-08	1.23E-02
hsa-miR-301b	23327	NEDD4L	0.51	7.16E-08	1.21E-02
hsa-miR-217	137886	UBXN2B	0.51	7.09E-08	1.19E-02
hsa-miR-301b	8028	MLLT10	0.51	6.99E-08	1.18E-02
hsa-miR-767-3p	6418	SET	0.51	6.92E-08	1.16E-02
hsa-miR-3659	139285	AMER1	0.51	6.89E-08	1.16E-02
hsa-miR-216b	6498	SKIL	0.52	6.54E-08	1.10E-02
hsa-miR-1276	55715	DOK4	0.52	6.36E-08	1.07E-02
hsa-miR-206	8939	FUBP3	0.52	6.34E-08	1.07E-02
hsa-miR-216a	4325	MMP16	0.52	6.34E-08	1.07E-02
hsa-miR-216a	23135	KDM6B	0.52	6.17E-08	1.04E-02
hsa-miR-216a	25836	NIPBL	0.52	6.13E-08	1.03E-02
hsa-miR-618	84662	GLIS2	0.52	6.13E-08	1.03E-02
hsa-miR-206	8324	FZD7	0.52	6.08E-08	1.02E-02
hsa-miR-330-3p	158471	PRUNE2	0.52	6.06E-08	1.02E-02
hsa-miR-206	222194	RSBN1L	0.52	6.06E-08	1.02E-02
hsa-miR-767-3p	4330	MN1	0.52	6.03E-08	1.02E-02
hsa-miR-125a-3p	84465	MEGF11	0.52	5.99E-08	1.01E-02
hsa-miR-301b	26960	NBEA	0.52	5.93E-08	9.98E-03
hsa-miR-301b	23040	MYT1L	0.52	5.88E-08	9.89E-03
hsa-miR-767-3p	26115	TANC2	0.52	5.86E-08	9.87E-03
hsa-miR-216a	59353	TMEM35	0.52	5.80E-08	9.76E-03
hsa-miR-217	55454	CSGALNACT2	0.52	5.77E-08	9.71E-03
hsa-miR-3659	135295	SRSF12	0.52	5.56E-08	9.36E-03
hsa-miR-330-3p	7504	XK	0.52	5.55E-08	9.33E-03
hsa-miR-301b	9294	S1PR2	0.52	5.49E-08	9.24E-03
hsa-miR-767-3p	10137	RBM12	0.52	5.47E-08	9.21E-03
hsa-miR-618	84668	FAM126A	0.52	5.41E-08	9.11E-03
hsa-miR-216a	607	BCL9	0.52	5.40E-08	9.08E-03
hsa-miR-330-3p	57460	PPM1H	0.52	5.34E-08	8.99E-03
hsa-miR-767-5p	54790	TET2	0.52	5.34E-08	8.98E-03
hsa-miR-551a	112755	STX1B	0.52	5.22E-08	8.78E-03
hsa-miR-216a	5062	PAK2	0.52	5.09E-08	8.57E-03
hsa-miR-767-5p	8899	PRPF4B	0.52	5.08E-08	8.55E-03
hsa-miR-301b	200424	TET3	0.52	5.02E-08	8.44E-03
hsa-miR-216b	9857	CEP350	0.52	5.00E-08	8.42E-03
hsa-miR-767-5p	3157	HMGCS1	0.52	4.99E-08	8.40E-03
hsa-miR-3659	23119	HIC2	0.52	4.99E-08	8.40E-03
hsa-miR-618	6659	SOX4	0.52	4.98E-08	8.37E-03
hsa-miR-301b	9098	USP6	0.52	4.92E-08	8.27E-03
hsa-miR-767-5p	55728	N4BP2	0.52	4.82E-08	8.11E-03
hsa-miR-330-3p	374986	FAM73A	0.52	4.75E-08	8.00E-03
hsa-miR-767-5p	6996	TDG	0.52	4.71E-08	7.92E-03

hsa-miR-206	7572	ZNF24	0.52	4.68E-08	7.88E-03
hsa-miR-217	2044	EPHA5	0.52	4.68E-08	7.88E-03
hsa-miR-423-5p	114794	ELFN2	0.52	4.59E-08	7.72E-03
hsa-miR-767-3p	995	CDC25C	0.52	4.58E-08	7.71E-03
hsa-miR-216b	10018	BCL2L11	0.52	4.58E-08	7.71E-03
hsa-miR-767-5p	1525	CXADR	0.52	4.54E-08	7.64E-03
hsa-miR-206	285590	SH3PXD2B	0.52	4.49E-08	7.56E-03
hsa-miR-618	55205	ZNF532	0.52	4.45E-08	7.49E-03
hsa-miR-892a	4899	NRF1	0.52	4.35E-08	7.33E-03
hsa-miR-217	283464	GXYLT1	0.52	4.32E-08	7.27E-03
hsa-miR-330-3p	2277	FIGF	0.52	4.30E-08	7.24E-03
hsa-miR-206	153830	RNF145	0.52	4.18E-08	7.04E-03
hsa-miR-296-3p	55634	KRBOX4	0.52	4.11E-08	6.92E-03
hsa-miR-23c	2289	FKBP5	0.52	4.11E-08	6.92E-03
hsa-miR-301b	152006	RNF38	0.52	4.05E-08	6.82E-03
hsa-miR-206	60682	SMAP1	0.52	4.03E-08	6.79E-03
hsa-miR-217	10659	CELF2	0.52	3.75E-08	6.32E-03
hsa-miR-206	23446	SLC44A1	0.52	3.73E-08	6.29E-03
hsa-miR-206	7849	PAX8	0.52	3.73E-08	6.28E-03
hsa-miR-1270	55636	CHD7	0.52	3.64E-08	6.13E-03
hsa-miR-1270	55636	CHD7	0.52	3.64E-08	6.13E-03
hsa-miR-206	1611	DAP	0.52	3.48E-08	5.86E-03
hsa-miR-206	5928	RBBP4	0.52	3.45E-08	5.81E-03
hsa-miR-3663-3p	1021	CDK6	0.52	3.45E-08	5.81E-03
hsa-miR-216b	51762	RAB8B	0.52	3.41E-08	5.75E-03
hsa-miR-216a	4756	NEO1	0.52	3.39E-08	5.70E-03
hsa-miR-767-3p	200424	TET3	0.53	3.27E-08	5.50E-03
hsa-miR-206	113263	GLCCI1	0.53	3.21E-08	5.41E-03
hsa-miR-330-3p	340419	RSPO2	0.53	3.15E-08	5.31E-03
hsa-miR-206	1655	DDX5	0.53	3.12E-08	5.25E-03
hsa-miR-217	4170	MCL1	0.53	3.10E-08	5.22E-03
hsa-miR-1276	147991	DPY19L3	0.53	3.07E-08	5.16E-03
hsa-miR-206	6942	TCF20	0.53	3.04E-08	5.12E-03
hsa-miR-206	7328	UBE2H	0.53	3.00E-08	5.05E-03
hsa-miR-2113	1627	DBN1	0.53	2.93E-08	4.94E-03
hsa-miR-301b	29922	NME7	0.53	2.85E-08	4.81E-03
hsa-miR-217	22856	CHSY1	0.53	2.85E-08	4.79E-03
hsa-miR-330-3p	203197	C9orf91	0.53	2.81E-08	4.73E-03
hsa-miR-301b	7552	ZNF711	0.53	2.77E-08	4.66E-03
hsa-miR-301b	387640	SKIDA1	0.53	2.63E-08	4.43E-03
hsa-miR-301b	3720	JARID2	0.53	2.55E-08	4.29E-03
hsa-miR-301b	89797	NAV2	0.53	2.53E-08	4.25E-03
hsa-miR-216a	8289	ARID1A	0.53	2.50E-08	4.21E-03
hsa-miR-1275	57459	GATAD2B	0.53	2.50E-08	4.20E-03
hsa-miR-767-5p	3480	IGF1R	0.53	2.45E-08	4.13E-03
hsa-miR-296-3p	26137	ZBTB20	0.53	2.45E-08	4.12E-03
hsa-miR-767-5p	5565	PRKAB2	0.53	2.40E-08	4.04E-03
hsa-miR-216a	8648	NCOA1	0.53	2.38E-08	4.01E-03
hsa-miR-301b	29127	RACGAP1	0.53	2.33E-08	3.92E-03

hsa-miR-767-3p	55596	ZCCHC8	0.53	2.25E-08	3.79E-03
hsa-miR-330-3p	26056	RAB11FIP5	0.53	2.23E-08	3.76E-03
hsa-miR-767-3p	4848	CNOT2	0.53	2.19E-08	3.68E-03
hsa-miR-301b	10640	EXOC5	0.53	2.12E-08	3.58E-03
hsa-miR-301b	23097	CDK19	0.53	2.10E-08	3.54E-03
hsa-miR-206	27236	ARFIP1	0.53	2.10E-08	3.54E-03
hsa-miR-1276	8354	HIST1H3E	0.53	2.08E-08	3.50E-03
hsa-miR-1276	8354	HIST1H3J	0.53	2.08E-08	3.50E-03
hsa-miR-1276	8354	HIST1H3I	0.53	2.08E-08	3.50E-03
hsa-miR-1276	8354	HIST1H3H	0.53	2.08E-08	3.50E-03
hsa-miR-1276	8354	HIST1H3F	0.53	2.08E-08	3.50E-03
hsa-miR-1276	8354	HIST1H3D	0.53	2.08E-08	3.50E-03
hsa-miR-1276	8354	HIST1H3C	0.53	2.08E-08	3.50E-03
hsa-miR-1276	8354	HIST1H3B	0.53	2.08E-08	3.50E-03
hsa-miR-1276	8354	HIST1H3A	0.53	2.08E-08	3.50E-03
hsa-miR-1276	8354	HIST1H3G	0.53	2.08E-08	3.50E-03
hsa-miR-206	22863	ATG14	0.53	2.02E-08	3.41E-03
hsa-miR-767-5p	65124	SOWAHC	0.53	1.99E-08	3.35E-03
hsa-miR-892a	64105	CENPK	0.53	1.98E-08	3.34E-03
hsa-miR-206	23023	TMCC1	0.53	1.97E-08	3.32E-03
hsa-miR-217	84617	TUBB6	0.53	1.96E-08	3.29E-03
hsa-miR-767-5p	1654	DDX3X	0.53	1.94E-08	3.26E-03
hsa-miR-330-3p	9783	RIMS3	0.53	1.87E-08	3.14E-03
hsa-miR-618	57626	KLHL1	0.53	1.84E-08	3.09E-03
hsa-miR-217	57695	USP37	0.53	1.83E-08	3.09E-03
hsa-miR-216a	11278	KLF12	0.53	1.82E-08	3.07E-03
hsa-miR-301b	55719	FAM178A	0.53	1.81E-08	3.05E-03
hsa-miR-301b	57690	TNRC6C	0.53	1.81E-08	3.05E-03
hsa-miR-449a	1021	CDK6	0.53	1.80E-08	3.04E-03
hsa-miR-1276	4200	ME2	0.53	1.79E-08	3.02E-03
hsa-miR-125a-3p	92105	INTS4	0.53	1.78E-08	3.00E-03
hsa-miR-206	23603	CORO1C	0.53	1.77E-08	2.97E-03
hsa-miR-423-5p	89796	NAV1	0.53	1.76E-08	2.97E-03
hsa-miR-301b	8975	USP13	0.53	1.74E-08	2.93E-03
hsa-miR-217	6996	TDG	0.53	1.72E-08	2.90E-03
hsa-miR-301b	22834	ZNF652	0.53	1.72E-08	2.90E-03
hsa-miR-4295	122042	RXFP2	0.53	1.65E-08	2.79E-03
hsa-miR-301b	6307	MSMO1	0.54	1.65E-08	2.78E-03
hsa-miR-767-5p	51444	RNF138	0.54	1.60E-08	2.69E-03
hsa-miR-618	80312	TET1	0.54	1.59E-08	2.68E-03
hsa-miR-3689a-3p	150864	FAM117B	0.54	1.59E-08	2.67E-03
hsa-miR-767-5p	2941	GSTA4	0.54	1.52E-08	2.56E-03
hsa-miR-618	23279	NUP160	0.54	1.50E-08	2.53E-03
hsa-miR-301b	51072	MEMO1	0.54	1.50E-08	2.52E-03
hsa-miR-216a	9678	PHF14	0.54	1.46E-08	2.46E-03
hsa-miR-219-1-3p	10735	STAG2	0.54	1.46E-08	2.45E-03
hsa-miR-301b	23071	ERP44	0.54	1.42E-08	2.39E-03
hsa-miR-301b	3815	KIT	0.54	1.41E-08	2.38E-03
hsa-miR-301b	26523	AGO1	0.54	1.41E-08	2.38E-03

hsa-miR-217	10371	SEMA3A	0.54	1.41E-08	2.37E-03
hsa-miR-216b	9986	RCE1	0.54	1.35E-08	2.27E-03
hsa-miR-767-5p	23567	ZNF346	0.54	1.32E-08	2.22E-03
hsa-miR-216a	22919	MAPRE1	0.54	1.32E-08	2.22E-03
hsa-miR-1270	22998	LIMCH1	0.54	1.31E-08	2.21E-03
hsa-miR-1270	22998	LIMCH1	0.54	1.31E-08	2.21E-03
hsa-miR-206	7534	YWHAZ	0.54	1.25E-08	2.11E-03
hsa-miR-217	6421	SFPQ	0.54	1.24E-08	2.09E-03
hsa-miR-216a	57216	VANGL2	0.54	1.23E-08	2.08E-03
hsa-miR-767-5p	1316	KLF6	0.54	1.23E-08	2.07E-03
hsa-miR-767-3p	9337	CNOT8	0.54	1.21E-08	2.04E-03
hsa-miR-296-3p	8828	NRP2	0.54	1.21E-08	2.04E-03
hsa-miR-618	6434	TRA2B	0.54	1.20E-08	2.01E-03
hsa-miR-301b	9231	DLG5	0.54	1.18E-08	1.99E-03
hsa-miR-301b	222537	HS3ST5	0.54	1.15E-08	1.94E-03
hsa-miR-217	10560	SLC19A2	0.54	1.10E-08	1.84E-03
hsa-miR-217	11011	TLK2	0.54	1.07E-08	1.81E-03
hsa-miR-217	10730	YME1L1	0.54	1.06E-08	1.79E-03
hsa-miR-301b	6874	TAF4	0.54	1.05E-08	1.77E-03
hsa-miR-618	5936	RBM4	0.54	1.04E-08	1.75E-03
hsa-miR-767-5p	26064	RAI14	0.54	1.03E-08	1.73E-03
hsa-miR-216a	9388	LIPG	0.54	1.01E-08	1.70E-03
hsa-miR-217	8493	PPM1D	0.54	1.01E-08	1.70E-03
hsa-miR-767-5p	80312	TET1	0.54	9.91E-09	1.67E-03
hsa-miR-301b	7321	UBE2D1	0.54	9.86E-09	1.66E-03
hsa-miR-767-5p	3321	IGSF3	0.54	9.75E-09	1.64E-03
hsa-miR-618	4281	MID1	0.54	9.74E-09	1.64E-03
hsa-miR-301b	137886	UBXN2B	0.54	9.66E-09	1.63E-03
hsa-miR-301b	79753	SNIP1	0.54	9.17E-09	1.55E-03
hsa-miR-767-3p	1385	CREB1	0.54	9.03E-09	1.52E-03
hsa-miR-125a-3p	55454	CSGALNACT2	0.54	8.74E-09	1.47E-03
hsa-miR-767-3p	8202	NCOA3	0.54	8.69E-09	1.46E-03
hsa-miR-301b	10018	BCL2L11	0.54	8.63E-09	1.45E-03
hsa-miR-423-5p	155382	VPS37D	0.54	8.53E-09	1.44E-03
hsa-miR-217	23411	SIRT1	0.54	8.43E-09	1.42E-03
hsa-miR-618	7903	ST8SIA4	0.54	8.43E-09	1.42E-03
hsa-miR-301b	7352	UCP3	0.54	8.26E-09	1.39E-03
hsa-miR-1276	64324	NSD1	0.54	8.25E-09	1.39E-03
hsa-miR-217	6335	SCN9A	0.54	8.10E-09	1.37E-03
hsa-miR-217	91746	YTHDC1	0.54	8.05E-09	1.36E-03
hsa-miR-206	23085	ERC1	0.55	7.70E-09	1.30E-03
hsa-miR-301b	6660	SOX5	0.55	7.41E-09	1.25E-03
hsa-miR-206	5756	TWF1	0.55	7.36E-09	1.24E-03
hsa-miR-618	3181	HNRNPA2B1	0.55	7.27E-09	1.22E-03
hsa-miR-767-5p	2201	FBN2	0.55	7.16E-09	1.21E-03
hsa-miR-216b	55870	ASH1L	0.55	6.87E-09	1.16E-03
hsa-miR-301b	8715	NOL4	0.55	6.29E-09	1.06E-03
hsa-miR-206	4214	MAP3K1	0.55	6.22E-09	1.05E-03
hsa-miR-206	253430	IPMK	0.55	5.80E-09	9.78E-04

hsa-miR-330-3p	23136	EPB41L3	0.55	5.78E-09	9.75E-04
hsa-miR-301b	9839	ZEB2	0.55	5.78E-09	9.75E-04
hsa-miR-301b	151963	MB21D2	0.55	5.77E-09	9.72E-04
hsa-miR-767-3p	27443	CECR2	0.55	5.77E-09	9.72E-04
hsa-miR-206	54665	RSBN1	0.55	5.55E-09	9.35E-04
hsa-miR-767-3p	51447	IP6K2	0.55	5.50E-09	9.27E-04
hsa-miR-767-5p	22982	DIP2C	0.55	5.44E-09	9.16E-04
hsa-miR-767-3p	7525	YES1	0.55	5.38E-09	9.07E-04
hsa-miR-206	6426	SRSF1	0.55	5.27E-09	8.88E-04
hsa-miR-206	167227	DCP2	0.55	5.20E-09	8.76E-04
hsa-miR-767-3p	9752	PCDHA9	0.55	5.02E-09	8.46E-04
hsa-miR-301b	744	MPPED2	0.55	4.87E-09	8.21E-04
hsa-miR-301b	23013	SPEN	0.55	4.87E-09	8.20E-04
hsa-miR-206	1266	CNN3	0.55	4.84E-09	8.17E-04
hsa-miR-217	80335	WDR82	0.55	4.81E-09	8.10E-04
hsa-miR-206	50515	CHST11	0.55	4.80E-09	8.09E-04
hsa-miR-216b	1457	CSNK2A1	0.55	4.76E-09	8.02E-04
hsa-miR-1276	11237	RNF24	0.55	4.74E-09	7.99E-04
hsa-miR-301b	7410	VAV2	0.55	4.69E-09	7.91E-04
hsa-miR-767-3p	8879	SGPL1	0.55	4.63E-09	7.80E-04
hsa-miR-767-3p	59353	TMEM35	0.55	4.59E-09	7.74E-04
hsa-miR-206	84271	POLDIP3	0.55	4.51E-09	7.61E-04
hsa-miR-767-3p	6815	STYX	0.55	4.45E-09	7.49E-04
hsa-miR-767-5p	84206	MEX3B	0.55	4.43E-09	7.46E-04
hsa-miR-301b	84458	LCOR	0.55	4.36E-09	7.35E-04
hsa-miR-767-5p	64324	NSD1	0.55	4.33E-09	7.29E-04
hsa-miR-767-5p	91584	PLXNA4	0.55	4.32E-09	7.28E-04
hsa-miR-1275	63973	NEUROG2	0.55	4.05E-09	6.84E-04
hsa-miR-217	114818	KLHL29	0.55	3.93E-09	6.63E-04
hsa-miR-1276	58516	FAM60A	0.55	3.92E-09	6.61E-04
hsa-miR-301b	19	ABCA1	0.55	3.86E-09	6.51E-04
hsa-miR-125a-3p	54934	KANSL2	0.55	3.84E-09	6.47E-04
hsa-miR-216b	8540	AGPS	0.55	3.71E-09	6.25E-04
hsa-miR-1252	154881	KCTD7	0.55	3.71E-09	6.25E-04
hsa-miR-216b	55500	ETNK1	0.55	3.67E-09	6.19E-04
hsa-miR-618	54329	GPR85	0.56	3.58E-09	6.03E-04
hsa-miR-206	5469	MED1	0.56	3.35E-09	5.64E-04
hsa-miR-3126-5p	6666	SOX12	0.56	3.33E-09	5.62E-04
hsa-miR-217	92	ACVR2A	0.56	3.23E-09	5.45E-04
hsa-miR-301b	81846	SBF2	0.56	3.21E-09	5.41E-04
hsa-miR-216a	390980	ZNF805	0.56	3.09E-09	5.21E-04
hsa-miR-216a	51444	RNF138	0.56	3.03E-09	5.11E-04
hsa-miR-216b	403	ARL3	0.56	3.01E-09	5.08E-04
hsa-miR-216b	57396	CLK4	0.56	3.00E-09	5.06E-04
hsa-miR-1254	151742	PPM1L	0.56	3.00E-09	5.06E-04
hsa-miR-1254	151742	PPM1L	0.56	3.00E-09	5.06E-04
hsa-miR-206	23527	ACAP2	0.56	2.98E-09	5.03E-04
hsa-miR-206	5335	PLCG1	0.56	2.98E-09	5.02E-04
hsa-miR-767-5p	9736	USP34	0.56	2.91E-09	4.91E-04

hsa-miR-423-5p	6666	SOX12	0.56	2.90E-09	4.89E-04
hsa-miR-767-5p	151963	MB21D2	0.56	2.87E-09	4.85E-04
hsa-miR-448	1602	DACH1	0.56	2.83E-09	4.77E-04
hsa-miR-216a	23450	SF3B3	0.56	2.74E-09	4.61E-04
hsa-miR-767-3p	149076	ZNF362	0.56	2.70E-09	4.56E-04
hsa-miR-423-5p	4826	NNAT	0.56	2.69E-09	4.54E-04
hsa-miR-484	653319	KIAA0895L	0.56	2.66E-09	4.49E-04
hsa-miR-767-3p	57670	KIAA1549	0.56	2.59E-09	4.37E-04
hsa-miR-125a-3p	7204	TRIO	0.56	2.57E-09	4.33E-04
hsa-miR-206	387640	SKIDA1	0.56	2.53E-09	4.26E-04
hsa-miR-4289	10149	GPR64	0.56	2.52E-09	4.26E-04
hsa-miR-767-3p	9889	ZBED4	0.56	2.37E-09	4.00E-04
hsa-miR-301b	9794	MAML1	0.56	2.36E-09	3.98E-04
hsa-miR-216b	51	ACOX1	0.56	2.34E-09	3.95E-04
hsa-miR-618	221833	SP8	0.56	2.33E-09	3.93E-04
hsa-miR-301b	2186	BPTF	0.56	2.30E-09	3.88E-04
hsa-miR-206	10743	RAI1	0.56	2.29E-09	3.85E-04
hsa-miR-1275	4330	MN1	0.56	2.26E-09	3.81E-04
hsa-miR-216b	137970	UNC5D	0.56	2.25E-09	3.79E-04
hsa-miR-301b	3842	TNPO1	0.56	2.15E-09	3.63E-04
hsa-miR-423-5p	23119	HIC2	0.56	2.15E-09	3.62E-04
hsa-miR-217	84458	LCOR	0.56	2.07E-09	3.49E-04
hsa-miR-767-5p	7428	VHL	0.56	1.98E-09	3.33E-04
hsa-miR-125a-3p	55752	11-Sep	0.56	1.90E-09	3.21E-04
hsa-miR-217	166968	MIER3	0.56	1.76E-09	2.97E-04
hsa-miR-767-5p	862	RUNX1T1	0.56	1.69E-09	2.85E-04
hsa-miR-125a-3p	10644	IGF2BP2	0.56	1.67E-09	2.81E-04
hsa-miR-217	51747	LUC7L3	0.56	1.66E-09	2.80E-04
hsa-miR-216b	22913	RALY	0.57	1.64E-09	2.77E-04
hsa-miR-301b	149420	PDIK1L	0.57	1.59E-09	2.68E-04
hsa-miR-301b	6925	TCF4	0.57	1.49E-09	2.52E-04
hsa-miR-301b	51441	YTHDF2	0.57	1.48E-09	2.50E-04
hsa-miR-301b	619279	ZNF704	0.57	1.48E-09	2.49E-04
hsa-miR-301b	57162	PELI1	0.57	1.47E-09	2.48E-04
hsa-miR-206	6667	SP1	0.57	1.37E-09	2.32E-04
hsa-miR-216b	205717	KIAA2018	0.57	1.34E-09	2.25E-04
hsa-miR-217	114799	ESCO1	0.57	1.33E-09	2.24E-04
hsa-miR-767-5p	3038	HAS3	0.57	1.32E-09	2.22E-04
hsa-miR-301b	58508	MLL3	0.57	1.29E-09	2.18E-04
hsa-miR-301b	254065	BRWD3	0.57	1.29E-09	2.18E-04
hsa-miR-125a-3p	1808	DPYSL2	0.57	1.28E-09	2.16E-04
hsa-miR-767-5p	23426	GRIP1	0.57	1.28E-09	2.15E-04
hsa-miR-206	605	BCL7A	0.57	1.26E-09	2.13E-04
hsa-miR-767-3p	64789	EXO5	0.57	1.20E-09	2.03E-04
hsa-miR-767-3p	54790	TET2	0.57	1.19E-09	2.01E-04
hsa-miR-301b	26122	EPC2	0.57	1.18E-09	1.99E-04
hsa-miR-217	51735	RAPGEF6	0.57	1.15E-09	1.94E-04
hsa-miR-301b	865	CBFB	0.57	1.14E-09	1.93E-04
hsa-miR-767-5p	3658	IREB2	0.57	1.09E-09	1.84E-04

hsa-miR-301b	80311	KLHL15	0.57	1.03E-09	1.74E-04
hsa-miR-219-1-3p	51072	MEMO1	0.57	1.03E-09	1.74E-04
hsa-miR-216b	6925	TCF4	0.57	9.75E-10	1.65E-04
hsa-miR-301b	908	CCT6A	0.57	9.64E-10	1.63E-04
hsa-miR-301b	4194	MDM4	0.57	9.14E-10	1.54E-04
hsa-miR-296-3p	57482	KIAA1211	0.57	9.06E-10	1.53E-04
hsa-miR-301b	399664	MEX3D	0.57	8.61E-10	1.45E-04
hsa-miR-125a-3p	9246	UBE2L6	0.57	8.48E-10	1.43E-04
hsa-miR-301b	60468	BACH2	0.57	8.44E-10	1.42E-04
hsa-miR-1275	4841	NONO	0.57	8.32E-10	1.40E-04
hsa-miR-767-5p	4678	NASP	0.57	7.78E-10	1.31E-04
hsa-miR-217	10846	PDE10A	0.57	7.52E-10	1.27E-04
hsa-miR-301b	57459	GATAD2B	0.57	7.46E-10	1.26E-04
hsa-miR-618	92312	MEX3A	0.58	6.99E-10	1.18E-04
hsa-miR-301b	192670	AGO4	0.58	6.93E-10	1.17E-04
hsa-miR-301b	9770	RASSF2	0.58	6.93E-10	1.17E-04
hsa-miR-219-1-3p	114794	ELFN2	0.58	6.87E-10	1.16E-04
hsa-miR-125a-3p	57486	NLN	0.58	6.78E-10	1.14E-04
hsa-miR-206	23032	USP33	0.58	6.69E-10	1.13E-04
hsa-miR-301b	23243	ANKRD28	0.58	6.66E-10	1.12E-04
hsa-miR-296-3p	89796	NAV1	0.58	6.56E-10	1.11E-04
hsa-miR-216b	51479	ANKFY1	0.58	6.45E-10	1.09E-04
hsa-miR-206	6599	SMARCC1	0.58	6.38E-10	1.08E-04
hsa-miR-206	140838	NANP	0.58	6.29E-10	1.06E-04
hsa-miR-217	79813	EHMT1	0.58	6.21E-10	1.05E-04
hsa-miR-301b	6659	SOX4	0.58	6.15E-10	1.04E-04
hsa-miR-301b	7804	LRP8	0.58	6.11E-10	1.03E-04
hsa-miR-217	91584	PLXNA4	0.58	5.94E-10	1.00E-04
hsa-miR-301b	26115	TANC2	0.58	5.87E-10	9.90E-05
hsa-miR-301b	1948	EFNB2	0.58	5.63E-10	9.49E-05
hsa-miR-301b	166968	MIER3	0.58	5.60E-10	9.44E-05
hsa-miR-301b	65267	WNK3	0.58	5.57E-10	9.39E-05
hsa-miR-301b	153241	CEP120	0.58	5.50E-10	9.29E-05
hsa-miR-767-5p	10512	SEMA3C	0.58	5.35E-10	9.03E-05
hsa-miR-767-3p	653319	KIAA0895L	0.58	5.23E-10	8.82E-05
hsa-miR-125a-3p	1316	KLF6	0.58	4.99E-10	8.42E-05
hsa-miR-216b	2551	GABPA	0.58	4.95E-10	8.36E-05
hsa-miR-301b	57186	RALGAPA2	0.58	4.65E-10	7.85E-05
hsa-miR-301b	23607	CD2AP	0.58	4.56E-10	7.69E-05
hsa-miR-767-3p	54329	GPR85	0.58	4.32E-10	7.28E-05
hsa-miR-217	2201	FBN2	0.58	4.30E-10	7.25E-05
hsa-miR-301b	23503	ZFYVE26	0.58	4.17E-10	7.04E-05
hsa-miR-206	9658	ZNF516	0.58	4.10E-10	6.92E-05
hsa-miR-301b	10389	SCML2	0.58	3.84E-10	6.47E-05
hsa-miR-206	55705	IPO9	0.58	3.62E-10	6.12E-05
hsa-miR-767-5p	4613	MYCN	0.58	3.51E-10	5.92E-05
hsa-miR-301b	7837	PXDN	0.58	3.49E-10	5.88E-05
hsa-miR-217	9658	ZNF516	0.58	3.40E-10	5.74E-05
hsa-miR-219-1-3p	7371	UCK2	0.58	3.34E-10	5.64E-05

hsa-miR-767-3p	196528	ARID2	0.58	3.23E-10	5.45E-05
hsa-miR-767-3p	9682	KDM4A	0.59	3.07E-10	5.19E-05
hsa-miR-301b	10472	ZBTB18	0.59	2.93E-10	4.94E-05
hsa-miR-301b	26136	TES	0.59	2.80E-10	4.73E-05
hsa-miR-301b	55727	BTBD7	0.59	2.79E-10	4.71E-05
hsa-miR-206	5080	PAX6	0.59	2.77E-10	4.67E-05
hsa-miR-217	23211	ZC3H4	0.59	2.76E-10	4.65E-05
hsa-miR-125a-3p	54454	ATAD2B	0.59	2.73E-10	4.61E-05
hsa-miR-301b	7976	FZD3	0.59	2.57E-10	4.34E-05
hsa-miR-125a-3p	10238	DCAF7	0.59	2.26E-10	3.82E-05
hsa-miR-125a-3p	23545	ATP6V0A2	0.59	2.14E-10	3.61E-05
hsa-miR-217	22858	ICK	0.59	2.11E-10	3.56E-05
hsa-miR-206	23005	MAPKBP1	0.59	2.10E-10	3.55E-05
hsa-miR-301b	1385	CREB1	0.59	2.07E-10	3.50E-05
hsa-miR-216a	23331	TTC28	0.59	2.05E-10	3.46E-05
hsa-miR-125a-3p	63973	NEUROG2	0.59	1.99E-10	3.36E-05
hsa-miR-217	286410	ATP11C	0.59	1.71E-10	2.89E-05
hsa-miR-217	2334	AFF2	0.59	1.65E-10	2.79E-05
hsa-miR-206	2218	FKTN	0.59	1.65E-10	2.79E-05
hsa-miR-219-1-3p	80143	SIKE1	0.59	1.65E-10	2.78E-05
hsa-miR-206	220988	HNRNPA3	0.59	1.60E-10	2.70E-05
hsa-miR-217	22998	LIMCH1	0.59	1.51E-10	2.56E-05
hsa-miR-217	53335	BCL11A	0.59	1.50E-10	2.53E-05
hsa-miR-301b	57224	NHSL1	0.59	1.45E-10	2.44E-05
hsa-miR-767-5p	342357	ZKSCAN2	0.59	1.44E-10	2.43E-05
hsa-miR-301b	65055	REEP1	0.59	1.41E-10	2.38E-05
hsa-miR-449a	60436	TGIF2	0.59	1.31E-10	2.21E-05
hsa-miR-217	2665	GDI2	0.60	1.22E-10	2.05E-05
hsa-miR-206	54816	ZNF280D	0.60	1.18E-10	1.99E-05
hsa-miR-1276	8339	HIST1H2BC	0.60	1.17E-10	1.98E-05
hsa-miR-1276	8339	HIST1H2BI	0.60	1.17E-10	1.98E-05
hsa-miR-1276	8339	HIST1H2BG	0.60	1.17E-10	1.98E-05
hsa-miR-1276	8339	HIST1H2BE	0.60	1.17E-10	1.98E-05
hsa-miR-1276	8339	HIST1H2BF	0.60	1.17E-10	1.98E-05
hsa-miR-206	3329	HSPD1	0.60	1.15E-10	1.94E-05
hsa-miR-216b	6432	SRSF7	0.60	1.06E-10	1.80E-05
hsa-miR-767-3p	26472	PPP1R14B	0.60	1.06E-10	1.80E-05
hsa-miR-301b	9859	CEP170	0.60	1.01E-10	1.71E-05
hsa-miR-217	2898	GRIK2	0.60	9.26E-11	1.56E-05
hsa-miR-206	51366	UBR5	0.60	9.02E-11	1.52E-05
hsa-miR-423-5p	6723	SRM	0.60	8.60E-11	1.45E-05
hsa-miR-301b	29967	LRP12	0.60	8.46E-11	1.43E-05
hsa-miR-216b	8573	CASK	0.60	7.82E-11	1.32E-05
hsa-miR-301b	64864	RFX7	0.60	7.40E-11	1.25E-05
hsa-miR-423-5p	11078	TRIOBP	0.60	7.11E-11	1.20E-05
hsa-miR-206	359845	FAM101B	0.60	7.10E-11	1.20E-05
hsa-miR-301b	57472	CNOT6	0.60	6.99E-11	1.18E-05
hsa-miR-206	51122	COMMD2	0.60	6.74E-11	1.14E-05
hsa-miR-206	8503	PIK3R3	0.60	6.60E-11	1.12E-05

hsa-miR-216a	9768	KIAA0101	0.60	6.41E-11	1.08E-05
hsa-miR-219-1-3p	22834	ZNF652	0.60	6.26E-11	1.06E-05
hsa-miR-301b	57482	KIAA1211	0.60	6.02E-11	1.02E-05
hsa-miR-219-1-3p	493869	GPX8	0.60	5.91E-11	9.99E-06
hsa-miR-423-5p	55351	STK32B	0.60	5.91E-11	9.98E-06
hsa-miR-206	3796	KIF2A	0.60	5.85E-11	9.88E-06
hsa-miR-301b	57448	BIRC6	0.60	5.62E-11	9.50E-06
hsa-miR-301b	5454	POU3F2	0.60	5.49E-11	9.27E-06
hsa-miR-125a-3p	25850	ZNF345	0.60	5.36E-11	9.05E-06
hsa-miR-301b	57179	KIAA1191	0.61	5.12E-11	8.64E-06
hsa-miR-216b	4801	NFYB	0.61	5.03E-11	8.50E-06
hsa-miR-301b	1808	DPYSL2	0.61	4.97E-11	8.40E-06
hsa-miR-767-5p	60468	BACH2	0.61	4.90E-11	8.28E-06
hsa-miR-423-5p	23060	ZNF609	0.61	4.62E-11	7.81E-06
hsa-miR-206	1665	DHX15	0.61	4.58E-11	7.73E-06
hsa-miR-206	27112	FAM155B	0.61	4.45E-11	7.52E-06
hsa-miR-206	340533	KIAA2022	0.61	4.37E-11	7.39E-06
hsa-miR-125a-3p	55743	CHFR	0.61	4.37E-11	7.38E-06
hsa-miR-301b	344148	NCKAP5	0.61	4.26E-11	7.20E-06
hsa-miR-301b	23181	DIP2A	0.61	4.15E-11	7.00E-06
hsa-miR-301b	54467	ANKIB1	0.61	4.12E-11	6.96E-06
hsa-miR-423-5p	60468	BACH2	0.61	3.74E-11	6.32E-06
hsa-miR-216b	25853	DCAF12	0.61	3.67E-11	6.19E-06
hsa-miR-301b	862	RUNX1T1	0.61	3.61E-11	6.09E-06
hsa-miR-219-1-3p	221935	SDK1	0.61	3.59E-11	6.06E-06
hsa-miR-301b	3321	IGSF3	0.61	3.58E-11	6.05E-06
hsa-miR-217	25836	NIPBL	0.61	3.53E-11	5.96E-06
hsa-miR-301b	23139	MAST2	0.61	3.41E-11	5.76E-06
hsa-miR-301b	51111	SUV420H1	0.61	3.33E-11	5.62E-06
hsa-miR-206	10919	EHMT2	0.61	3.14E-11	5.30E-06
hsa-miR-216a	9925	ZBTB5	0.61	2.90E-11	4.90E-06
hsa-miR-206	6428	SRSF3	0.61	2.81E-11	4.74E-06
hsa-miR-216b	1809	DPYSL3	0.61	2.76E-11	4.66E-06
hsa-miR-301b	8462	KLF11	0.61	2.73E-11	4.60E-06
hsa-miR-217	220988	HNRNPA3	0.61	2.56E-11	4.33E-06
hsa-miR-301b	10742	RAI2	0.61	2.56E-11	4.32E-06
hsa-miR-301b	7551	ZNF3	0.61	2.43E-11	4.10E-06
hsa-miR-767-3p	3720	JARID2	0.61	2.36E-11	3.98E-06
hsa-miR-206	4082	MARCKS	0.61	2.32E-11	3.92E-06
hsa-miR-301b	255967	PAN3	0.61	2.30E-11	3.89E-06
hsa-miR-301b	4760	NEUROD1	0.61	2.30E-11	3.89E-06
hsa-miR-1276	3007	HIST1H1D	0.61	2.28E-11	3.85E-06
hsa-miR-301b	6738	TROVE2	0.61	2.16E-11	3.64E-06
hsa-miR-301b	29068	ZBTB44	0.61	2.12E-11	3.58E-06
hsa-miR-1276	3012	HIST1H2AE	0.62	2.05E-11	3.47E-06
hsa-miR-1276	3012	HIST1H2AB	0.62	2.05E-11	3.47E-06
hsa-miR-206	23450	SF3B3	0.62	2.03E-11	3.43E-06
hsa-miR-206	53335	BCL11A	0.62	1.94E-11	3.27E-06
hsa-miR-206	6434	TRA2B	0.62	1.89E-11	3.20E-06

hsa-miR-301b	57631	LRCH2	0.62	1.69E-11	2.86E-06
hsa-miR-206	26960	NBEA	0.62	1.47E-11	2.48E-06
hsa-miR-216a	92312	MEX3A	0.62	1.28E-11	2.16E-06
hsa-miR-217	84168	ANTXR1	0.62	1.28E-11	2.16E-06
hsa-miR-206	91584	PLXNA4	0.62	1.20E-11	2.02E-06
hsa-miR-206	256643	CXorf23	0.62	1.19E-11	2.02E-06
hsa-miR-767-3p	79674	VEPH1	0.62	1.17E-11	1.97E-06
hsa-miR-1276	8342	HIST1H2BM	0.62	1.14E-11	1.92E-06
hsa-miR-206	64762	GAREM	0.62	1.00E-11	1.69E-06
hsa-miR-216b	7410	VAV2	0.62	9.04E-12	1.53E-06
hsa-miR-217	80816	ASXL3	0.62	8.14E-12	1.38E-06
hsa-miR-206	3190	HNRNPK	0.63	7.24E-12	1.22E-06
hsa-miR-216b	167227	DCP2	0.63	7.03E-12	1.19E-06
hsa-miR-216b	4774	NFIA	0.63	5.83E-12	9.85E-07
hsa-miR-219-1-3p	58487	CREBZF	0.63	5.82E-12	9.83E-07
hsa-miR-217	196528	ARID2	0.63	5.48E-12	9.26E-07
hsa-miR-219-1-3p	2335	FN1	0.63	5.25E-12	8.87E-07
hsa-miR-217	55609	ZNF280C	0.63	5.12E-12	8.65E-07
hsa-miR-217	57337	SENP7	0.63	5.10E-12	8.62E-07
hsa-miR-423-5p	5569	PKIA	0.63	5.08E-12	8.59E-07
hsa-miR-301b	7764	ZNF217	0.63	5.01E-12	8.47E-07
hsa-miR-217	60468	BACH2	0.63	4.60E-12	7.77E-07
hsa-miR-217	7072	TIA1	0.63	4.42E-12	7.46E-07
hsa-miR-206	4775	NFATC3	0.63	3.87E-12	6.55E-07
hsa-miR-206	23181	DIP2A	0.63	3.59E-12	6.06E-07
hsa-miR-206	7267	TTC3	0.63	3.41E-12	5.77E-07
hsa-miR-1276	8356	HIST1H3D	0.63	3.23E-12	5.46E-07
hsa-miR-1276	8356	HIST1H3J	0.63	3.23E-12	5.46E-07
hsa-miR-1276	8356	HIST1H3I	0.63	3.23E-12	5.46E-07
hsa-miR-1276	8356	HIST1H3H	0.63	3.23E-12	5.46E-07
hsa-miR-1276	8356	HIST1H3G	0.63	3.23E-12	5.46E-07
hsa-miR-1276	8356	HIST1H3E	0.63	3.23E-12	5.46E-07
hsa-miR-1276	8356	HIST1H3C	0.63	3.23E-12	5.46E-07
hsa-miR-1276	8356	HIST1H3B	0.63	3.23E-12	5.46E-07
hsa-miR-1276	8356	HIST1H3A	0.63	3.23E-12	5.46E-07
hsa-miR-1276	8356	HIST1H3F	0.63	3.23E-12	5.46E-07
hsa-miR-892a	11096	ADAMTS5	0.63	3.14E-12	5.31E-07
hsa-miR-206	8573	CASK	0.63	3.12E-12	5.27E-07
hsa-miR-206	8440	NCK2	0.63	2.97E-12	5.03E-07
hsa-miR-301b	23060	ZNF609	0.63	2.87E-12	4.85E-07
hsa-miR-216b	9730	VPRBP	0.64	2.29E-12	3.87E-07
hsa-miR-217	3181	HNRNPA2B1	0.64	2.13E-12	3.61E-07
hsa-miR-206	8202	NCOA3	0.64	2.07E-12	3.49E-07
hsa-miR-301b	91833	WDR20	0.64	2.00E-12	3.37E-07
hsa-miR-216b	85458	DIXDC1	0.64	1.97E-12	3.33E-07
hsa-miR-217	11278	KLF12	0.64	1.83E-12	3.09E-07
hsa-miR-216b	55719	FAM178A	0.64	1.70E-12	2.88E-07
hsa-miR-206	6908	TBP	0.64	1.62E-12	2.73E-07
hsa-miR-206	10521	DDX17	0.64	1.57E-12	2.66E-07

hsa-miR-206	9782	MATR3	0.64	1.44E-12	2.44E-07
hsa-miR-216b	254225	RNF169	0.64	1.44E-12	2.43E-07
hsa-miR-206	55904	MLL5	0.64	1.34E-12	2.27E-07
hsa-miR-206	79684	MSANTD2	0.64	1.32E-12	2.23E-07
hsa-miR-206	8161	COIL	0.64	1.30E-12	2.19E-07
hsa-miR-301b	694	BTG1	0.64	1.28E-12	2.17E-07
hsa-miR-301b	9334	B4GALT5	0.64	1.28E-12	2.16E-07
hsa-miR-216b	23598	PATZ1	0.64	1.20E-12	2.03E-07
hsa-miR-206	65124	SOWAHC	0.64	1.19E-12	2.01E-07
hsa-miR-217	7552	ZNF711	0.64	1.10E-12	1.86E-07
hsa-miR-206	3192	HNRNPU	0.64	1.05E-12	1.78E-07
hsa-miR-1252	284058	KANSL1	0.64	1.05E-12	1.77E-07
hsa-miR-206	1951	CELSR3	0.65	8.03E-13	1.36E-07
hsa-miR-206	8322	FZD4	0.65	6.63E-13	1.12E-07
hsa-miR-206	26523	AGO1	0.65	5.74E-13	9.70E-08
hsa-miR-206	121512	FGD4	0.65	5.63E-13	9.51E-08
hsa-miR-206	57223	SMEK2	0.65	5.26E-13	8.88E-08
hsa-miR-217	81669	CCNL2	0.65	4.68E-13	7.92E-08
hsa-miR-296-3p	3842	TNPO1	0.65	4.62E-13	7.80E-08
hsa-miR-206	55327	LIN7C	0.65	4.40E-13	7.43E-08
hsa-miR-206	84458	LCOR	0.65	3.91E-13	6.61E-08
hsa-miR-216b	23095	KIF1B	0.65	3.53E-13	5.97E-08
hsa-miR-206	23607	CD2AP	0.65	3.40E-13	5.75E-08
hsa-miR-217	6664	SOX11	0.66	3.32E-13	5.61E-08
hsa-miR-206	9019	MPZL1	0.66	3.18E-13	5.37E-08
hsa-miR-216b	22828	SCAF8	0.66	3.10E-13	5.23E-08
hsa-miR-219-1-3p	10658	CELF1	0.66	2.83E-13	4.78E-08
hsa-miR-206	55216	C11orf57	0.66	1.85E-13	3.12E-08
hsa-miR-216b	5725	PTBP1	0.66	1.51E-13	2.55E-08
hsa-miR-206	6598	SMARCB1	0.66	1.33E-13	2.24E-08
hsa-miR-301b	3614	IMPDH1	0.66	1.31E-13	2.22E-08
hsa-miR-449a	27086	FOXP1	0.66	1.21E-13	2.05E-08
hsa-miR-206	3720	JARID2	0.66	1.10E-13	1.86E-08
hsa-miR-206	84662	GLIS2	0.67	9.44E-14	1.60E-08
hsa-miR-216b	149420	PDIK1L	0.67	8.64E-14	1.46E-08
hsa-miR-206	1875	E2F5	0.67	7.34E-14	1.24E-08
hsa-miR-216b	84458	LCOR	0.67	6.65E-14	1.12E-08
hsa-miR-206	7716	VEZF1	0.67	6.19E-14	1.05E-08
hsa-miR-217	23331	TTC28	0.67	6.03E-14	1.02E-08
hsa-miR-206	51497	NELFCD	0.67	6.01E-14	1.02E-08
hsa-miR-206	54467	ANKIB1	0.67	5.63E-14	9.51E-09
hsa-miR-206	7705	ZNF146	0.67	5.48E-14	9.27E-09
hsa-miR-206	64324	NSD1	0.67	5.19E-14	8.78E-09
hsa-miR-216b	10644	IGF2BP2	0.67	5.07E-14	8.58E-09
hsa-miR-206	55023	PHIP	0.67	4.95E-14	8.37E-09
hsa-miR-217	64919	BCL11B	0.67	4.66E-14	7.88E-09
hsa-miR-206	84530	SRRM4	0.67	4.57E-14	7.73E-09
hsa-miR-206	4211	MEIS1	0.67	4.45E-14	7.52E-09
hsa-miR-206	152006	RNF38	0.67	4.27E-14	7.21E-09

hsa-miR-206	84295	PHF6	0.67	4.15E-14	7.02E-09
hsa-miR-206	2335	FN1	0.68	2.88E-14	4.86E-09
hsa-miR-206	5612	PRKRIR	0.68	2.71E-14	4.59E-09
hsa-miR-206	3150	HMGN1	0.68	2.66E-14	4.49E-09
hsa-miR-206	9057	SLC7A6	0.68	2.44E-14	4.12E-09
hsa-miR-216b	7976	FZD3	0.68	2.41E-14	4.08E-09
hsa-miR-219-1-3p	58500	ZNF250	0.68	2.14E-14	3.62E-09
hsa-miR-206	55632	G2E3	0.68	1.72E-14	2.90E-09
hsa-miR-219-1-3p	1947	EFNB1	0.68	1.55E-14	2.63E-09
hsa-miR-216b	11078	TRIOBP	0.68	1.50E-14	2.53E-09
hsa-miR-219-1-3p	23049	SMG1	0.68	1.37E-14	2.32E-09
hsa-miR-206	64919	BCL11B	0.68	1.25E-14	2.12E-09
hsa-miR-206	8880	FUBP1	0.68	1.08E-14	1.83E-09
hsa-miR-301b	8829	NRP1	0.69	6.97E-15	1.18E-09
hsa-miR-206	6422	SFRP1	0.69	6.07E-15	1.03E-09
hsa-miR-206	51447	IP6K2	0.69	4.39E-15	7.43E-10
hsa-miR-206	54329	GPR85	0.69	3.58E-15	6.05E-10
hsa-miR-216b	10613	ERLIN1	0.69	3.10E-15	5.24E-10
hsa-miR-206	5226	PGD	0.70	2.71E-15	4.58E-10
hsa-miR-219-1-3p	4800	NFYA	0.70	2.70E-15	4.57E-10
hsa-miR-206	4076	CAPRIN1	0.70	1.86E-15	3.14E-10
hsa-miR-216b	11278	KLF12	0.70	1.73E-15	2.93E-10
hsa-miR-206	23119	HIC2	0.70	1.58E-15	2.67E-10
hsa-miR-206	50804	MYEF2	0.70	1.52E-15	2.57E-10
hsa-miR-206	57585	CRAMP1L	0.70	1.51E-15	2.56E-10
hsa-miR-206	55609	ZNF280C	0.70	1.42E-15	2.40E-10
hsa-miR-206	8732	RNGTT	0.70	1.23E-15	2.08E-10
hsa-miR-206	653121	ZBTB8A	0.70	1.05E-15	1.77E-10
hsa-miR-216b	63973	NEUROG2	0.70	1.02E-15	1.73E-10
hsa-miR-206	23002	DAAM1	0.70	8.19E-16	1.38E-10
hsa-miR-206	26040	SETBP1	0.71	7.34E-16	1.24E-10
hsa-miR-216b	862	RUNX1T1	0.71	7.31E-16	1.24E-10
hsa-miR-206	80311	KLHL15	0.71	6.96E-16	1.18E-10
hsa-miR-216b	9589	WTAP	0.71	4.30E-16	7.28E-11
hsa-miR-216b	26053	AUTS2	0.71	4.28E-16	7.23E-11
hsa-miR-216b	694	BTG1	0.71	2.60E-16	4.40E-11
hsa-miR-206	196528	ARID2	0.72	1.73E-16	2.93E-11
hsa-miR-206	51320	MEX3C	0.72	1.55E-16	2.62E-11
hsa-miR-206	51444	RNF138	0.72	1.54E-16	2.61E-11
hsa-miR-216b	11168	PSIP1	0.72	1.38E-16	2.34E-11
hsa-miR-206	1488	CTBP2	0.72	1.11E-16	1.87E-11
hsa-miR-206	10020	GNE	0.72	1.05E-16	1.77E-11
hsa-miR-206	1948	EFNB2	0.72	8.70E-17	1.47E-11
hsa-miR-206	149420	PDIK1L	0.73	3.88E-17	6.56E-12
hsa-miR-216b	4800	NFYA	0.73	3.83E-17	6.47E-12
hsa-miR-206	3156	HMGCR	0.73	3.66E-17	6.20E-12
hsa-miR-206	3842	TNPO1	0.73	2.02E-17	3.41E-12
hsa-miR-206	65267	WNK3	0.74	9.55E-18	1.62E-12
hsa-miR-216b	51082	POLR1D	0.74	6.17E-18	1.04E-12

hsa-miR-206	5150	PDE7A	0.74	2.89E-18	4.88E-13
hsa-miR-206	10773	ZBTB6	0.75	1.33E-18	2.26E-13
hsa-miR-206	54874	FNBP1L	0.75	5.70E-19	9.65E-14
hsa-miR-206	78997	GDAP1L1	0.76	3.75E-19	6.34E-14
hsa-miR-206	57188	ADAMTSL3	0.76	3.37E-19	5.70E-14
hsa-miR-206	22822	PHLDA1	0.76	1.49E-19	2.52E-14
hsa-miR-206	84668	FAM126A	0.76	1.05E-19	1.78E-14
hsa-miR-206	60468	BACH2	0.76	7.67E-20	1.30E-14
hsa-miR-206	8829	NRP1	0.77	4.07E-20	6.89E-15
hsa-miR-206	6591	SNAI2	0.78	1.05E-20	1.78E-15
hsa-miR-206	84870	RSPO3	0.78	8.60E-21	1.46E-15
hsa-miR-216b	1746	DLX2	0.78	3.89E-21	6.57E-16

Supplementary Table 3. Permutation test on the correlation between miRNA and mRNA expression in the DLPFC

	% total miRNA–target mRNA pairs (positively correlated)	% total miRNA–target mRNA pairs (negatively correlated)
Original analysis	4.00E-01	2.95E-01
Permutation 1	8.28E-03	0
Permutation 2	2.36E-03	2.95E-03
Permutation 3	2.96E-03	2.36E-03
Permutation 4	3.55E-03	5.90E-04
Permutation 5	3.55E-03	2.95E-03
Permutation 6	1.77E-03	4.72E-03
Permutation 7	0	1.18E-03
Permutation 8	2.36E-03	1.77E-03
Permutation 9	1.18E-03	1.77E-03
Permutation 10	3.55E-03	3.55E-03

Supplementary Table 4. Demographic characteristics of brain donors used to compare miRNA expression between schizophrenia and control subjects

AA: African American; CAUC: Caucasian

Brain ID	Diagnosis	Age (year)	Gender	Race	Post-mortem Interval (hour)	pH
890	Control	18.2	M	AA	36.5	6.7
1407	Control	18.3	M	AA	14	6.37
1143	Control	18.5	M	AA	11.5	6.62
1861	Control	18.7	M	CAUC	9	6.12
1865	Control	18.8	F	CAUC	15	6.67
885	Control	19.9	M	AA	21.5	6.93
1563	Control	21.3	F	AA	18	6.81
1306	Control	21.5	M	AA	15	6.49
1558	Control	23.4	F	CAUC	17.5	6.06
1518	Control	24.0	M	CAUC	20.5	6.98
1340	Control	26.2	F	AA	42	6.92
1604	Control	27.2	M	AA	14	6.37
1204	Control	27.5	M	CAUC	19.5	6.59
1389	Control	28.4	M	CAUC	19	6.29
1469	Control	28.6	M	CAUC	7.5	6.85
1410	Control	29.8	M	AA	29.5	6.91
1556	Control	30.0	F	AA	37.5	6.85
1761	Control	30.0	M	CAUC	22	6.08
1405	Control	31.5	F	AA	33.5	6.91
1142	Control	33.0	M	AA	43	6.62
1446	Control	34.5	M	CAUC	29.5	6.71
1191	Control	36.6	M	AA	36.5	6.55
1408	Control	37.2	M	AA	13	6.37
1324	Control	38.6	F	AA	38.5	6.58
1409	Control	40.6	F	CAUC	28.5	6.1
1674	Control	42.8	M	CAUC	24.5	6.7
1105	Control	44.2	F	AA	89.5	6.22
1473	Control	44.5	M	AA	29	6.77
1517	Control	44.8	F	AA	62.5	6.93
1644	Control	45.3	M	AA	21.5	6.61
1179	Control	45.4	F	AA	43	6.43
1432	Control	45.7	M	CAUC	43.5	6.26
1325	Control	45.8	F	AA	25	6.74
948	Control	45.9	M	AA	38.5	6.66
1525	Control	46.1	F	AA	13	7.1
1271	Control	46.6	M	CAUC	16	6.93
1532	Control	46.6	M	CAUC	68	6.59
1750	Control	46.9	M	CAUC	26.5	6.16
1649	Control	47.0	M	CAUC	67.5	6.84
1164	Control	48.1	F	AA	16	6.62
1017	Control	48.5	F	AA	23.5	6.25
1122	Control	48.6	M	AA	23	6.67
1092	Control	49.0	M	CAUC	18	6.13
1753	Control	49.0	F	CAUC	36	6.47
1463	Control	49.6	M	CAUC	18.5	6.57
1193	Control	49.8	M	AA	51	6.83
1039	Control	51.5	F	AA	24	6.79
1249	Control	51.8	F	AA	28	6.13

1113	Control	51.9	F	CAUC	51.5	6.53
859	Control	52.2	M	AA	19.5	6.52
910	Control	53.0	F	AA	10	6.66
1314	Control	53.4	F	AA	50.5	6.5
946	Control	54.0	M	AA	32.5	6.76
1351	Control	54.0	M	CAUC	63	6.17
1539	Control	54.6	F	AA	53.5	6.98
1007	Control	57.1	M	AA	25	6.5
1034	Control	57.6	M	CAUC	24	6.29
1006	Control	57.9	M	AA	47.5	6.72
894	Control	58.0	F	AA	24	6.06
1305	Control	58.4	M	AA	50	5.96
1304	Control	58.6	M	CAUC	64	6.87
1069	Control	59.6	F	AA	37	6.64
1135	Control	60.4	M	AA	44	6.59
1297	Control	62.8	M	AA	37	6.79
1695	Control	63.2	M	CAUC	26	6.74
925	Control	66.7	F	CAUC	29.5	6.48
1756	Control	71.1	F	CAUC	23.5	6.16
1160	Control	73.3	M	AA	68.5	6.41
1448	Schizophrenia	18.6	M	AA	32	6.68
1016	Schizophrenia	20.2	M	CAUC	24.5	6.65
1455	Schizophrenia	21.6	M	AA	44	6.89
888	Schizophrenia	25.6	M	CAUC	43	6.8
1183	Schizophrenia	26.1	F	CAUC	29.5	6.72
1416	Schizophrenia	37.2	M	CAUC	21	6.76
978	Schizophrenia	38.7	M	AA	61	6.45
1178	Schizophrenia	40.4	F	AA	60	6.55
1025	Schizophrenia	41.6	F	AA	51	6.1
1497	Schizophrenia	44.0	M	CAUC	33.5	6.07
1139	Schizophrenia	44.9	F	CAUC	44	6.22
1399	Schizophrenia	45.0	F	AA	17.5	6.8
856	Schizophrenia	47.4	M	AA	168	6.25
1030	Schizophrenia	49.7	F	AA	14.5	6.41
1588	Schizophrenia	50.6	M	AA	46.5	6.04
1479	Schizophrenia	51.7	M	AA	72	6.64
1688	Schizophrenia	52.1	M	AA	51	6.4
1840	Schizophrenia	55.4	M	AA	33.5	6.45
1738	Schizophrenia	55.7	M	CAUC	58.5	6.49
1580	Schizophrenia	56.9	F	CAUC	15	6.37
1751	Schizophrenia	57.2	M	AA	30.5	6.16
1383	Schizophrenia	59.0	M	CAUC	13.5	6.75
873	Schizophrenia	59.5	F	AA	48	6.13
1538	Schizophrenia	59.9	F	AA	29	6.5
1700	Schizophrenia	60.0	M	AA	24	6.15
1298	Schizophrenia	60.2	M	AA	38	6.68
855	Schizophrenia	60.8	F	AA	19	6.41
963	Schizophrenia	62.7	F	AA	76	6.38
1021	Schizophrenia	63.2	M	AA	56.5	6.72
858	Schizophrenia	64.6	F	AA	58.5	6.09
1576	Schizophrenia	66.3	F	AA	32	6.19
1676	Schizophrenia	68.0	M	CAUC	45	6.18
1594	Schizophrenia	69.0	M	CAUC	13.5	6.27
1093	Schizophrenia	71.9	F	CAUC	40	6.28

Supplementary Table 5. Demographic characteristics of brain donors used for PCR

CAUC: Caucasian; AA: African American

Brain ID	Diagnosis	Age (year)	Gender	Race	Post-mortem Interval (hour)	pH
1876	Control	16.4	M	CAUC	16	6.57
1306	Control	21.5	M	AA	15	6.98
1518	Control	24.0	M	CAUC	20.5	6.29
1463	Control	49.6	M	CAUC	18.5	6.49
1034	Control	57.6	M	CAUC	24	6.68
1016	Schizo	20.2	M	CAUC	24.5	6.65
888	Schizo	25.6	M	CAUC	43	6.8
1479	Schizo	51.7	M	AA	72	6.64
1383	Schizo	59.0	M	CAUC	13.5	6.75
1594	Schizo	69.0	M	CAUC	13.5	6.27

Supplementary Table 6. Genes co-targeted by Group 6 miRNAs

Gene ID	HGNC symbol	# miRNA targeting sites	# sites targeted by Group 6 miRNAs	*p*-value	FDR
1605	DAG1	598	104	6.498E-15	9.11E-11
4582	MUC1	20	20	6.989E-14	4.90E-10
1740	DLG2	735	115	1.506E-13	7.03E-10
23043	TNIK	80	32	6.006E-13	2.10E-09
7165	TPD52L2	144	42	1.111E-12	3.11E-09
11143	KAT7	250	55	7.098E-12	1.66E-08
115677	NOSTRIN	16	16	2.232E-11	4.47E-08
10313	RTN3	100	32	7.16E-11	1.25E-07
6560	SLC12A4	60	25	9.694E-11	1.51E-07
775	CACNA1C	2990	299	6.186E-09	8.67E-06
5076	PAX2	280	50	2.638E-08	3.07E-05
23154	NCDN	141	33	2.802E-08	3.07E-05
3612	IMPA1	27	15	2.847E-08	3.07E-05
286	ANK1	568	80	3.966E-08	3.97E-05
4303	FOXO4	138	32	5.412E-08	5.06E-05
8496	PPFIBP1	88	24	1.979E-07	1.73E-04
4221	MEN1	28	14	2.224E-07	1.83E-04
54840	APTX	40	16	3.436E-07	2.67E-04
3983	ABLIM1	92	24	3.933E-07	2.90E-04
6432	SRSF7	126	28	7.388E-07	5.14E-04
7555	CNBP	534	72	7.709E-07	5.14E-04
1500	CTNND1	1958	198	9.955E-07	6.34E-04
2011	MARK2	520	70	1.14E-06	6.95E-04
55634	KRBOX4	48	16	2.446E-06	1.43E-03
4133	MAP2	204	36	2.708E-06	1.43E-03
4010	LMX1B	153	30	2.871E-06	1.43E-03
23089	PEG10	684	84	2.948E-06	1.43E-03
652	BMP4	12	9	2.967E-06	1.43E-03
2922	GRP	12	9	2.967E-06	1.43E-03
3267	AGFG1	600	76	3.133E-06	1.46E-03
7534	YWHAZ	690	84	3.976E-06	1.80E-03
2104	ESRRG	651	80	4.86E-06	2.13E-03
60592	SCOC	176	32	5.483E-06	2.33E-03
255743	NPNT	250	40	6.501E-06	2.68E-03
1608	DGKG	138	27	9.053E-06	3.62E-03
9619	ABCG1	30	12	9.961E-06	3.88E-03
1996	ELAVL4	210	35	1.101E-05	4.17E-03
10335	MRVI1	276	42	1.15E-05	4.24E-03
2334	AFF2	522	66	1.387E-05	4.98E-03
23332	CLASP1	260	40	1.463E-05	5.00E-03
80821	DDHD1	429	57	1.463E-05	5.00E-03
29993	PACSIN1	88	20	2.015E-05	6.72E-03

10742	RAI2	60	16	2.572E-05	8.38E-03
23759	PPIL2	18	9	3.307E-05	1.02E-02
177	AGER	18	9	3.307E-05	1.02E-02
23034	SAMD4A	381	51	3.334E-05	1.02E-02
286144	TRIQK	364	49	4.127E-05	1.20E-02
3614	IMPDH1	294	42	4.271E-05	1.20E-02
80036	TRPM3	49	14	4.298E-05	1.20E-02
6253	RTN2	36	12	4.353E-05	1.20E-02
79567	FAM65A	36	12	4.353E-05	1.20E-02
64783	RBM15	14	8	4.456E-05	1.20E-02
5063	PAK3	6	6	4.953E-05	1.27E-02
10886	NPFFR2	6	6	4.953E-05	1.27E-02
51088	KLHL5	200	32	5.116E-05	1.27E-02
4140	MARK3	95	20	5.149E-05	1.27E-02
9055	PRC1	57	15	5.177E-05	1.27E-02
79980	DSN1	25	10	5.458E-05	1.32E-02
8082	SSPN	112	22	5.542E-05	1.32E-02
440574	MINOS1	156	27	5.965E-05	1.39E-02
6938	TCF12	540	65	6.036E-05	1.39E-02
25976	TIPARP	105	21	6.46E-05	1.46E-02
1788	DNMT3A	81	18	6.58E-05	1.46E-02
3664	IRF6	66	16	6.83E-05	1.50E-02
23162	MAPK8IP3	82	18	7.54E-05	1.62E-02
140578	CHODL	132	24	7.64E-05	1.62E-02
79796	ALG9	324	44	8.19E-05	1.71E-02
64218	SEMA4A	16	8	9.11E-05	1.88E-02
1240	CMKLR1	68	16	9.24E-05	1.88E-02
57604	KIAA1456	84	18	9.83E-05	1.97E-02
1908	EDN3	40	12	1.01E-04	2.00E-02
1837	DTNA	465	57	1.07E-04	2.07E-02
23468	CBX5	771	84	1.36E-04	2.60E-02
6470	SHMT1	42	12	1.49E-04	2.82E-02
57190	SEPN1	72	16	1.64E-04	3.03E-02
29789	OLA1	158	26	1.65E-04	3.03E-02
6660	SOX5	315	42	1.67E-04	3.03E-02
2318	FLNC	30	10	1.87E-04	3.30E-02
4776	NFATC4	81	17	1.88E-04	3.30E-02
1780	DYNC1I1	24	9	1.91E-04	3.30E-02
79184	BRCC3	24	9	1.91E-04	3.30E-02
55769	ZNF83	90	18	2.08E-04	3.56E-02
23552	CDK20	5	5	2.22E-04	3.69E-02
3384	ICAM2	5	5	2.22E-04	3.69E-02
8856	NR1I2	9	6	2.26E-04	3.69E-02
79613	TANGO6	9	6	2.26E-04	3.69E-02
144165	PRICKLE1	108	20	2.35E-04	3.78E-02
84630	TTBK1	84	17	2.73E-04	4.35E-02
57446	NDRG3	76	16	2.78E-04	4.38E-02

2309	FOXO3	242	34	2.82E-04	4.40E-02

Supplementary Table 7. Genes co-targeted by Group 8 miRNAs

Gene ID	HGNC symbol	# miRNA targeting sites	# sites targeted by Group 8 miRNAs	*p*-value	FDR
9643	MORF4L2	752	160	1.09E-41	1.53E-37
8913	CACNA1G	100	42	8.52E-21	5.97E-17
5144	PDE4D	1746	189	8.92E-17	4.17E-13
3084	NRG1	334	63	2.71E-15	9.51E-12
10018	BCL2L11	2236	208	2.32E-12	5.98E-09
6778	STAT6	65	25	2.56E-12	5.98E-09
7323	UBE2D3	711	90	6.44E-12	1.29E-08
10801	9-Sep	161	35	1.32E-10	2.30E-07
25850	ZNF345	90	25	7.96E-10	1.24E-06
51174	TUBD1	48	18	3.93E-09	5.50E-06
4287	ATXN3	948	96	4.74E-08	6.04E-05
9802	DAZAP2	281	40	2.75E-07	3.21E-04
117854	TRIM6	8	8	4.31E-07	4.65E-04
2898	GRIK2	248	36	6.95E-07	6.95E-04
132864	CPEB2	1614	138	7.76E-07	7.24E-04
8904	CPNE1	30	12	8.78E-07	7.24E-04
56650	CLDND1	30	12	8.78E-07	7.24E-04
7050	TGIF1	128	24	1.01E-06	7.86E-04
30817	EMR2	7	7	2.36E-06	1.67E-03
22998	LIMCH1	430	50	2.39E-06	1.67E-03
79109	MAPKAP1	22	10	2.91E-06	1.85E-03
115201	ATG4A	22	10	2.91E-06	1.85E-03
9527	GOSR1	201	30	3.41E-06	2.08E-03
11142	PKIG	18	9	5.01E-06	2.81E-03
9936	CD302	18	9	5.01E-06	2.81E-03
135112	NCOA7	498	54	6.26E-06	3.37E-03
84268	RPAIN	14	8	8.10E-06	4.21E-03
200150	PLD5	63	15	9.15E-06	4.58E-03
7157	TP53	119	21	1.05E-05	5.09E-03
5562	PRKAA1	154	24	1.64E-05	7.68E-03
10804	GJB6	16	8	1.70E-05	7.68E-03
132	ADK	76	16	1.77E-05	7.76E-03
9581	PREPL	126	21	2.25E-05	9.55E-03
9584	RBM39	127	21	2.49E-05	9.83E-03
2554	GABRA1	371	42	2.54E-05	9.83E-03
1727	CYB5R3	30	10	2.62E-05	9.83E-03
1508	CTSB	70	15	2.68E-05	9.83E-03
4286	MITF	462	49	2.72E-05	9.83E-03
10468	FST	45	12	2.74E-05	9.83E-03
9685	CLINT1	99	18	3.09E-05	1.08E-02
1602	DACH1	195	27	3.34E-05	1.13E-02
813	CALU	696	66	3.39E-05	1.13E-02

169026	SLC30A8	230	30	3.48E-05	1.14E-02
8450	CUL4B	48	12	4.69E-05	1.43E-02
79657	RPAP3	48	12	4.69E-05	1.43E-02
114907	FBXO32	270	33	4.70E-05	1.43E-02
79723	SUV39H2	75	15	5.36E-05	1.60E-02
5521	PPP2R2B	14	7	5.80E-05	1.69E-02
6322	SCML1	20	8	5.97E-05	1.71E-02
10733	PLK4	9	6	6.16E-05	1.73E-02
55861	DBNDD2	27	9	6.61E-05	1.79E-02
11030	RBPMS	116	19	6.63E-05	1.79E-02
759	CA1	5	5	7.26E-05	1.89E-02
25937	WWTR1	138	21	7.28E-05	1.89E-02
518	ATP5G3	28	9	8.34E-05	2.12E-02
84969	TOX2	52	12	9.09E-05	2.28E-02
5000	ORC4	246	30	1.04E-04	2.57E-02
10492	SYNCRIP	784	70	1.09E-04	2.64E-02
2332	FMR1	440	45	1.20E-04	2.85E-02
463	ZFHX3	298	34	1.23E-04	2.85E-02
26065	LSM14A	54	12	1.24E-04	2.85E-02
28985	MCTS1	180	24	1.48E-04	3.33E-02
2104	ESRRG	651	60	1.50E-04	3.33E-02
6863	TAC1	24	8	1.68E-04	3.68E-02
2070	EYA4	255	30	1.84E-04	3.97E-02
10768	AHCYL1	195	25	1.89E-04	4.00E-02
57818	G6PC2	12	6	2.00E-04	4.00E-02
100526664	LY75-CD302	12	6	2.00E-04	4.00E-02
200909	HTR3D	12	6	2.00E-04	4.00E-02
100526664	LY75	12	6	2.00E-04	4.00E-02
84970	C1orf94	12	6	2.00E-04	4.00E-02
54933	RHBDL2	7	5	2.09E-04	4.13E-02
10933	MORF4L1	58	12	2.21E-04	4.31E-02
672	BRCA1	140	20	2.27E-04	4.33E-02
154661	RUNDC3B	87	15	2.29E-04	4.33E-02
6935	ZEB1	750	66	2.42E-04	4.52E-02
29966	STRN3	164	22	2.53E-04	4.66E-02
3600	IL15	26	8	2.64E-04	4.81E-02

Supplementary Table 8. Signal transuction networks enriched by the target genes of Group 6 miRNAs

Network	Network id	P-value	Adjusted p-value	# Genes (observed)	# Genes (expected)	# Genes (total)	List of observed genes	Gene ids
GLYCOGEN SYNTHASE KINASE	PW_GSK_HOMO_SAPIENS	0	0	9	2.10	257	MUC1, MARK2, YWHAZ, OLA1, FOXO4, MAP2, FOXO3, PACSIN1, CDK20	4582, 2011, 7534, 29789, 4303, 4133, 2309, 29993, 23552
14 3 3 PROTEIN	PW_14 3 3_HOMO_SAPIENS	0	0.001	7	1.35	165	MARK3, MARK2, YWHAZ, FOXO4, MAP2, FOXO3, SAMD4A	4140, 2011, 7534, 4303, 4133, 2309, 23034
MICROTUBULE AFFINITY REGULATING KINASE	PW_MARK_HOMO_SAPIENS	0.001	0	4	0.44	54	TTBK1, MARK3, MARK2, MAP2	84630, 4140, 2011, 4133
DEVELOPMENTAL	PW_DEVELOPMENTAL_HOMO_SAPIENS	0.001	0	9	2.60	319	BMP4, TCF12, PAX2, NOSTRIN, IRF6, ABLIM1, CBX5, RBM15, LMX1B	652, 6938, 5076, 115677, 3664, 3983, 23468, 64783, 4010
NUCLEAR RECEPTOR SUBFAMILY 4, GROUP A, MEMBER 2	PW_NR4A2_HOMO_SAPIENS	0.004	0.005	3	0.31	38	LMX1B, MAP2, SOX5	4010, 4133, 6660
GLIAL CELL LINE DERIVED NEUROTROPHIC FACTOR	PW_GDNF_HOMO_SAPIENS	0.005	0.002	4	0.69	84	BMP4, PAX2, EDN3, NPNT	652, 5076, 1908, 255743
SERINE/THREONINE KINASE 11 | LKB1	PW_STK11_HOMO_SAPIENS	0.005	0.005	4	0.69	85	MARK3, MARK2, YWHAZ, FOXO3	4140, 2011, 7534, 2309
NITRIC OXIDE	PW_NITRIC_OXIDE_HOMO_SAPIENS	0.007	0.005	5	1.19	146	NOSTRIN, EDN3, SLC12A4, MRVI1, MAP2	115677, 1908, 6560, 10335, 4133
SNAIL FAMILY ZINC FINGER 1	PW_SNAIL_HOMO_SAPIENS	0.010	0.011	5	1.31	160	PEG10, YWHAZ, OLA1, DNMT3A, TPD52L2	23089, 7534, 29789, 1788, 7165

Supplementary Table 9. Molecular functions enriched by the target genes of Group 6 miRNAs

GO-Term	GO-Term id	P-value	Adjusted p-value	# Genes (observed)	# Genes (expected)	# Genes (total)	List of observed genes	Gene ids
dystroglycan binding	GO:0002162	0	0	3	0.05	7	DAG1, CLASP1, MAP2	1605, 23332, 4133
regulatory region DNA binding	GO:0000975	0.001	0	13	4.98	709	LMX1B, NR1I2, NFATC4, KAT7, CNBP, ESRRG, TCF12, IRF6, MEN1, TIPARP, FOXO3, MUC1, PAX2	4010, 8856, 4776, 11143, 7555, 2104, 6938, 3664, 4221, 25976, 2309, 4582, 5076
regulatory region nucleic acid binding	GO:0001067	0.001	0	13	4.99	710	LMX1B, NR1I2, NFATC4, KAT7, CNBP, ESRRG, TCF12, IRF6, MEN1, TIPARP, FOXO3, MUC1, PAX2	4010, 8856, 4776, 11143, 7555, 2104, 6938, 3664, 4221, 25976, 2309, 4582, 5076

protein binding	GO:0005515	0.002	0.002	73	60.92	8667	PACSIN1, SELENON, MARK3, NOSTRIN, ICAM2, GRP, DSN1, CBX5, YWHAZ, PEG10, AGFG1, LMX1B, NCDN, PAK3, SEMA4A, PRICKLE1, NPNT, NR1I2, MAPK8IP3, CMKLR1, NFATC4, KAT7, CNBP, SOX5, SCOC, AGER, TNIK, PPIL2, CACNA1C, DAG1, MARK2, ESRRG, SAMD4A, IMPA1, ZNF83, SHMT1, DTNA, TCF12, RBM15, RIPOR1, CLASP1, IRF6, RTN2, RTN3, MEN1, MRVI1, FLNC, FOXO3, DNMT3A, MAP2, EDN3, ANK1, NPFFR2, DYNC1I1, FOXO4, BRCC3, MUC1, ABCG1, RAI2, CTNND1, KLHL5, DLG2, SRSF7, SLC12A4, PRC1, PAX2, OLA1, ABLIM1, PPFIBP1, APTX, MINOS1, BMP4, CDK20	29993, 57190, 4140, 115677, 3384, 2922, 79980, 23468, 7534, 23089, 3267, 4010, 23154, 5063, 64218, 144165, 255743, 8856, 23162, 1240, 4776, 11143, 7555, 6660, 60592, 177, 23043, 23759, 775, 1605, 2011, 2104, 23034, 3612, 55769, 6470, 1837, 6938, 64783, 79567, 23332, 3664, 6253, 10313, 4221, 10335, 2318, 2309, 1788, 4133, 1908, 286, 10886, 1780, 4303, 79184, 4582, 9619, 10742, 1500, 51088, 1740, 6432, 6560, 9055, 5076, 29789, 3983, 8496, 54840, 440574, 652, 23552

binding	GO:0005488	0.003	0.005	84	76.11	10828	PACSIN1, SELENON, MARK3, NOSTRIN, ICAM2, TPD52L2, GRP, DSN1, CBX5, YWHAZ, PEG10, AGFG1, LMX1B, NCDN, CHODL, PAK3, SEMA4A, PRICKLE1, NPNT, NR1I2, MAPK8IP3, CMKLR1, NFATC4, KAT7, CNBP, DDHD1, DGKG, SOX5, SCOC, AGER, TNIK, PPIL2, IMPDH1, CACNA1C, DAG1, MARK2, ESRRG, SAMD4A, IMPA1, ZNF83, ELAVL4, SHMT1, DTNA, TCF12, RBM15, RIPOR1, CLASP1, IRF6, RTN2, KIAA1456, RTN3, MEN1, MRVI1, FLNC, TIPARP, FOXO3, DNMT3A, MAP2, EDN3, ANK1, NPFFR2, DYNC1I1, FOXO4, BRCC3, MUC1, KRBOX4, ABCG1, RAI2, CTNND1, KLHL5, AFF2, DLG2, SRSF7, SLC12A4, PRC1, PAX2, OLA1, ABLIM1, PPFIBP1, TTBK1, APTX, MINOS1, BMP4, CDK20	29993, 57190, 4140, 115677, 3384, 7165, 2922, 79980, 23468, 7534, 23089, 3267, 4010, 23154, 140578, 5063, 64218, 144165, 255743, 8856, 23162, 1240, 4776, 11143, 7555, 80821, 1608, 6660, 60592, 177, 23043, 23759, 3614, 775, 1605, 2011, 2104, 23034, 3612, 55769, 1996, 6470, 1837, 6938, 64783, 79567, 23332, 3664, 6253, 57604, 10313, 4221, 10335, 2318, 25976, 2309, 1788, 4133, 1908, 286, 10886, 1780, 4303, 79184, 4582, 55634, 9619, 10742, 1500, 51088, 2334, 1740, 6432, 6560, 9055, 5076, 29789, 3983, 8496, 84630, 54840, 440574, 652, 23552

nucleic acid binding	GO:0003676	0.004	0.006	33	21.31	3032	NOSTRIN, TPD52L2, YWHAZ, PEG10, AGFG1, LMX1B, NR1I2, NFATC4, KAT7, CNBP, SOX5, IMPDH1, MARK2, ESRRG, SAMD4A, ZNF83, ELAVL4, SHMT1, TCF12, RBM15, IRF6, KIAA1456, MEN1, TIPARP, FOXO3, DNMT3A, FOXO4, MUC1, KRBOX4, AFF2, SRSF7, PAX2, APTX	115677, 7165, 7534, 23089, 3267, 4010, 8856, 4776, 11143, 7555, 6660, 3614, 2011, 2104, 23034, 55769, 1996, 6470, 6938, 64783, 3664, 57604, 4221, 25976, 2309, 1788, 4303, 4582, 55634, 2334, 6432, 5076, 54840
transcription regulatory region DNA binding	GO:0044212	0.004	0.005	12	4.97	707	LMX1B, NR1I2, NFATC4, KAT7, CNBP, ESRRG, TCF12, MEN1, TIPARP, FOXO3, MUC1, PAX2	4010, 8856, 4776, 11143, 7555, 2104, 6938, 4221, 25976, 2309, 4582, 5076
cytoskeletal protein binding	GO:0008092	0.005	0.003	12	5.14	732	PACSIN1, MAPK8IP3, CACNA1C, DAG1, CLASP1, FLNC, MAP2, ANK1, DYNC1I1, KLHL5, PRC1, ABLIM1	29993, 23162, 775, 1605, 23332, 2318, 4133, 286, 1780, 51088, 9055, 3983
double-stranded DNA binding	GO:0003690	0.006	0.006	11	4.61	656	LMX1B, NR1I2, NFATC4, KAT7, CNBP, ESRRG, TCF12, MEN1, MUC1, PAX2, APTX	4010, 8856, 4776, 11143, 7555, 2104, 6938, 4221, 4582, 5076, 54840
translation repressor activity	GO:0030371	0.010	0.016	2	0.15	22	SAMD4A, SHMT1	23034, 6470

Supplementary Table 10. Biological processes enriched by the target genes of Group 6 miRNAs

GO-Term	GO-Term id	P-value	Adjusted p-value	# Genes (observed)	# Genes (expected)	# Genes (total)	List of observed genes	Gene ids
regulation of cell differentiation	GO:0045595	0	0	23	9.22	1324	MAP2, EDN3, FOXO3, FOXO4, CLASP1, RBM15, MEN1, BMP4, ABCG1, PAX2, CHODL, PACSIN1, DAG1, MARK2, TNIK, TCF12, NPNT, PRICKLE1, PAK3, AGER, SOX5, CMKLR1, NFATC4	4133, 1908, 2309, 4303, 23332, 64783, 4221, 652, 9619, 5076, 140578, 29993, 1605, 2011, 23043, 6938, 255743, 144165, 5063, 177, 6660, 1240, 4776
response to water-immersion restraint stress	GO:1990785	0	0	2	0.02	3	FOXO3, FOXO4	2309, 4303
neuron differentiation	GO:0030182	0	0	19	7.88	1131	MAP2, EDN3, DNMT3A, FOXO3, BMP4, PAX2, NCDN, CHODL, LMX1B, PACSIN1, DAG1, MARK2, TNIK, TCF12, SEMA4A, PAK3, AGER, DGKG, NFATC4	4133, 1908, 1788, 2309, 652, 5076, 23154, 140578, 4010, 29993, 1605, 2011, 23043, 6938, 64218, 5063, 177, 1608, 4776
smooth muscle cell differentiation	GO:0051145	0	0	4	0.31	44	FOXO4, BMP4, NPNT, NFATC4	4303, 652, 255743, 4776
generation of neurons	GO:0048699	0	0	20	8.61	1236	MAP2, EDN3, DNMT3A, FOXO3, BMP4, PAX2, NCDN, CHODL, LMX1B, PACSIN1, DAG1, MARK2, TNIK, TCF12, SEMA4A, PAK3, AGER, SOX5, DGKG, NFATC4	4133, 1908, 1788, 2309, 652, 5076, 23154, 140578, 4010, 29993, 1605, 2011, 23043, 6938, 64218, 5063, 177, 6660, 1608, 4776

branching morphogenesis of an epithelial tube	GO:0048754	0	0	6	0.97	139	RBM15, BMP4, PAX2, DAG1, NPNT, NFATC4	64783, 652, 5076, 1605, 255743, 4776
regulation of basement membrane assembly involved in embryonic body morphogenesis	GO:1904259	0	0	2	0.03	5	CLASP1, DAG1	23332, 1605
basement membrane assembly involved in embryonic body morphogenesis	GO:2001197	0	0	2	0.03	5	CLASP1, DAG1	23332, 1605
regulation of basement membrane organization	GO:0110011	0	0	2	0.03	5	CLASP1, DAG1	23332, 1605
positive regulation of basement membrane assembly involved in embryonic body morphogenesis	GO:1904261	0	0	2	0.03	5	CLASP1, DAG1	23332, 1605
nephric duct formation	GO:0072179	0	0	2	0.03	5	BMP4, PAX2	652, 5076

nervous system development	GO:0007399	0	0	26	13.60	1952	MAP2, EDN3, DNMT3A, FOXO3, MEN1, TTBK1, BMP4, DLG2, AFF2, CTNND1, PAX2, NCDN, CHODL, LMX1B, PACSIN1, DAG1, MARK2, TNIK, TCF12, SEMA4A, PRICKLE1, PAK3, AGER, SOX5, DGKG, NFATC4	4133, 1908, 1788, 2309, 4221, 84630, 652, 1740, 2334, 1500, 5076, 23154, 140578, 4010, 29993, 1605, 2011, 23043, 6938, 64218, 144165, 5063, 177, 6660, 1608, 4776
regulation of developmental process	GO:0050793	0	0	26	13.61	1954	MAP2, EDN3, FOXO3, FOXO4, CLASP1, RBM15, MEN1, TTBK1, BMP4, ABCG1, PAX2, CHODL, PACSIN1, DAG1, MARK2, TNIK, TCF12, NPNT, SEMA4A, PRICKLE1, PAK3, AGER, SOX5, DDHD1, CMKLR1, NFATC4	4133, 1908, 2309, 4303, 23332, 64783, 4221, 84630, 652, 9619, 5076, 140578, 29993, 1605, 2011, 23043, 6938, 255743, 64218, 144165, 5063, 177, 6660, 80821, 1240, 4776
neurogenesis	GO:0022008	0	0	20	9.17	1317	MAP2, EDN3, DNMT3A, FOXO3, BMP4, PAX2, NCDN, CHODL, LMX1B, PACSIN1, DAG1, MARK2, TNIK, TCF12, SEMA4A, PAK3, AGER, SOX5, DGKG, NFATC4	4133, 1908, 1788, 2309, 652, 5076, 23154, 140578, 4010, 29993, 1605, 2011, 23043, 6938, 64218, 5063, 177, 6660, 1608, 4776
positive regulation of histone H4 acetylation	GO:0090240	0	0	2	0.04	6	MUC1, KAT7	4582, 11143
establishment or maintenance of cell polarity	GO:0007163	0	0	6	1.12	161	ANK1, MAP2, CLASP1, DLG2, MARK3, MARK2	286, 4133, 23332, 1740, 4140, 2011

morphogenesis of a branching epithelium	GO:0061138	0	0	6	1.18	170	RBM15, BMP4, PAX2, DAG1, NPNT, NFATC4	64783, 652, 5076, 1605, 255743, 4776
cell differentiation	GO:0030154	0	0	35	21.85	3137	MAP2, EDN3, DNMT3A, FOXO3, TIPARP, FLNC, FOXO4, IRF6, CLASP1, RBM15, NDRG3, MEN1, BMP4, ABCG1, PAX2, NCDN, CHODL, PEG10, AGFG1, LMX1B, PACSIN1, SELENON, DAG1, MARK2, TNIK, TCF12, NPNT, SEMA4A, PRICKLE1, PAK3, AGER, SOX5, DGKG, CMKLR1, NFATC4	4133, 1908, 1788, 2309, 25976, 2318, 4303, 3664, 23332, 64783, 57446, 4221, 652, 9619, 5076, 23154, 140578, 23089, 3267, 4010, 29993, 57190, 1605, 2011, 23043, 6938, 255743, 64218, 144165, 5063, 177, 6660, 1608, 1240, 4776
establishment of Golgi localization	GO:0051683	0	0	2	0.06	8	RIPOR1, YWHAZ	79567, 7534
metanephric collecting duct	GO:0072205	0	0	2	0.06	8	BMP4, PAX2	652, 5076
basement membrane assembly	GO:0070831	0	0	2	0.06	8	CLASP1, DAG1	23332, 1605
mesenchymal cell differentiation involved in renal system development	GO:2001012	0	0	2	0.06	8	BMP4, PAX2	652, 5076

mesenchymal cell differentiation involved in kidney development	GO:0072161	0	0	2	0.06	8	BMP4, PAX2	652, 5076
negative regulation of metanephros development	GO:0072217	0	0	2	0.06	8	BMP4, PAX2	652, 5076
cellular developmental process	GO:0048869	0	0	36	22.84	3279	MAP2, EDN3, DNMT3A, FOXO3, TIPARP, FLNC, FOXO4, IRF6, CLASP1, RBM15, NDRG3, MEN1, TTBK1, BMP4, ABCG1, PAX2, NCDN, CHODL, PEG10, AGFG1, LMX1B, PACSIN1, SELENON, DAG1, MARK2, TNIK, TCF12, NPNT, SEMA4A, PRICKLE1, PAK3, AGER, SOX5, DGKG, CMKLR1, NFATC4	4133, 1908, 1788, 2309, 25976, 2318, 4303, 3664, 23332, 64783, 57446, 4221, 84630, 652, 9619, 5076, 23154, 140578, 23089, 3267, 4010, 29993, 57190, 1605, 2011, 23043, 6938, 255743, 64218, 144165, 5063, 177, 6660, 1608, 1240, 4776
establishment or maintenance of epithelial cell apical/basal polarity	GO:0045197	0	0	3	0.24	34	ANK1, DLG2, MARK2	286, 1740, 2011
pattern specification involved in kidney development	GO:0061004	0	0	2	0.06	9	BMP4, PAX2	652, 5076

positive regulation of extracellular matrix assembly	GO:1901203	0	0	2	0.06	9	CLASP1, DAG1	23332, 1605
renal system pattern specification	GO:0072048	0	0	2	0.06	9	BMP4, PAX2	652, 5076
morphogenesis of a branching structure	GO:0001763	0	0	6	1.27	183	RBM15, BMP4, PAX2, DAG1, NPNT, NFATC4	64783, 652, 5076, 1605, 255743, 4776
positive regulation of transcription from RNA polymerase II promoter	GO:0045944	0	0	15	6.53	937	FOXO3, MUC1, FOXO4, RBM15, MEN1, BMP4, PAX2, LMX1B, TCF12, ESRRG, NR1I2, NPNT, CNBP, NFATC4, KAT7	2309, 4582, 4303, 64783, 4221, 652, 5076, 4010, 6938, 2104, 8856, 255743, 7555, 4776, 11143
cell development	GO:0048468	0	0	22	11.66	1674	MAP2, EDN3, FOXO3, FLNC, IRF6, MEN1, BMP4, PAX2, NCDN, CHODL, AGFG1, PACSIN1, SELENON, DAG1, MARK2, TNIK, TCF12, SEMA4A, PAK3, AGER, DGKG, NFATC4	4133, 1908, 2309, 2318, 3664, 4221, 652, 5076, 23154, 140578, 3267, 29993, 57190, 1605, 2011, 23043, 6938, 64218, 5063, 177, 1608, 4776
regulation of anatomical structure morphogenesis	GO:0022603	0	0	14	5.91	848	MAP2, FOXO4, CLASP1, TTBK1, BMP4, PAX2, DAG1, MARK2, TNIK, SEMA4A, PRICKLE1, PAK3, DDHD1, NFATC4	4133, 4303, 23332, 84630, 652, 5076, 1605, 2011, 23043, 64218, 144165, 5063, 80821, 4776
positive regulation of cell differentiation	GO:0045597	0	0	13	5.25	754	EDN3, FOXO3, BMP4, PAX2, PACSIN1, DAG1, MARK2, TCF12, NPNT, PAK3, AGER, SOX5, CMKLR1	1908, 2309, 652, 5076, 29993, 1605, 2011, 6938, 255743, 5063, 177, 6660, 1240

embryonic body morphogenesis	GO:0010172	0	0	2	0.07	10	CLASP1, DAG1	23332, 1605
regulation of neuron differentiation	GO:0045664	0	0	10	3.48	500	MAP2, FOXO3, BMP4, CHODL, PACSIN1, MARK2, TNIK, TCF12, PAK3, NFATC4	4133, 2309, 652, 140578, 29993, 2011, 23043, 6938, 5063, 4776
establishment or maintenance of bipolar cell polarity	GO:0061245	0	0	3	0.27	39	ANK1, DLG2, MARK2	286, 1740, 2011
establishment or maintenance of apical/basal cell polarity	GO:0035088	0	0	3	0.27	39	ANK1, DLG2, MARK2	286, 1740, 2011
positive regulation of nitrogen compound metabolic process	GO:0051173	0	0	28	16.65	2391	EDN3, FOXO3, TIPARP, MUC1, BRCC3, FOXO4, IRF6, RBM15, MEN1, CDK20, BMP4, PAX2, LMX1B, DAG1, MARK2, TNIK, TCF12, SAMD4A, ESRRG, NR1I2, MAPK8IP3, NPNT, PRICKLE1, PAK3, AGER, CNBP, NFATC4, KAT7	1908, 2309, 25976, 4582, 79184, 4303, 3664, 64783, 4221, 23552, 652, 5076, 4010, 1605, 2011, 23043, 6938, 23034, 2104, 8856, 23162, 255743, 144165, 5063, 177, 7555, 4776, 11143
regulation of histone H4 acetylation	GO:0090239	0	0.005	2	0.08	11	MUC1, KAT7	4582, 11143
regulation of nervous system development	GO:0051960	0	0	12	4.81	691	MAP2, FOXO3, BMP4, PAX2, CHODL, PACSIN1, DAG1, MARK2, TNIK, TCF12, PAK3, NFATC4	4133, 2309, 652, 5076, 140578, 29993, 1605, 2011, 23043, 6938, 5063, 4776

positive regulation of metabolic process	GO:0009893	0	0.006	30	18.52	2659	EDN3, FOXO3, TIPARP, MUC1, BRCC3, FOXO4, IRF6, RBM15, MEN1, CDK20, BMP4, ABCG1, PAX2, LMX1B, DAG1, MARK2, TNIK, TCF12, SAMD4A, ESRRG, NR1I2, MAPK8IP3, NPNT, PRICKLE1, PAK3, SCOC, AGER, CNBP, NFATC4, KAT7	1908, 2309, 25976, 4582, 79184, 4303, 3664, 64783, 4221, 23552, 652, 9619, 5076, 4010, 1605, 2011, 23043, 6938, 23034, 2104, 8856, 23162, 255743, 144165, 5063, 60592, 177, 7555, 4776, 11143
collecting duct	GO:0072044	0	0	2	0.08	12	BMP4, PAX2	652, 5076
regulation of extracellular matrix assembly	GO:1901201	0	0	2	0.08	12	CLASP1, DAG1	23332, 1605
mesenchymal to epithelial transition involved in metanephros morphogenesis	GO:0003337	0	0	2	0.08	12	BMP4, PAX2	652, 5076
ureter development	GO:0072189	0	0.005	2	0.08	12	BMP4, PAX2	652, 5076
nephric duct morphogenesis	GO:0072178	0	0.005	2	0.08	12	BMP4, PAX2	652, 5076
positive regulation of protein kinase activity	GO:0045860	0	0	9	3.01	432	EDN3, CDK20, BMP4, DAG1, MARK2, TNIK, MAPK8IP3, PAK3, AGER	1908, 23552, 652, 1605, 2011, 23043, 23162, 5063, 177
regulation of neurogenesis	GO:0050767	0	0	11	4.25	610	MAP2, FOXO3, BMP4, CHODL, PACSIN1, DAG1, MARK2, TNIK, TCF12, PAK3, NFATC4	4133, 2309, 652, 140578, 29993, 1605, 2011, 23043, 6938, 5063, 4776

epithelial tube morphogenesis	GO:0060562	0	0.005	7	1.95	280	RBM15, BMP4, PAX2, DAG1, NPNT, PRICKLE1, NFATC4	64783, 652, 5076, 1605, 255743, 144165, 4776
Golgi localization	GO:0051645	0	0	2	0.09	13	RIPOR1, YWHAZ	79567, 7534
stem cell differentiation	GO:0048863	0	0	5	1.02	147	EDN3, FOXO4, PAX2, PRICKLE1, SOX5	1908, 4303, 5076, 144165, 6660
body morphogenesis	GO:0010171	0	0	3	0.32	46	TIPARP, CLASP1, DAG1	25976, 23332, 1605
response to amyloid-beta	GO:1904645	0	0	2	0.10	14	FOXO3, AGER	2309, 177
positive regulation of cellular metabolic process	GO:0031325	0	0.005	28	17.27	2479	EDN3, FOXO3, MUC1, BRCC3, FOXO4, IRF6, RBM15, MEN1, CDK20, BMP4, PAX2, LMX1B, DAG1, MARK2, TNIK, TCF12, SAMD4A, ESRRG, NR1I2, MAPK8IP3, NPNT, PRICKLE1, PAK3, SCOC, AGER, CNBP, NFATC4,	1908, 2309, 4582, 79184, 4303, 3664, 64783, 4221, 23552, 652, 5076, 4010, 1605, 2011, 23043, 6938, 23034, 2104, 8856, 23162, 255743, 144165, 5063, 60592, 177, 7555, 4776, 11143
positive regulation of macromolecule metabolic process	GO:0010604	0	0.007	28	17.28	2481	EDN3, FOXO3, TIPARP, MUC1, BRCC3, FOXO4, IRF6, RBM15, MEN1, CDK20, BMP4, PAX2, LMX1B, DAG1, MARK2, TNIK, TCF12, SAMD4A, ESRRG, NR1I2, MAPK8IP3, NPNT, PRICKLE1, PAK3, AGER, CNBP, NFATC4, KAT7	1908, 2309, 25976, 4582, 79184, 4303, 3664, 64783, 4221, 23552, 652, 5076, 4010, 1605, 2011, 23043, 6938, 23034, 2104, 8856, 23162, 255743, 144165, 5063, 177, 7555, 4776, 11143

positive regulation of biological process	GO:0048518	0	0.006	43	30.69	4407	ANK1, EDN3, DNMT3A, FOXO3, TIPARP, MUC1, BRCC3, FOXO4, IRF6, CLASP1, RIPOR1, RBM15, MEN1, CDK20, BMP4, ABCG1, PAX2, PRC1, LMX1B, YWHAZ, ICAM2, PACSIN1, SELENON, GRP, DAG1, MARK2, TNIK, TCF12, SAMD4A, ESRRG, NR1I2, MAPK8IP3, NPNT, PRICKLE1, PAK3, SCOC, AGER, SOX5, DDHD1, CNBP, CMKLR1, NFATC4, KAT7	286, 1908, 1788, 2309, 25976, 4582, 79184, 4303, 3664, 23332, 79567, 64783, 4221, 23552, 652, 9619, 5076, 9055, 4010, 7534, 3384, 29993, 57190, 2922, 1605, 2011, 23043, 6938, 23034, 2104, 8856, 23162, 255743, 144165, 5063, 60592, 177, 6660, 80821, 7555, 1240, 4776, 11143
metanephric renal vesicle morphogenesis	GO:0072283	0	0	2	0.10	15	BMP4, PAX2	652, 5076
regulation of glomerulus development	GO:0090192	0	0.005	2	0.10	15	BMP4, PAX2	652, 5076
nephric duct development	GO:0072176	0	0.007	2	0.10	15	BMP4, PAX2	652, 5076
positive regulation of response to oxidative stress	GO:1902884	0	0.007	2	0.10	15	FOXO3, SELENON	2309, 57190

negative regulation of gene expression	GO:0010629	0	0.005	18	9.39	1348	DNMT3A, FOXO3, TIPARP, MUC1, RBM15, MEN1, BMP4, AFF2, PAX2, SRSF7, CBX5, NOSTRIN, SHMT1, SAMD4A, NR1I2, PRICKLE1, CNBP, NFATC4	1788, 2309, 25976, 4582, 64783, 4221, 652, 2334, 5076, 6432, 23468, 115677, 6470, 23034, 8856, 144165, 7555, 4776
positive regulation of kinase activity	GO:0033674	0	0.005	9	3.22	463	EDN3, CDK20, BMP4, DAG1, MARK2, TNIK, MAPK8IP3, PAK3, AGER	1908, 23552, 652, 1605, 2011, 23043, 23162, 5063, 177
positive regulation of MAP kinase activity	GO:0043406	0	0	6	1.57	226	EDN3, BMP4, TNIK, MAPK8IP3, PAK3, AGER	1908, 652, 23043, 23162, 5063, 177
establishment of cell polarity	GO:0030010	0	0	4	0.69	99	MAP2, CLASP1, MARK3, MARK2	4133, 23332, 4140, 2011
cellular response to glucose stimulus	GO:0071333	0	0	4	0.69	99	FOXO3, MEN1, PAX2, AGER	2309, 4221, 5076, 177
positive regulation of locomotion	GO:0040017	0.005	0.009	8	2.66	382	EDN3, FOXO4, CLASP1, RIPOR1, BMP4, PAK3, AGER, CMKLR1	1908, 4303, 23332, 79567, 652, 5063, 177, 1240
muscle fiber development	GO:0048747	0.005	0.010	3	0.36	51	FLNC, BMP4, SELENON	2318, 652, 57190
cellular response to hexose stimulus	GO:0071331	0.005	0	4	0.70	101	FOXO3, MEN1, PAX2, AGER	2309, 4221, 5076, 177
response to immobilization stress	GO:0035902	0.005	0.008	2	0.11	16	FOXO3, FOXO4	2309, 4303

positive regulation of extracellular matrix organization	GO:1903055	0.005	0.005	2	0.11	16	CLASP1, DAG1	23332, 1605
negative regulation of kidney development	GO:0090185	0.005	0.006	2	0.11	16	BMP4, PAX2	652, 5076
cellular response to monosaccharide stimulus	GO:0071326	0.006	0	4	0.71	102	FOXO3, MEN1, PAX2, AGER	2309, 4221, 5076, 177
positive regulation of developmental process	GO:0051094	0.006	0.008	15	7.31	1050	EDN3, FOXO3, CLASP1, BMP4, PAX2, PACSIN1, DAG1, MARK2, TCF12, NPNT, PAK3, AGER, SOX5, DDHD1, CMKLR1	1908, 2309, 23332, 652, 5076, 29993, 1605, 2011, 6938, 255743, 5063, 177, 6660, 80821, 1240
cellular response to organic cyclic compound	GO:0071407	0.006	0	9	3.30	474	FOXO3, TIPARP, BMP4, DAG1, SHMT1, ESRRG, NR1I2, PAK3, NFATC4	2309, 25976, 652, 1605, 6470, 2104, 8856, 5063, 4776
regulation of protein kinase activity	GO:0045859	0.006	0	11	4.58	658	EDN3, NPFFR2, MEN1, CDK20, BMP4, DAG1, MARK2, TNIK, MAPK8IP3, PAK3, AGER	1908, 10886, 4221, 23552, 652, 1605, 2011, 23043, 23162, 5063, 177
regulation of cell morphogenesis	GO:0022604	0.006	0.008	8	2.73	392	MAP2, TTBK1, DAG1, MARK2, TNIK, SEMA4A, PAK3, NFATC4	4133, 84630, 1605, 2011, 23043, 64218, 5063, 4776
negative regulation of morphogenesis of an epithelium	GO:1905331	0.006	0	2	0.12	17	CLASP1, BMP4	23332, 652

mesodermal cell fate commitment	GO:0001710	0.006	0	2	0.12	17	BMP4, PAX2	652, 5076
negative regulation of muscle cell differentiation	GO:0051148	0.006	0.009	3	0.38	54	FOXO4, BMP4, PRICKLE1	4303, 652, 144165
tube morphogenesis	GO:0035239	0.006	0.009	7	2.19	315	RBM15, BMP4, PAX2, DAG1, NPNT, PRICKLE1, NFATC4	64783, 652, 5076, 1605, 255743, 144165, 4776
regulation of MAPK cascade	GO:0043408	0.006	0	10	4.00	574	EDN3, NPFFR2, MEN1, BMP4, DAG1, TNIK, MAPK8IP3, NPNT, PAK3, AGER	1908, 10886, 4221, 652, 1605, 23043, 23162, 255743, 5063, 177
cellular response to carbohydrate stimulus	GO:0071322	0.007	0	4	0.75	108	FOXO3, MEN1, PAX2, AGER	2309, 4221, 5076, 177
positive regulation of cellular process	GO:0048522	0.007	0.007	39	27.61	3965	ANK1, EDN3, DNMT3A, FOXO3, MUC1, BRCC3, FOXO4, IRF6, CLASP1, RIPOR1, RBM15, MEN1, CDK20, BMP4, PAX2, PRC1, LMX1B, YWHAZ, PACSIN1, GRP, DAG1, MARK2, TNIK, TCF12, SAMD4A, ESRRG, NR1I2, MAPK8IP3, NPNT, PRICKLE1, PAK3, SCOC, AGER, SOX5, DDHD1, CNBP, CMKLR1, NFATC4, KAT7	286, 1908, 1788, 2309, 4582, 79184, 4303, 3664, 23332, 79567, 64783, 4221, 23552, 652, 5076, 9055, 4010, 7534, 29993, 2922, 1605, 2011, 23043, 6938, 23034, 2104, 8856, 23162, 255743, 144165, 5063, 60592, 177, 6660, 80821, 7555, 1240, 4776, 11143
smooth muscle tissue development	GO:0048745	0.007	0.012	2	0.13	18	TIPARP, BMP4	25976, 652

kidney mesenchyme development	GO:0072074	0.007	0.008	2	0.13	18	BMP4, PAX2	652, 5076
negative regulation of myoblast differentiation	GO:0045662	0.007	0.007	2	0.13	18	BMP4, PRICKLE1	652, 144165
GMP metabolic process	GO:0046037	0.007	0	2	0.13	18	DLG2, IMPDH1	1740, 3614
renal vesicle morphogenesis	GO:0072077	0.007	0	2	0.13	18	BMP4, PAX2	652, 5076
branching involved in ureteric bud morphogenesis	GO:0001658	0.007	0.008	3	0.39	56	BMP4, PAX2, NPNT	652, 5076, 255743
microtubule bundle formation	GO:0001578	0.007	0.008	3	0.39	56	MAP2, CLASP1, PRC1	4133, 23332, 9055
regulation of multicellular organismal development	GO:2000026	0.007	0.007	19	10.50	1508	MAP2, FOXO3, FOXO4, CLASP1, RBM15, BMP4, PAX2, CHODL, PACSIN1, DAG1, MARK2, TNIK, TCF12, SEMA4A, PRICKLE1, PAK3, AGER, SOX5, NFATC4	4133, 2309, 4303, 23332, 64783, 652, 5076, 140578, 29993, 1605, 2011, 23043, 6938, 64218, 144165, 5063, 177, 6660, 4776
response to carbohydrate	GO:0009743	0.007	0	5	1.20	173	FOXO3, MEN1, PAX2, AGER, KAT7	2309, 4221, 5076, 177, 11143
stress-activated protein kinase signaling cascade	GO:0031098	0.007	0	6	1.71	246	MEN1, TNIK, MAPK8IP3, PAK3, AGER, KAT7	4221, 23043, 23162, 5063, 177, 11143

mesenchymal cell differentiation	GO:0048762	0.007	0.009	5	1.22	175	EDN3, CLASP1, BMP4, PAX2, DAG1	1908, 23332, 652, 5076, 1605
positive regulation of macromolecule biosynthetic process	GO:0010557	0.007	0.006	18	9.81	1408	FOXO3, MUC1, FOXO4, IRF6, RBM15, MEN1, BMP4, PAX2, LMX1B, TCF12, SAMD4A, ESRRG, NR1I2, NPNT, PAK3, CNBP, NFATC4,	2309, 4582, 4303, 3664, 64783, 4221, 652, 5076, 4010, 6938, 23034, 2104, 8856, 255743, 5063, 7555, 4776, 11143
basement membrane organization	GO:0071711	0.008	0.006	2	0.13	19	CLASP1, DAG1	23332, 1605
microtubule anchoring	GO:0034453	0.008	0.006	2	0.13	19	CLASP1, DAG1	23332, 1605
mesenchymal to epithelial transition	GO:0060231	0.008	0.006	2	0.13	19	BMP4, PAX2	652, 5076
renal vesicle development	GO:0072087	0.008	0	2	0.13	19	BMP4, PAX2	652, 5076
dendrite morphogenesis	GO:0048813	0.008	0.007	4	0.78	112	MAP2, TNIK, PAK3, NFATC4	4133, 23043, 5063, 4776
muscle cell differentiation	GO:0042692	0.008	0.009	7	2.31	331	FLNC, FOXO4, BMP4, SELENON, NPNT, PRICKLE1, NFATC4	2318, 4303, 652, 57190, 255743, 144165, 4776
positive regulation of biosynthetic process	GO:0009891	0.008	0.008	19	10.69	1535	FOXO3, MUC1, FOXO4, IRF6, RBM15, MEN1, BMP4, ABCG1, PAX2, LMX1B, TCF12, SAMD4A, ESRRG, NR1I2, NPNT, PAK3, CNBP, NFATC4, KAT7	2309, 4582, 4303, 3664, 64783, 4221, 652, 9619, 5076, 4010, 6938, 23034, 2104, 8856, 255743, 5063, 7555, 4776, 11143
positive regulation of JUN kinase activity	GO:0043507	0.008	0.005	3	0.42	60	TNIK, MAPK8IP3, AGER	23043, 23162, 177

type B pancreatic cell differentiation	GO:0003309	0.008	0.009	2	0.14	20	MEN1, BMP4	4221, 652
cellular glucose homeostasis	GO:0001678	0.008	0.007	4	0.80	115	FOXO3, MEN1, PAX2, AGER	2309, 4221, 5076, 177
regulation of protein serine/threonine kinase activity	GO:0071900	0.009	0.011	8	2.91	418	EDN3, NPFFR2, MEN1, BMP4, TNIK, MAPK8IP3, PAK3, AGER	1908, 10886, 4221, 652, 23043, 23162, 5063, 177
regulation of dendrite development	GO:0050773	0.009	0.011	4	0.81	116	PACSIN1, TNIK, PAK3, NFATC4	29993, 23043, 5063, 4776
positive regulation of nucleic acid-templated transcription	GO:1903508	0.009	0.009	16	8.43	1210	FOXO3, MUC1, FOXO4, IRF6, RBM15, MEN1, BMP4, PAX2, LMX1B, TCF12, ESRRG, NR1I2, NPNT, CNBP, NFATC4, KAT7	2309, 4582, 4303, 3664, 64783, 4221, 652, 5076, 4010, 6938, 2104, 8856, 255743, 7555, 4776, 11143
positive regulation of transcription, DNA-templated	GO:0045893	0.009	0.009	16	8.43	1210	FOXO3, MUC1, FOXO4, IRF6, RBM15, MEN1, BMP4, PAX2, LMX1B, TCF12, ESRRG, NR1I2, NPNT, CNBP, NFATC4, KAT7	2309, 4582, 4303, 3664, 64783, 4221, 652, 5076, 4010, 6938, 2104, 8856, 255743, 7555, 4776, 11143
positive regulation of RNA biosynthetic process	GO:1902680	0.009	0.010	16	8.43	1211	FOXO3, MUC1, FOXO4, IRF6, RBM15, MEN1, BMP4, PAX2, LMX1B, TCF12, ESRRG, NR1I2, NPNT, CNBP, NFATC4, KAT7	2309, 4582, 4303, 3664, 64783, 4221, 652, 5076, 4010, 6938, 2104, 8856, 255743, 7555, 4776, 11143
ureteric bud morphogenesis	GO:0060675	0.009	0.010	3	0.42	61	BMP4, PAX2, NPNT	652, 5076, 255743

neuron projection development	GO:0031175	0.009	0.013	12	5.54	796	MAP2, PAX2, NCDN, CHODL, PACSIN1, DAG1, MARK2, TNIK, SEMA4A, PAK3, AGER, NFATC4	4133, 5076, 23154, 140578, 29993, 1605, 2011, 23043, 64218, 5063, 177, 4776
dendrite development	GO:0016358	0.009	0.008	5	1.28	184	MAP2, PACSIN1, TNIK, PAK3, NFATC4	4133, 29993, 23043, 5063, 4776
mesonephric tubule morphogenes is	GO:0072171	0.009	0.010	3	0.43	62	BMP4, PAX2, NPNT	652, 5076, 255743
neuron development	GO:0048666	0.010	0.011	13	6.30	905	MAP2, PAX2, NCDN, CHODL, PACSIN1, DAG1, MARK2, TNIK, SEMA4A, PAK3, AGER, DGKG, NFATC4	4133, 5076, 23154, 140578, 29993, 1605, 2011, 23043, 64218, 5063, 177, 1608, 4776
regulation of kinase activity	GO:0043549	0.010	0.006	11	4.92	707	EDN3, NPFFR2, MEN1, CDK20, BMP4, DAG1, MARK2, TNIK, MAPK8IP3, PAK3, AGER	1908, 10886, 4221, 23552, 652, 1605, 2011, 23043, 23162, 5063, 177
regulation of cell development	GO:0060284	0.010	0.010	11	4.94	709	MAP2, FOXO3, BMP4, CHODL, PACSIN1, DAG1, MARK2, TNIK, TCF12, PAK3, NFATC4	4133, 2309, 652, 140578, 29993, 1605, 2011, 23043, 6938, 5063, 4776
positive regulation of cellular response to transforming growth factor beta stimulus	GO:1903846	0.010	0.006	2	0.15	22	MEN1, NPNT	4221, 255743
regulation of metanephros development	GO:0072215	0.010	0.009	2	0.15	22	BMP4, PAX2	652, 5076

positive regulation of transforming growth factor beta receptor signaling pathway	GO:0030511	0.010	0.006	2	0.15	22	MEN1, NPNT	4221, 255743
negative regulation of developmental process	GO:0051093	0.010	0.009	11	4.95	711	FOXO3, FOXO4, CLASP1, RBM15, MEN1, BMP4, ABCG1, PAX2, SEMA4A, PRICKLE1, NFATC4	2309, 4303, 23332, 64783, 4221, 652, 9619, 5076, 64218, 144165, 4776
positive regulation of cell migration	GO:0030335	0.010	0.011	7	2.40	345	EDN3, FOXO4, CLASP1, RIPOR1, BMP4, PAK3, CMKLR1	1908, 4303, 23332, 79567, 652, 5063, 1240
muscle structure development	GO:0061061	0.010	0.009	9	3.64	522	FLNC, FOXO4, BMP4, CHODL, SELENON, TCF12, NPNT, PRICKLE1, NFATC4	2318, 4303, 652, 140578, 57190, 6938, 255743, 144165, 4776

regulation of cellular process	GO:0050794	0.010	0.010	65	54.44	7816	ANK1, MAP2, EDN3, DNMT3A, FOXO3, TIPARP, MUC1, BRCC3, FOXO4, NPFFR2, IRF6, CLASP1, RIPOR1, RBM15, NDRG3, MEN1, TTBK1, CDK20, BMP4, DLG2, AFF2, ABCG1, CTNND1, KRBOX4, PAX2, PRC1, SRSF7, NCDN, CHODL, PEG10, LMX1B, CBX5, YWHAZ, ICAM2, NOSTRIN, PACSIN1, SELENON, MARK3, GRP, CACNA1C, DAG1, MARK2, TNIK, ZNF83, SHMT1, DTNA, TCF12, IMPA1, SAMD4A, ESRRG, NR1I2, MAPK8IP3, NPNT, SEMA4A, PRICKLE1, PAK3, SCOC, AGER, SOX5, DDHD1, DGKG, CNBP, CMKLR1, NFATC4, KAT7	286, 4133, 1908, 1788, 2309, 25976, 4582, 79184, 4303, 10886, 3664, 23332, 79567, 64783, 57446, 4221, 84630, 23552, 652, 1740, 2334, 9619, 1500, 55634, 5076, 9055, 6432, 23154, 140578, 23089, 4010, 23468, 7534, 3384, 115677, 29993, 57190, 4140, 2922, 775, 1605, 2011, 23043, 55769, 6470, 1837, 6938, 3612, 23034, 2104, 8856, 23162, 255743, 64218, 144165, 5063, 60592, 177, 6660, 80821, 1608, 7555, 1240, 4776, 11143

Supplementary Table 11. Signal transuction networks enriched by the target genes of Group 8 miRNAs

Network	Network id	P-value	Adjusted p-value	# Genes (observed)	# Genes (expected)	# Genes (total)	List of observed genes	Gene ids
ESTROGEN RECEPTOR	PW_ER_HOMO_SAPIENS	0.001	0.004	6	1.14	160	BRCA1, DACH1, RBM39, UBE2D3, ZFHX3, ESRRG	672, 1602, 9584, 7323, 463, 2104
CYCLIN B2	PW_CCNB2_HOMO_SAPIENS	0.003	0.003	2	0.08	11	PLK4, TP53	10733, 7157
RIBOSOMAL PROTEIN S6 KINASE	PW_S6K_HOMO_SAPIENS	0.004	0.002	5	1.04	146	FBXO32, FST, MAPKAP1, FMR1, PRKAA1	114907, 10468, 79109, 2332, 5562
CELL DIVISION CYCLE 25C	PW_CDC25_HOMO_SAPIENS	0.004	0.001	3	0.33	46	BRCA1, PLK4, TP53	672, 10733, 7157
PEROXISOME PROLIFERATOR ACTIVATED RECEPTOR DELTA	PW_PPARD_HOMO_SAPIENS	0.007	0.006	3	0.39	54	IL15, PRKAA1, ESRRG	3600, 5562, 2104
SWI/SNF RELATED, MATRIX ASSOCIATED, ACTIN DEPENDENT REGULATOR OF CHROMATIN, SUBFAMILY A, MEMBER 4 (BRG1)	PW_SMARCA4_HOMO_SAPIENS	0.008	0.005	3	0.42	59	ZEB1, BRCA1, MITF	6935, 672, 4286

DYSTROPHIA MYOTONICA PROTEIN KINASE	PW_DMPK_HOMO_SAPIENS	0.010	0.010	2	0.15	21	FBXO32, MORF4L1	114907, 10933
SECOND MESSENGER CAMP	PW_CAMP_HOMO_SAPIENS	0.010	0.012	5	1.33	186	CA1, MITF, GRIK2, PDE4D, TAC1	759, 4286, 2898, 5144, 6863

Supplementary Table 12. Molecular functions enriched by the target genes of Group 8 miRNAs

GO-Term	GO-Term id	P-value	Adjusted p-value	# Genes (observed)	# Genes (expected)	# Genes (total)	List of observed genes	Gene ids
poly-purine tract binding	GO:0070717	0.000	0.000	3	0.11	18	FMR1, SYNCRIP, RBPMS	2332, 10492, 11030
single-stranded RNA binding	GO:0003727	0.001	0.001	4	0.38	64	FMR1, SYNCRIP, LSM14A, RBPMS	2332, 10492, 26065, 11030
transmitter-gated ion channel activity	GO:0022824	0.001	0.002	3	0.22	38	GABRA1, HTR3D, GRIK2	2554, 200909, 2898
transmitter-gated channel activity	GO:0022835	0.001	0.002	3	0.22	38	GABRA1, HTR3D, GRIK2	2554, 200909, 2898
RNA stem-loop binding	GO:0035613	0.001	0.001	2	0.06	10	FMR1, RBPMS	2332, 11030
poly(A) binding	GO:0008143	0.002	0.002	2	0.07	12	SYNCRIP, RBPMS	10492, 11030
damaged DNA binding	GO:0003684	0.002	0.003	3	0.27	45	CUL4B, BRCA1, TP53	8450, 672, 7157
protein tyrosine kinase binding	GO:1990782	0.004	0.007	3	0.33	56	TRIM6, TP53, NRG1	117854, 7157, 3084
extracellular ligand-gated ion channel	GO:0005230	0.004	0.005	3	0.33	56	GABRA1, HTR3D, GRIK2	2554, 200909, 2898
mRNA 3'-UTR binding	GO:0003730	0.005	0.003	3	0.34	58	FMR1, CPEB2, TP53	2332, 132864, 7157
dynein complex binding	GO:0070840	0.005	0.003	2	0.11	18	BCL2L11, FMR1	10018, 2332
double-stranded DNA binding	GO:0003690	0.005	0.002	10	3.87	656	TOX2, STAT6, LSM14A, ESRRG, SUV39H2, MITF, ZFHX3, ORC4, TP53, TGIF1	84969, 6778, 26065, 2104, 79723, 4286, 463, 5000, 7157, 7050
neurotransmitter receptor activity	GO:0030594	0.005	0.007	3	0.35	59	GABRA1, HTR3D, GRIK2	2554, 200909, 2898
translation repressor activity	GO:0030371	0.007	0.008	2	0.13	22	FMR1, CPEB2	2332, 132864

transcription regulatory region DNA binding	GO:0044212	0.008	0.010	10	4.17	707	TOX2, STAT6, ESRRG, SUV39H2, MITF, ZFHX3, ZEB1, BRCA1, TP53, TGIF1	84969, 6778, 2104, 79723, 4286, 463, 6935, 672, 7157, 7050
regulatory region DNA binding	GO:0000975	0.008	0.010	10	4.18	709	TOX2, STAT6, ESRRG, SUV39H2, MITF, ZFHX3, ZEB1, BRCA1, TP53, TGIF1	84969, 6778, 2104, 79723, 4286, 463, 6935, 672, 7157, 7050
sequence-specific double-stranded DNA binding	GO:1990837	0.008	0.008	9	3.54	600	TOX2, STAT6, ESRRG, SUV39H2, MITF, ZFHX3, ORC4, TP53, TGIF1	84969, 6778, 2104, 79723, 4286, 463, 5000, 7157, 7050
regulatory region nucleic acid binding	GO:0001067	0.008	0.010	10	4.19	710	TOX2, STAT6, ESRRG, SUV39H2, MITF, ZFHX3, ZEB1, BRCA1, TP53, TGIF1	84969, 6778, 2104, 79723, 4286, 463, 6935, 672, 7157, 7050
RNA polymerase II transcription factor activity, sequence-specific DNA binding	GO:0000981	0.010	0.007	9	3.63	616	TOX2, STAT6, ESRRG, MITF, ZFHX3, ZEB1, DACH1, TP53, TGIF1	84969, 6778, 2104, 4286, 463, 6935, 1602, 7157, 7050

Supplementary Table 13. Biological processes enriched by the target genes of Group 8 miRNAs

GO-Term	GO-Term id	P-value	Adjusted p-value	# Genes (observed)	# Genes (expected)	# Genes (total)	List of observed genes	Gene ids
negative regulation of cellular macromolecule biosynthetic process	GO:2000113	0	0	22	6.61	1166	TRIM6, SUV39H2, CPEB2, ZFHX3, MORF4L2, PKIG, MITF, WWTR1, UBE2D3, FMR1, SYNCRIP, FST, STAT6, STRN3, TGIF1, MORF4L1, ZNF345, BRCA1, ZEB1, NRG1, DACH1, TP53	117854, 79723, 132864, 463, 9643, 11142, 4286, 25937, 7323, 2332, 10492, 10468, 6778, 29966, 7050, 10933, 25850, 672, 6935, 3084, 1602, 7157
negative regulation of cellular biosynthetic process	GO:0031327	0	0	23	7.20	1270	PRKAA1, TRIM6, SUV39H2, CPEB2, ZFHX3, MORF4L2, PKIG, MITF, WWTR1, UBE2D3, FMR1, SYNCRIP, FST, STAT6, STRN3, TGIF1, MORF4L1, ZNF345, BRCA1, ZEB1, NRG1, DACH1, TP53	5562, 117854, 79723, 132864, 463, 9643, 11142, 4286, 25937, 7323, 2332, 10492, 10468, 6778, 29966, 7050, 10933, 25850, 672, 6935, 3084, 1602, 7157
negative regulation of biosynthetic process	GO:0009890	0	0	23	7.28	1285	PRKAA1, TRIM6, SUV39H2, CPEB2, ZFHX3, MORF4L2, PKIG, MITF, WWTR1, UBE2D3, FMR1, SYNCRIP, FST, STAT6, STRN3, TGIF1, MORF4L1, ZNF345, BRCA1, ZEB1, NRG1, DACH1, TP53	5562, 117854, 79723, 132864, 463, 9643, 11142, 4286, 25937, 7323, 2332, 10492, 10468, 6778, 29966, 7050, 10933, 25850, 672, 6935, 3084, 1602, 7157

negative regulation of macromolecule biosynthetic process	GO:0010558	0	0	22	6.87	1212	TRIM6, SUV39H2, CPEB2, ZFHX3, MORF4L2, PKIG, MITF, WWTR1, UBE2D3, FMR1, SYNCRIP, FST, STAT6, STRN3, TGIF1, MORF4L1, ZNF345, BRCA1, ZEB1, NRG1, DACH1, TP53	117854, 79723, 132864, 463, 9643, 11142, 4286, 25937, 7323, 2332, 10492, 10468, 6778, 29966, 7050, 10933, 25850, 672, 6935, 3084, 1602, 7157
negative regulation of RNA metabolic process	GO:0051253	0	0	20	5.83	1028	TRIM6, SUV39H2, ZFHX3, MORF4L2, PKIG, MITF, WWTR1, UBE2D3, FMR1, FST, STAT6, STRN3, TGIF1, MORF4L1, ZNF345, BRCA1, ZEB1, NRG1, DACH1, TP53	117854, 79723, 463, 9643, 11142, 4286, 25937, 7323, 2332, 10468, 6778, 29966, 7050, 10933, 25850, 672, 6935, 3084, 1602, 7157
negative regulation of gene expression	GO:0010629	0	0	23	7.64	1348	TRIM6, SUV39H2, CPEB2, ZFHX3, MORF4L2, PKIG, MITF, WWTR1, UBE2D3, FMR1, CPNE1, SYNCRIP, FST, STAT6, STRN3, TGIF1, MORF4L1, ZNF345, BRCA1, ZEB1, NRG1, DACH1, TP53	117854, 79723, 132864, 463, 9643, 11142, 4286, 25937, 7323, 2332, 8904, 10492, 10468, 6778, 29966, 7050, 10933, 25850, 672, 6935, 3084, 1602, 7157
negative regulation of transcription, DNA-templated	GO:0045892	0	0	19	5.46	964	TRIM6, SUV39H2, ZFHX3, MORF4L2, PKIG, MITF, WWTR1, UBE2D3, FST, STAT6, STRN3, TGIF1, MORF4L1, ZNF345, BRCA1, ZEB1, NRG1, DACH1, TP53	117854, 79723, 463, 9643, 11142, 4286, 25937, 7323, 10468, 6778, 29966, 7050, 10933, 25850, 672, 6935, 3084, 1602, 7157
negative regulation of nucleic acid-templated transcription	GO:1903507	0	0	19	5.62	992	TRIM6, SUV39H2, ZFHX3, MORF4L2, PKIG, MITF, WWTR1, UBE2D3, FST, STAT6, STRN3, TGIF1, MORF4L1, ZNF345, BRCA1, ZEB1, NRG1, DACH1, TP53	117854, 79723, 463, 9643, 11142, 4286, 25937, 7323, 10468, 6778, 29966, 7050, 10933, 25850, 672, 6935, 3084, 1602, 7157

negative regulation of RNA biosynthetic process	GO:1902679	0	0	19	5.63	993	TRIM6, SUV39H2, ZFHX3, MORF4L2, PKIG, MITF, WWTR1, UBE2D3, FST, STAT6, STRN3, TGIF1, MORF4L1, ZNF345, BRCA1, ZEB1, NRG1, DACH1, TP53	117854, 79723, 463, 9643, 11142, 4286, 25937, 7323, 10468, 6778, 29966, 7050, 10933, 25850, 672, 6935, 3084, 1602, 7157
negative regulation of nucleobase-containing compound metabolic process	GO:0045934	0	0	20	6.46	1139	TRIM6, SUV39H2, ZFHX3, MORF4L2, PKIG, MITF, WWTR1, UBE2D3, FMR1, FST, STAT6, STRN3, TGIF1, MORF4L1, ZNF345, BRCA1, ZEB1, NRG1, DACH1, TP53	117854, 79723, 463, 9643, 11142, 4286, 25937, 7323, 2332, 10468, 6778, 29966, 7050, 10933, 25850, 672, 6935, 3084, 1602, 7157
cellular response to chemical stimulus	GO:0070887	0	0	29	12.67	2235	PRKAA1, LSM14A, TRIM6, GJB6, ESRRG, SUV39H2, CPEB2, FBXO32, TAC1, BCL2L11, UBE2D3, FMR1, CYB5R3, CPNE1, ATXN3, GABRA1, SYNCRIP, STAT6, STRN3, TGIF1, CTSB, PDE4D, NCOA7, AHCYL1, BRCA1, ZEB1, TP53, ADGRE2, IL15	5562, 26065, 117854, 10804, 2104, 79723, 132864, 114907, 6863, 10018, 7323, 2332, 1727, 8904, 4287, 2554, 10492, 6778, 29966, 7050, 1508, 5144, 135112, 10768, 672, 6935, 7157, 30817, 3600
negative regulation of nitrogen compound metabolic process	GO:0051172	0	0	26	10.61	1872	DBNDD2, PRKAA1, TRIM6, SUV39H2, CPEB2, ZFHX3, MORF4L2, PKIG, MITF, WWTR1, UBE2D3, FMR1, SYNCRIP, FST, STAT6, STRN3, TGIF1, PDE4D, NCOA7, MORF4L1, ZNF345, BRCA1, ZEB1, NRG1, DACH1, TP53	55861, 5562, 117854, 79723, 132864, 463, 9643, 11142, 4286, 25937, 7323, 2332, 10492, 10468, 6778, 29966, 7050, 5144, 135112, 10933, 25850, 672, 6935, 3084, 1602, 7157

negative regulation of macromolecule metabolic process	GO:0010605	0	0	26	11.30	1993	DBNDD2, TRIM6, SUV39H2, CPEB2, ZFHX3, MORF4L2, PKIG, MITF, WWTR1, UBE2D3, FMR1, CPNE1, SYNCRIP, FST, STAT6, STRN3, TGIF1, PDE4D, NCOA7, MORF4L1, ZNF345, BRCA1, ZEB1, NRG1, DACH1, TP53	55861, 117854, 79723, 132864, 463, 9643, 11142, 4286, 25937, 7323, 2332, 8904, 10492, 10468, 6778, 29966, 7050, 5144, 135112, 10933, 25850, 672, 6935, 3084, 1602, 7157
negative regulation of cellular metabolic process	GO:0031324	0	0	26	11.31	1995	DBNDD2, PRKAA1, TRIM6, SUV39H2, CPEB2, ZFHX3, MORF4L2, PKIG, MITF, WWTR1, UBE2D3, FMR1, SYNCRIP, FST, STAT6, STRN3, TGIF1, PDE4D, NCOA7, MORF4L1, ZNF345, BRCA1, ZEB1, NRG1, DACH1, TP53	55861, 5562, 117854, 79723, 132864, 463, 9643, 11142, 4286, 25937, 7323, 2332, 10492, 10468, 6778, 29966, 7050, 5144, 135112, 10933, 25850, 672, 6935, 3084, 1602, 7157
regulation of cellular macromolecule biosynthetic process	GO:2000112	0	0	35	18.37	3240	RBM39, PRKAA1, LSM14A, TRIM6, ESRRG, SUV39H2, CPEB2, ZFHX3, MORF4L2, PKIG, MITF, WWTR1, UBE2D3, FMR1, TOX2, CPNE1, ATXN3, SYNCRIP, FST, STAT6, STRN3, TGIF1, RBPMS, MCTS1, SCML1, NCOA7, MORF4L1, ZNF345, BRCA1, ZEB1, NRG1, DACH1, TP53, EYA4, IL15	9584, 5562, 26065, 117854, 2104, 79723, 132864, 463, 9643, 11142, 4286, 25937, 7323, 2332, 84969, 8904, 4287, 10492, 10468, 6778, 29966, 7050, 11030, 28985, 6322, 135112, 10933, 25850, 672, 6935, 3084, 1602, 7157, 2070, 3600

negative regulation of metabolic process	GO:0009892	0	0	27	12.39	2186	DBNDD2, PRKAA1, TRIM6, SUV39H2, CPEB2, ZFHX3, MORF4L2, PKIG, MITF, WWTR1, UBE2D3, FMR1, CPNE1, SYNCRIP, FST, STAT6, STRN3, TGIF1, PDE4D, NCOA7, MORF4L1, ZNF345, BRCA1, ZEB1, NRG1, DACH1, TP53	55861, 5562, 117854, 79723, 132864, 463, 9643, 11142, 4286, 25937, 7323, 2332, 8904, 10492, 10468, 6778, 29966, 7050, 5144, 135112, 10933, 25850, 672, 6935, 3084, 1602, 7157
response to chemical	GO:0042221	0	0	33	17.11	3018	PRKAA1, LSM14A, TRIM6, GJB6, ESRRG, SUV39H2, CPEB2, FBXO32, ZFHX3, TAC1, BCL2L11, UBE2D3, FMR1, CYB5R3, CPNE1, ATXN3, GABRA1, SYNCRIP, STAT6, STRN3, TGIF1, CTSB, PDE4D, NCOA7, SLC30A8, AHCYL1, BRCA1, ZEB1, NRG1, TP53, CACNA1G,	5562, 26065, 117854, 10804, 2104, 79723, 132864, 114907, 463, 6863, 10018, 7323, 2332, 1727, 8904, 4287, 2554, 10492, 6778, 29966, 7050, 1508, 5144, 135112, 169026, 10768, 672, 6935, 3084, 7157, 8913, 30817, 3600
regulation of macromolecule biosynthetic process	GO:0010556	0	0	35	18.83	3321	RBM39, PRKAA1, LSM14A, TRIM6, ESRRG, SUV39H2, CPEB2, ZFHX3, MORF4L2, PKIG, MITF, WWTR1, UBE2D3, FMR1, TOX2, CPNE1, ATXN3, SYNCRIP, FST, STAT6, STRN3, TGIF1, RBPMS, MCTS1, SCML1, NCOA7, MORF4L1, ZNF345, BRCA1, ZEB1, NRG1, DACH1, TP53, EYA4, IL15	9584, 5562, 26065, 117854, 2104, 79723, 132864, 463, 9643, 11142, 4286, 25937, 7323, 2332, 84969, 8904, 4287, 10492, 10468, 6778, 29966, 7050, 11030, 28985, 6322, 135112, 10933, 25850, 672, 6935, 3084, 1602, 7157, 2070, 3600
cellular response to virus	GO:0098586	0	0	4	0.19	34	LSM14A, TRIM6, BCL2L11, FMR1	26065, 117854, 10018, 2332

cellular response to organic substance	GO:0071310	0	0	24	10.70	1888	PRKAA1, LSM14A, TRIM6, GJB6, ESRRG, CPEB2, FBXO32, TAC1, BCL2L11, UBE2D3, CPNE1, ATXN3, GABRA1, SYNCRIP, STAT6, STRN3, TGIF1, CTSB, PDE4D, AHCYL1, BRCA1, ZEB1, TP53, IL15	5562, 26065, 117854, 10804, 2104, 132864, 114907, 6863, 10018, 7323, 8904, 4287, 2554, 10492, 6778, 29966, 7050, 1508, 5144, 10768, 672, 6935, 7157, 3600
regulation of RNA metabolic process	GO:0051252	0	0	32	17.06	3010	RBM39, PRKAA1, TRIM6, ESRRG, SUV39H2, ZFHX3, MORF4L2, PKIG, MITF, WWTR1, UBE2D3, FMR1, TOX2, CPNE1, ATXN3, FST, STAT6, STRN3, TGIF1, RBPMS, MCTS1, SCML1, NCOA7, MORF4L1, ZNF345, AHCYL1, BRCA1, ZEB1, NRG1, DACH1, TP53, EYA4	9584, 5562, 117854, 2104, 79723, 463, 9643, 11142, 4286, 25937, 7323, 2332, 84969, 8904, 4287, 10468, 6778, 29966, 7050, 11030, 28985, 6322, 135112, 10933, 25850, 10768, 672, 6935, 3084, 1602, 7157, 2070
regulation of cellular biosynthetic process	GO:0031326	0	0	35	19.67	3470	RBM39, PRKAA1, LSM14A, TRIM6, ESRRG, SUV39H2, CPEB2, ZFHX3, MORF4L2, PKIG, MITF, WWTR1, UBE2D3, FMR1, TOX2, CPNE1, ATXN3, SYNCRIP, FST, STAT6, STRN3, TGIF1, RBPMS, MCTS1, SCML1, NCOA7, MORF4L1, ZNF345, BRCA1, ZEB1, NRG1, DACH1, TP53, EYA4, IL15	9584, 5562, 26065, 117854, 2104, 79723, 132864, 463, 9643, 11142, 4286, 25937, 7323, 2332, 84969, 8904, 4287, 10492, 10468, 6778, 29966, 7050, 11030, 28985, 6322, 135112, 10933, 25850, 672, 6935, 3084, 1602, 7157, 2070, 3600

negative regulation of transcription from RNA polymerase II promoter	GO:0000122	0	0	13	3.79	669	SUV39H2, ZFHX3, PKIG, MITF, WWTR1, UBE2D3, FST, STAT6, TGIF1, ZNF345, ZEB1, DACH1, TP53	79723, 463, 11142, 4286, 25937, 7323, 10468, 6778, 7050, 25850, 6935, 1602, 7157
cellular macromolecule biosynthetic process	GO:0034645	0	0	38	22.38	3948	RBM39, PRKAA1, LSM14A, TRIM6, ESRRG, SUV39H2, ORC4, CPEB2, ZFHX3, MORF4L2, PKIG, MITF, WWTR1, UBE2D3, FMR1, TOX2, RPAIN, CPNE1, ATXN3, SYNCRIP, FST, STAT6, STRN3, TGIF1, RBPMS, MCTS1, SCML1, NCOA7, MORF4L1, ZNF345, BRCA1, ZEB1, ATG4A, NRG1, DACH1, TP53, EYA4, IL15	9584, 5562, 26065, 117854, 2104, 79723, 5000, 132864, 463, 9643, 11142, 4286, 25937, 7323, 2332, 84969, 84268, 8904, 4287, 10492, 10468, 6778, 29966, 7050, 11030, 28985, 6322, 135112, 10933, 25850, 672, 6935, 115201, 3084, 1602, 7157, 2070, 3600
response to organic substance	GO:0010033	0	0	27	13.38	2360	PRKAA1, LSM14A, TRIM6, GJB6, ESRRG, CPEB2, FBXO32, ZFHX3, TAC1, BCL2L11, UBE2D3, FMR1, CPNE1, ATXN3, GABRA1, SYNCRIP, STAT6, STRN3, TGIF1, CTSB, PDE4D, SLC30A8, AHCYL1, BRCA1, ZEB1, TP53, IL15	5562, 26065, 117854, 10804, 2104, 132864, 114907, 463, 6863, 10018, 7323, 2332, 8904, 4287, 2554, 10492, 6778, 29966, 7050, 1508, 5144, 169026, 10768, 672, 6935, 7157, 3600

regulation of biosynthetic process	GO:0009889	0	0	35	19.99	3526	RBM39, PRKAA1, LSM14A, TRIM6, ESRRG, SUV39H2, CPEB2, ZFHX3, MORF4L2, PKIG, MITF, WWTR1, UBE2D3, FMR1, TOX2, CPNE1, ATXN3, SYNCRIP, FST, STAT6, STRN3, TGIF1, RBPMS, MCTS1, SCML1, NCOA7, MORF4L1, ZNF345, BRCA1, ZEB1, NRG1, DACH1, TP53, EYA4, IL15	9584, 5562, 26065, 117854, 2104, 79723, 132864, 463, 9643, 11142, 4286, 25937, 7323, 2332, 84969, 8904, 4287, 10492, 10468, 6778, 29966, 7050, 11030, 28985, 6322, 135112, 10933, 25850, 672, 6935, 3084, 1602, 7157, 2070, 3600
regulation of gene expression	GO:0010468	0	0	35	20.03	3534	RBM39, PRKAA1, LSM14A, TRIM6, ESRRG, SUV39H2, CPEB2, ZFHX3, MORF4L2, PKIG, MITF, WWTR1, UBE2D3, FMR1, TOX2, CPNE1, ATXN3, SYNCRIP, FST, STAT6, STRN3, TGIF1, RBPMS, MCTS1, SCML1, NCOA7, MORF4L1, ZNF345, AHCYL1, BRCA1, ZEB1, NRG1, DACH1, TP53, EYA4	9584, 5562, 26065, 117854, 2104, 79723, 132864, 463, 9643, 11142, 4286, 25937, 7323, 2332, 84969, 8904, 4287, 10492, 10468, 6778, 29966, 7050, 11030, 28985, 6322, 135112, 10933, 25850, 10768, 672, 6935, 3084, 1602, 7157, 2070

macromolecule biosynthetic process	GO:0009059	0	0	38	23.02	4061	RBM39, PRKAA1, LSM14A, TRIM6, ESRRG, SUV39H2, ORC4, CPEB2, ZFHX3, MORF4L2, PKIG, MITF, WWTR1, UBE2D3, FMR1, TOX2, RPAIN, CPNE1, ATXN3, SYNCRIP, FST, STAT6, STRN3, TGIF1, RBPMS, MCTS1, SCML1, NCOA7, MORF4L1, ZNF345, BRCA1, ZEB1, ATG4A, NRG1, DACH1, TP53, EYA4, IL15	9584, 5562, 26065, 117854, 2104, 79723, 5000, 132864, 463, 9643, 11142, 4286, 25937, 7323, 2332, 84969, 84268, 8904, 4287, 10492, 10468, 6778, 29966, 7050, 11030, 28985, 6322, 135112, 10933, 25850, 672, 6935, 115201, 3084, 1602, 7157, 2070, 3600
regulation of nitrogen compound metabolic process	GO:0051171	0	0	41	25.84	4558	DBNDD2, RBM39, PRKAA1, LSM14A, TRIM6, CUL4B, ESRRG, SUV39H2, CPEB2, ZFHX3, MORF4L2, PKIG, MITF, BCL2L11, WWTR1, UBE2D3, FMR1, TOX2, CPNE1, ATXN3, SYNCRIP, FST, STAT6, STRN3, TGIF1, RBPMS, MCTS1, GRIK2, PDE4D, SCML1, NCOA7, MORF4L1, ZNF345, AHCYL1, BRCA1, ZEB1, NRG1, DACH1, TP53, EYA4, IL15	55861, 9584, 5562, 26065, 117854, 8450, 2104, 79723, 132864, 463, 9643, 11142, 4286, 10018, 25937, 7323, 2332, 84969, 8904, 4287, 10492, 10468, 6778, 29966, 7050, 11030, 28985, 2898, 5144, 6322, 135112, 10933, 25850, 10768, 672, 6935, 3084, 1602, 7157, 2070, 3600

regulation of nucleobase-containing compound metabolic process	GO:0019219	0	0.002	33	18.67	3293	RBM39, PRKAA1, TRIM6, ESRRG, SUV39H2, ZFHX3, MORF4L2, PKIG, MITF, WWTR1, UBE2D3, FMR1, TOX2, CPNE1, ATXN3, FST, STAT6, STRN3, TGIF1, RBPMS, MCTS1, PDE4D, SCML1, NCOA7, MORF4L1, ZNF345, AHCYL1, BRCA1, ZEB1, NRG1, DACH1, TP53, EYA4	9584, 5562, 117854, 2104, 79723, 463, 9643, 11142, 4286, 25937, 7323, 2332, 84969, 8904, 4287, 10468, 6778, 29966, 7050, 11030, 28985, 5144, 6322, 135112, 10933, 25850, 10768, 672, 6935, 3084, 1602, 7157, 2070
regulation of transcription, DNA-templated	GO:0006355	0	0.001	30	16.30	2876	RBM39, PRKAA1, TRIM6, ESRRG, SUV39H2, ZFHX3, MORF4L2, PKIG, MITF, WWTR1, UBE2D3, TOX2, CPNE1, ATXN3, FST, STAT6, STRN3, TGIF1, RBPMS, MCTS1, SCML1, NCOA7, MORF4L1, ZNF345, BRCA1, ZEB1, NRG1, DACH1, TP53, EYA4	9584, 5562, 117854, 2104, 79723, 463, 9643, 11142, 4286, 25937, 7323, 84969, 8904, 4287, 10468, 6778, 29966, 7050, 11030, 28985, 6322, 135112, 10933, 25850, 672, 6935, 3084, 1602, 7157, 2070
regulation of nucleic acid-templated transcription	GO:1903506	0	0.002	30	16.41	2894	RBM39, PRKAA1, TRIM6, ESRRG, SUV39H2, ZFHX3, MORF4L2, PKIG, MITF, WWTR1, UBE2D3, TOX2, CPNE1, ATXN3, FST, STAT6, STRN3, TGIF1, RBPMS, MCTS1, SCML1, NCOA7, MORF4L1, ZNF345, BRCA1, ZEB1, NRG1, DACH1, TP53, EYA4	9584, 5562, 117854, 2104, 79723, 463, 9643, 11142, 4286, 25937, 7323, 84969, 8904, 4287, 10468, 6778, 29966, 7050, 11030, 28985, 6322, 135112, 10933, 25850, 672, 6935, 3084, 1602, 7157, 2070

cellular response to DNA damage stimulus	GO:0006974	0	0	12	3.67	648	CUL4B, MORF4L2, BCL2L11, UBE2D3, FMR1, RPAIN, ATXN3, MCTS1, MORF4L1, BRCA1, TP53, EYA4	8450, 9643, 10018, 7323, 2332, 84268, 4287, 28985, 10933, 672, 7157, 2070
regulation of RNA biosynthetic process	GO:2001141	0	0.002	30	16.45	2902	RBM39, PRKAA1, TRIM6, ESRRG, SUV39H2, ZFHX3, MORF4L2, PKIG, MITF, WWTR1, UBE2D3, TOX2, CPNE1, ATXN3, FST, STAT6, STRN3, TGIF1, RBPMS, MCTS1, SCML1, NCOA7, MORF4L1, ZNF345, BRCA1, ZEB1, NRG1, DACH1, TP53, EYA4	9584, 5562, 117854, 2104, 79723, 463, 9643, 11142, 4286, 25937, 7323, 84969, 8904, 4287, 10468, 6778, 29966, 7050, 11030, 28985, 6322, 135112, 10933, 25850, 672, 6935, 3084, 1602, 7157, 2070
cellular nitrogen compound biosynthetic process	GO:0044271	0	0	36	21.72	3832	RBM39, PRKAA1, LSM14A, TRIM6, ESRRG, SUV39H2, CPEB2, ZFHX3, MORF4L2, PKIG, MITF, WWTR1, UBE2D3, FMR1, TOX2, CPNE1, ATXN3, SYNCRIP, FST, STAT6, ADK, STRN3, TGIF1, RBPMS, MCTS1, SCML1, NCOA7, MORF4L1, ATP5G3, ZNF345, BRCA1, ZEB1, NRG1, DACH1, TP53, EYA4	9584, 5562, 26065, 117854, 2104, 79723, 132864, 463, 9643, 11142, 4286, 25937, 7323, 2332, 84969, 8904, 4287, 10492, 10468, 6778, 132, 29966, 7050, 11030, 28985, 6322, 135112, 10933, 518, 25850, 672, 6935, 3084, 1602, 7157, 2070

regulation of primary metabolic process	GO:0080090	0	0	41	26.58	4689	DBNDD2, RBM39, PRKAA1, LSM14A, TRIM6, CUL4B, ESRRG, SUV39H2, CPEB2, ZFHX3, MORF4L2, PKIG, MITF, BCL2L11, WWTR1, UBE2D3, FMR1, TOX2, CPNE1, ATXN3, SYNCRIP, FST, STAT6, STRN3, TGIF1, RBPMS, MCTS1, GRIK2, PDE4D, SCML1, NCOA7, MORF4L1, ZNF345, AHCYL1, BRCA1, ZEB1, NRG1, DACH1, TP53, EYA4, IL15	55861, 9584, 5562, 26065, 117854, 8450, 2104, 79723, 132864, 463, 9643, 11142, 4286, 10018, 25937, 7323, 2332, 84969, 8904, 4287, 10492, 10468, 6778, 29966, 7050, 11030, 28985, 2898, 5144, 6322, 135112, 10933, 25850, 10768, 672, 6935, 3084, 1602, 7157, 2070, 3600
histone modification	GO:0016570	0	0	9	2.24	396	PRKAA1, CUL4B, SUV39H2, MORF4L2, FMR1, MORF4L1, BRCA1, TP53, EYA4	5562, 8450, 79723, 9643, 2332, 10933, 672, 7157, 2070
cellular response to stimulus	GO:0051716	0	0	44	29.53	5209	PRKAA1, LSM14A, TRIM6, GJB6, CUL4B, ESRRG, SUV39H2, CPEB2, FBXO32, MORF4L2, TAC1, PKIG, BCL2L11, WWTR1, UBE2D3, FMR1, RPAIN, CYB5R3, CPNE1, ATXN3, GABRA1, HTR3D, SYNCRIP, FST, STAT6, STRN3, TGIF1, RBPMS, MCTS1, GRIK2, CD302, CTSB, PDE4D, NCOA7, MORF4L1, AHCYL1, BRCA1, ZEB1, NRG1, TP53, ADGRE2, EYA4, IL15, MAPKAP1	5562, 26065, 117854, 10804, 8450, 2104, 79723, 132864, 114907, 9643, 6863, 11142, 10018, 25937, 7323, 2332, 84268, 1727, 8904, 4287, 2554, 200909, 10492, 10468, 6778, 29966, 7050, 11030, 28985, 2898, 9936, 1508, 5144, 135112, 10933, 10768, 672, 6935, 3084, 7157, 30817, 2070, 3600, 79109

regulation of macromolecule metabolic process	GO:0060255	0	0.001	41	26.64	4700	DBNDD2, RBM39, PRKAA1, LSM14A, TRIM6, CUL4B, ESRRG, SUV39H2, CPEB2, ZFHX3, MORF4L2, PKIG, MITF, BCL2L11, WWTR1, UBE2D3, FMR1, TOX2, CPNE1, ATXN3, SYNCRIP, FST, STAT6, STRN3, TGIF1, RBPMS, MCTS1, GRIK2, PDE4D, SCML1, NCOA7, MORF4L1, ZNF345, AHCYL1, BRCA1, ZEB1, NRG1, DACH1, TP53, EYA4, IL15	55861, 9584, 5562, 26065, 117854, 8450, 2104, 79723, 132864, 463, 9643, 11142, 4286, 10018, 25937, 7323, 2332, 84969, 8904, 4287, 10492, 10468, 6778, 29966, 7050, 11030, 28985, 2898, 5144, 6322, 135112, 10933, 25850, 10768, 672, 6935, 3084, 1602, 7157, 2070, 3600
regulation of intracellular transport	GO:0032386	0	0.001	10	2.76	487	PKIG, BCL2L11, WWTR1, UBE2D3, FMR1, PREPL, RBPMS, NRG1, TP53, ADGRE2	11142, 10018, 25937, 7323, 2332, 9581, 11030, 3084, 7157, 30817
DNA repair	GO:0006281	0	0	9	2.28	403	CUL4B, MORF4L2, UBE2D3, RPAIN, ATXN3, MORF4L1, BRCA1, TP53, EYA4	8450, 9643, 7323, 84268, 4287, 10933, 672, 7157, 2070

organic substance biosynthetic process	GO:1901576	0	0.001	42	27.73	4891	RBM39, PRKAA1, LSM14A, TRIM6, ESRRG, G6PC2, SUV39H2, ORC4, CPEB2, ZFHX3, MORF4L2, PKIG, MITF, WWTR1, UBE2D3, FMR1, TOX2, RPAIN, CYB5R3, CPNE1, ATXN3, SYNCRIP, FST, STAT6, ADK, STRN3, TGIF1, RBPMS, MCTS1, SCML1, NCOA7, MORF4L1, ATP5G3, ZNF345, BRCA1, ZEB1, ATG4A, NRG1, DACH1, TP53,	9584, 5562, 26065, 117854, 2104, 57818, 79723, 5000, 132864, 463, 9643, 11142, 4286, 25937, 7323, 2332, 84969, 84268, 1727, 8904, 4287, 10492, 10468, 6778, 132, 29966, 7050, 11030, 28985, 6322, 135112, 10933, 518, 25850, 672, 6935, 115201, 3084, 1602, 7157, 2070, 3600
response to stress	GO:0006950	0	0	29	16.14	2847	PRKAA1, LSM14A, TRIM6, CUL4B, SUV39H2, CPEB2, MORF4L2, TAC1, BCL2L11, UBE2D3, FMR1, RPAIN, ATXN3, SYNCRIP, STAT6, RBPMS, MCTS1, GRIK2, CTSB, NCOA7, MORF4L1, SLC30A8, BRCA1, NRG1, TP53, ADGRE2, EYA4, IL15, MAPKAP1	5562, 26065, 117854, 8450, 79723, 132864, 9643, 6863, 10018, 7323, 2332, 84268, 4287, 10492, 6778, 11030, 28985, 2898, 1508, 135112, 10933, 169026, 672, 3084, 7157, 30817, 2070, 3600, 79109
transcription, DNA-templated	GO:0006351	0	0.002	30	17.03	3004	RBM39, PRKAA1, TRIM6, ESRRG, SUV39H2, ZFHX3, MORF4L2, PKIG, MITF, WWTR1, UBE2D3, TOX2, CPNE1, ATXN3, FST, STAT6, STRN3, TGIF1, RBPMS, MCTS1, SCML1, NCOA7, MORF4L1, ZNF345, BRCA1, ZEB1, NRG1, DACH1, TP53, EYA4	9584, 5562, 117854, 2104, 79723, 463, 9643, 11142, 4286, 25937, 7323, 84969, 8904, 4287, 10468, 6778, 29966, 7050, 11030, 28985, 6322, 135112, 10933, 25850, 672, 6935, 3084, 1602, 7157, 2070

positive regulation of cytokine-mediated signaling pathway	GO:0001961	0.001	0	3	0.16	28	LSM14A, TRIM6, CPNE1	26065, 117854, 8904
nucleic acid-templated transcription	GO:0097659	0.001	0.002	30	17.10	3017	RBM39, PRKAA1, TRIM6, ESRRG, SUV39H2, ZFHX3, MORF4L2, PKIG, MITF, WWTR1, UBE2D3, TOX2, CPNE1, ATXN3, FST, STAT6, STRN3, TGIF1, RBPMS, MCTS1, SCML1, NCOA7, MORF4L1, ZNF345, BRCA1, ZEB1, NRG1, DACH1, TP53, EYA4	9584, 5562, 117854, 2104, 79723, 463, 9643, 11142, 4286, 25937, 7323, 84969, 8904, 4287, 10468, 6778, 29966, 7050, 11030, 28985, 6322, 135112, 10933, 25850, 672, 6935, 3084, 1602, 7157, 2070
biosynthetic process	GO:0009058	0.001	0.001	42	27.99	4937	RBM39, PRKAA1, LSM14A, TRIM6, ESRRG, G6PC2, SUV39H2, ORC4, CPEB2, ZFHX3, MORF4L2, PKIG, MITF, WWTR1, UBE2D3, FMR1, TOX2, RPAIN, CYB5R3, CPNE1, ATXN3, SYNCRIP, FST, STAT6, ADK, STRN3, TGIF1, RBPMS, MCTS1, SCML1, NCOA7, MORF4L1, ATP5G3, ZNF345, BRCA1, ZEB1, ATG4A, NRG1, DACH1, TP53,	9584, 5562, 26065, 117854, 2104, 57818, 79723, 5000, 132864, 463, 9643, 11142, 4286, 25937, 7323, 2332, 84969, 84268, 1727, 8904, 4287, 10492, 10468, 6778, 132, 29966, 7050, 11030, 28985, 6322, 135112, 10933, 518, 25850, 672, 6935, 115201, 3084, 1602, 7157, 2070, 3600

nucleobase-containing compound metabolic process	GO:0006139	0.001	0	39	25.16	4439	RBM39, PRKAA1, TRIM6, CUL4B, ESRRG, SUV39H2, ORC4, ZFHX3, MORF4L2, PKIG, MITF, WWTR1, UBE2D3, FMR1, TOX2, RPAIN, CPNE1, ATXN3, SYNCRIP, FST, STAT6, ADK, STRN3, TGIF1, RBPMS, MCTS1, PDE4D, SCML1, NCOA7, MORF4L1, ATP5G3, ZNF345, AHCYL1, BRCA1, ZEB1, NRG1, DACH1, TP53, EYA4	9584, 5562, 117854, 8450, 2104, 79723, 5000, 463, 9643, 11142, 4286, 25937, 7323, 2332, 84969, 84268, 8904, 4287, 10492, 10468, 6778, 132, 29966, 7050, 11030, 28985, 5144, 6322, 135112, 10933, 518, 25850, 10768, 672, 6935, 3084, 1602, 7157, 2070
RNA biosynthetic process	GO:0032774	0.001	0.002	30	17.17	3029	RBM39, PRKAA1, TRIM6, ESRRG, SUV39H2, ZFHX3, MORF4L2, PKIG, MITF, WWTR1, UBE2D3, TOX2, CPNE1, ATXN3, FST, STAT6, STRN3, TGIF1, RBPMS, MCTS1, SCML1, NCOA7, MORF4L1, ZNF345, BRCA1, ZEB1, NRG1, DACH1, TP53, EYA4	9584, 5562, 117854, 2104, 79723, 463, 9643, 11142, 4286, 25937, 7323, 84969, 8904, 4287, 10468, 6778, 29966, 7050, 11030, 28985, 6322, 135112, 10933, 25850, 672, 6935, 3084, 1602, 7157, 2070

nucleic acid metabolic process	GO:0090304	0.001	0	36	22.47	3964	RBM39, PRKAA1, TRIM6, CUL4B, ESRRG, SUV39H2, ORC4, ZFHX3, MORF4L2, PKIG, MITF, WWTR1, UBE2D3, FMR1, TOX2, RPAIN, CPNE1, ATXN3, SYNCRIP, FST, STAT6, STRN3, TGIF1, RBPMS, MCTS1, SCML1, NCOA7, MORF4L1, ZNF345, AHCYL1, BRCA1, ZEB1, NRG1, DACH1, TP53, EYA4	9584, 5562, 117854, 8450, 2104, 79723, 5000, 463, 9643, 11142, 4286, 25937, 7323, 2332, 84969, 84268, 8904, 4287, 10492, 10468, 6778, 29966, 7050, 11030, 28985, 6322, 135112, 10933, 25850, 10768, 672, 6935, 3084, 1602, 7157, 2070
positive regulation of type I interferon-mediated signaling pathway	GO:0060340	0.001	0	2	0.04	7	LSM14A, TRIM6	26065, 117854
positive regulation of response to cytokine stimulus	GO:0060760	0.001	0	3	0.18	31	LSM14A, TRIM6, CPNE1	26065, 117854, 8904
regulation of transport	GO:0051049	0.001	0	19	8.74	1541	PRKAA1, TRIM6, G6PC2, TAC1, PKIG, BCL2L11, WWTR1, UBE2D3, FMR1, PREPL, RBPMS, PDE4D, SLC30A8, AHCYL1, NRG1, TP53, CACNA1G, ADGRE2, GOSR1	5562, 117854, 57818, 6863, 11142, 10018, 25937, 7323, 2332, 9581, 11030, 5144, 169026, 10768, 3084, 7157, 8913, 30817, 9527

organic cyclic compound biosynthetic process	GO:1901362	0.001	0.002	33	19.98	3525	RBM39, PRKAA1, TRIM6, ESRRG, SUV39H2, ZFHX3, MORF4L2, PKIG, MITF, WWTR1, UBE2D3, TOX2, CYB5R3, CPNE1, ATXN3, FST, STAT6, ADK, STRN3, TGIF1, RBPMS, MCTS1, SCML1, NCOA7, MORF4L1, ATP5G3, ZNF345, BRCA1, ZEB1, NRG1, DACH1, TP53, EYA4	9584, 5562, 117854, 2104, 79723, 463, 9643, 11142, 4286, 25937, 7323, 84969, 1727, 8904, 4287, 10468, 6778, 132, 29966, 7050, 11030, 28985, 6322, 135112, 10933, 518, 25850, 672, 6935, 3084, 1602, 7157, 2070
nucleobase-containing compound biosynthetic process	GO:0034654	0.001	0.002	32	19.12	3373	RBM39, PRKAA1, TRIM6, ESRRG, SUV39H2, ZFHX3, MORF4L2, PKIG, MITF, WWTR1, UBE2D3, TOX2, CPNE1, ATXN3, FST, STAT6, ADK, STRN3, TGIF1, RBPMS, MCTS1, SCML1, NCOA7, MORF4L1, ATP5G3, ZNF345, BRCA1, ZEB1, NRG1, DACH1, TP53, EYA4	9584, 5562, 117854, 2104, 79723, 463, 9643, 11142, 4286, 25937, 7323, 84969, 8904, 4287, 10468, 6778, 132, 29966, 7050, 11030, 28985, 6322, 135112, 10933, 518, 25850, 672, 6935, 3084, 1602, 7157, 2070
cellular response to nitrogen compound	GO:1901699	0.001	0.002	10	2.99	528	PRKAA1, CPEB2, FMR1, GABRA1, STAT6, PDE4D, AHCYL1, BRCA1, ZEB1, TP53	5562, 132864, 2332, 2554, 6778, 5144, 10768, 672, 6935, 7157
cellular response to stress	GO:0033554	0.001	0	19	8.78	1549	PRKAA1, CUL4B, SUV39H2, CPEB2, MORF4L2, BCL2L11, UBE2D3, FMR1, RPAIN, ATXN3, STAT6, MCTS1, GRIK2, NCOA7, MORF4L1, BRCA1, TP53, EYA4, MAPKAP1	5562, 8450, 79723, 132864, 9643, 10018, 7323, 2332, 84268, 4287, 6778, 28985, 2898, 135112, 10933, 672, 7157, 2070, 79109

negative regulation of cytoplasmic	GO:2000766	0.001	0	2	0.05	8	CPEB2, FMR1	132864, 2332
organic cyclic compound metabolic process	GO:1901360	0.001	0	40	26.70	4709	RBM39, PRKAA1, TRIM6, CUL4B, ESRRG, SUV39H2, ORC4, ZFHX3, MORF4L2, PKIG, MITF, WWTR1, UBE2D3, FMR1, TOX2, RPAIN, CYB5R3, CPNE1, ATXN3, SYNCRIP, FST, STAT6, ADK, STRN3, TGIF1, RBPMS, MCTS1, PDE4D, SCML1, NCOA7, MORF4L1, ATP5G3, ZNF345, AHCYL1, BRCA1, ZEB1, NRG1, DACH1, TP53, EYA4	9584, 5562, 117854, 8450, 2104, 79723, 5000, 463, 9643, 11142, 4286, 25937, 7323, 2332, 84969, 84268, 1727, 8904, 4287, 10492, 10468, 6778, 132, 29966, 7050, 11030, 28985, 5144, 6322, 135112, 10933, 518, 25850, 10768, 672, 6935, 3084, 1602, 7157, 2070
heterocycle biosynthetic process	GO:0018130	0.001	0.002	32	19.39	3421	RBM39, PRKAA1, TRIM6, ESRRG, SUV39H2, ZFHX3, MORF4L2, PKIG, MITF, WWTR1, UBE2D3, TOX2, CPNE1, ATXN3, FST, STAT6, ADK, STRN3, TGIF1, RBPMS, MCTS1, SCML1, NCOA7, MORF4L1, ATP5G3, ZNF345, BRCA1, ZEB1, NRG1, DACH1, TP53, EYA4	9584, 5562, 117854, 2104, 79723, 463, 9643, 11142, 4286, 25937, 7323, 84969, 8904, 4287, 10468, 6778, 132, 29966, 7050, 11030, 28985, 6322, 135112, 10933, 518, 25850, 672, 6935, 3084, 1602, 7157, 2070

aromatic compound biosynthetic process	GO:0019438	0.001	0.002	32	19.40	3423	RBM39, PRKAA1, TRIM6, ESRRG, SUV39H2, ZFHX3, MORF4L2, PKIG, MITF, WWTR1, UBE2D3, TOX2, CPNE1, ATXN3, FST, STAT6, ADK, STRN3, TGIF1, RBPMS, MCTS1, SCML1, NCOA7, MORF4L1, ATP5G3, ZNF345, BRCA1, ZEB1, NRG1, DACH1, TP53, EYA4	9584, 5562, 117854, 2104, 79723, 463, 9643, 11142, 4286, 25937, 7323, 84969, 8904, 4287, 10468, 6778, 132, 29966, 7050, 11030, 28985, 6322, 135112, 10933, 518, 25850, 672, 6935, 3084, 1602, 7157, 2070
heterocycle metabolic process	GO:0046483	0.001	0	39	25.77	4546	RBM39, PRKAA1, TRIM6, CUL4B, ESRRG, SUV39H2, ORC4, ZFHX3, MORF4L2, PKIG, MITF, WWTR1, UBE2D3, FMR1, TOX2, RPAIN, CPNE1, ATXN3, SYNCRIP, FST, STAT6, ADK, STRN3, TGIF1, RBPMS, MCTS1, PDE4D, SCML1, NCOA7, MORF4L1, ATP5G3, ZNF345, AHCYL1, BRCA1, ZEB1, NRG1, DACH1, TP53, EYA4	9584, 5562, 117854, 8450, 2104, 79723, 5000, 463, 9643, 11142, 4286, 25937, 7323, 2332, 84969, 84268, 8904, 4287, 10492, 10468, 6778, 132, 29966, 7050, 11030, 28985, 5144, 6322, 135112, 10933, 518, 25850, 10768, 672, 6935, 3084, 1602, 7157, 2070
regulation of cellular localization	GO:0060341	0.001	0.003	12	4.27	754	PKIG, BCL2L11, WWTR1, UBE2D3, FMR1, PREPL, RBPMS, PDE4D, NRG1, TP53, ADGRE2, GOSR1	11142, 10018, 25937, 7323, 2332, 9581, 11030, 5144, 3084, 7157, 30817, 9527

RNA metabolic process	GO:0016070	0.001	0.003	33	20.33	3586	RBM39, PRKAA1, TRIM6, ESRRG, SUV39H2, ZFHX3, MORF4L2, PKIG, MITF, WWTR1, UBE2D3, FMR1, TOX2, CPNE1, ATXN3, SYNCRIP, FST, STAT6, STRN3, TGIF1, RBPMS, MCTS1, SCML1, NCOA7, MORF4L1, ZNF345, AHCYL1, BRCA1, ZEB1, NRG1, DACH1, TP53, EYA4	9584, 5562, 117854, 2104, 79723, 463, 9643, 11142, 4286, 25937, 7323, 2332, 84969, 8904, 4287, 10492, 10468, 6778, 29966, 7050, 11030, 28985, 6322, 135112, 10933, 25850, 10768, 672, 6935, 3084, 1602, 7157, 2070
DNA metabolic process	GO:0006259	0.001	0	12	4.31	760	CUL4B, ORC4, MORF4L2, UBE2D3, RPAIN, ATXN3, STAT6, MORF4L1, BRCA1, DACH1, TP53, EYA4	8450, 5000, 9643, 7323, 84268, 4287, 6778, 10933, 672, 1602, 7157, 2070
cellular aromatic compound metabolic process	GO:0006725	0.001	0	39	25.91	4570	RBM39, PRKAA1, TRIM6, CUL4B, ESRRG, SUV39H2, ORC4, ZFHX3, MORF4L2, PKIG, MITF, WWTR1, UBE2D3, FMR1, TOX2, RPAIN, CPNE1, ATXN3, SYNCRIP, FST, STAT6, ADK, STRN3, TGIF1, RBPMS, MCTS1, PDE4D, SCML1, NCOA7, MORF4L1, ATP5G3, ZNF345, AHCYL1, BRCA1, ZEB1, NRG1, DACH1, TP53, EYA4	9584, 5562, 117854, 8450, 2104, 79723, 5000, 463, 9643, 11142, 4286, 25937, 7323, 2332, 84969, 84268, 8904, 4287, 10492, 10468, 6778, 132, 29966, 7050, 11030, 28985, 5144, 6322, 135112, 10933, 518, 25850, 10768, 672, 6935, 3084, 1602, 7157, 2070

gene expression	GO:0010467	0.001	0.002	36	23.32	4113	RBM39, PRKAA1, LSM14A, TRIM6, ESRRG, SUV39H2, CPEB2, ZFHX3, MORF4L2, PKIG, MITF, WWTR1, UBE2D3, FMR1, TOX2, CPNE1, ATXN3, SYNCRIP, RHBDL2, FST, STAT6, STRN3, TGIF1, RBPMS, MCTS1, SCML1, NCOA7, MORF4L1, ZNF345, AHCYL1, BRCA1, ZEB1, NRG1, DACH1, TP53, EYA4	9584, 5562, 26065, 117854, 2104, 79723, 132864, 463, 9643, 11142, 4286, 25937, 7323, 2332, 84969, 8904, 4287, 10492, 54933, 10468, 6778, 29966, 7050, 11030, 28985, 6322, 135112, 10933, 25850, 10768, 672, 6935, 3084, 1602, 7157, 2070
cellular nitrogen compound metabolic process	GO:0034641	0.001	0	41	28.03	4945	RBM39, PRKAA1, LSM14A, TRIM6, CUL4B, ESRRG, SUV39H2, ORC4, CPEB2, ZFHX3, MORF4L2, PKIG, MITF, WWTR1, UBE2D3, FMR1, TOX2, RPAIN, CPNE1, ATXN3, SYNCRIP, FST, STAT6, ADK, STRN3, TGIF1, RBPMS, MCTS1, PDE4D, SCML1, NCOA7, MORF4L1, ATP5G3, ZNF345, AHCYL1, BRCA1, ZEB1, NRG1, DACH1, TP53, EYA4	9584, 5562, 26065, 117854, 8450, 2104, 79723, 5000, 132864, 463, 9643, 11142, 4286, 25937, 7323, 2332, 84969, 84268, 8904, 4287, 10492, 10468, 6778, 132, 29966, 7050, 11030, 28985, 5144, 6322, 135112, 10933, 518, 25850, 10768, 672, 6935, 3084, 1602, 7157, 2070

regulation of cellular metabolic process	GO:0031323	0.001	0.002	40	27.21	4799	DBNDD2, RBM39, PRKAA1, LSM14A, TRIM6, ESRRG, SUV39H2, CPEB2, ZFHX3, MORF4L2, PKIG, MITF, BCL2L11, WWTR1, UBE2D3, FMR1, TOX2, CPNE1, ATXN3, SYNCRIP, FST, STAT6, STRN3, TGIF1, RBPMS, MCTS1, GRIK2, PDE4D, SCML1, NCOA7, MORF4L1, ZNF345, AHCYL1, BRCA1, ZEB1, NRG1, DACH1, TP53, EYA4, IL15	55861, 9584, 5562, 26065, 117854, 2104, 79723, 132864, 463, 9643, 11142, 4286, 10018, 25937, 7323, 2332, 84969, 8904, 4287, 10492, 10468, 6778, 29966, 7050, 11030, 28985, 2898, 5144, 6322, 135112, 10933, 25850, 10768, 672, 6935, 3084, 1602, 7157, 2070, 3600
regulation of transcription from RNA polymerase II promoter	GO:0006357	0.001	0.001	19	9.29	1639	ESRRG, SUV39H2, ZFHX3, MORF4L2, PKIG, MITF, WWTR1, UBE2D3, TOX2, FST, STAT6, TGIF1, NCOA7, ZNF345, BRCA1, ZEB1, NRG1, DACH1, TP53	2104, 79723, 463, 9643, 11142, 4286, 25937, 7323, 84969, 10468, 6778, 7050, 135112, 25850, 672, 6935, 3084, 1602, 7157
cellular biosynthetic process	GO:0044249	0.002	0.003	40	27.26	4809	RBM39, PRKAA1, LSM14A, TRIM6, ESRRG, SUV39H2, ORC4, CPEB2, ZFHX3, MORF4L2, PKIG, MITF, WWTR1, UBE2D3, FMR1, TOX2, RPAIN, CPNE1, ATXN3, SYNCRIP, FST, STAT6, ADK, STRN3, TGIF1, RBPMS, MCTS1, SCML1, NCOA7, MORF4L1, ATP5G3, ZNF345, BRCA1, ZEB1, ATG4A, NRG1, DACH1, TP53,	9584, 5562, 26065, 117854, 2104, 79723, 5000, 132864, 463, 9643, 11142, 4286, 25937, 7323, 2332, 84969, 84268, 8904, 4287, 10492, 10468, 6778, 132, 29966, 7050, 11030, 28985, 6322, 135112, 10933, 518, 25850, 672, 6935, 115201, 3084, 1602, 7157, 2070, 3600

covalent chromatin modification	GO:0016569	0.002	0.001	9	2.77	488	PRKAA1, CUL4B, SUV39H2, MORF4L2, FMR1, MORF4L1, BRCA1, TP53, EYA4	5562, 8450, 79723, 9643, 2332, 10933, 672, 7157, 2070
regulation of circadian rhythm	GO:0042752	0.002	0.001	4	0.52	91	PRKAA1, SUV39H2, ZFHX3, TP53	5562, 79723, 463, 7157
regulation of cellular process	GO:0050794	0.002	0	56	44.31	7816	DBNDD2, RBM39, PRKAA1, LSM14A, TRIM6, GJB6, CUL4B, ESRRG, G6PC2, SUV39H2, CPEB2, ZFHX3, MORF4L2, TAC1, PKIG, MITF, BCL2L11, WWTR1, UBE2D3, FMR1, TOX2, CPNE1, PREPL, ATXN3, GABRA1, HTR3D, SYNCRIP, SEPT9, FST, STAT6, STRN3, TGIF1, RBPMS, MCTS1, GRIK2, CD302, CTSB, PDE4D, SCML1, PLK4, NCOA7, MORF4L1, SLC30A8, ZNF345, AHCYL1, BRCA1, ZEB1, NRG1, DACH1, TP53, CACNA1G, ADGRE2, EYA4, GOSR1, IL15, MAPKAP1	55861, 9584, 5562, 26065, 117854, 10804, 8450, 2104, 57818, 79723, 132864, 463, 9643, 6863, 11142, 4286, 10018, 25937, 7323, 2332, 84969, 8904, 9581, 4287, 2554, 200909, 10492, 10801, 10468, 6778, 29966, 7050, 11030, 28985, 2898, 9936, 1508, 5144, 6322, 10733, 135112, 10933, 169026, 25850, 10768, 672, 6935, 3084, 1602, 7157, 8913, 30817, 2070, 9527, 3600, 79109
positive regulation of establishment of protein localization to	GO:1903749	0.002	0.002	4	0.53	94	BCL2L11, UBE2D3, NRG1, TP53	10018, 7323, 3084, 7157
cellular response to hypoxia	GO:0071456	0.002	0.004	5	0.90	158	PRKAA1, SUV39H2, CPEB2, UBE2D3, TP53	5562, 79723, 132864, 7323, 7157
negative regulation of intracellular estrogen receptor	GO:0033147	0.002	0	2	0.07	12	STRN3, BRCA1	29966, 672

cytoplasmic translation	GO:0002181	0.002	0.005	3	0.26	46	CPEB2, FMR1, MCTS1	132864, 2332, 28985
negative regulation of cellular process	GO:0048523	0.002	0.005	32	20.40	3598	DBNDD2, PRKAA1, TRIM6, GJB6, SUV39H2, CPEB2, ZFHX3, MORF4L2, PKIG, MITF, WWTR1, UBE2D3, FMR1, CPNE1, SYNCRIP, FST, STAT6, STRN3, TGIF1, GRIK2, PDE4D, NCOA7, MORF4L1, ZNF345, BRCA1, ZEB1, NRG1, DACH1, TP53, EYA4, IL15, MAPKAP1	55861, 5562, 117854, 10804, 79723, 132864, 463, 9643, 11142, 4286, 25937, 7323, 2332, 8904, 10492, 10468, 6778, 29966, 7050, 2898, 5144, 135112, 10933, 25850, 672, 6935, 3084, 1602, 7157, 2070, 3600, 79109
cellular response to decreased oxygen levels	GO:0036294	0.002	0.004	5	0.93	164	PRKAA1, SUV39H2, CPEB2, UBE2D3, TP53	5562, 79723, 132864, 7323, 7157
positive regulation of neuron apoptotic process	GO:0043525	0.002	0.002	3	0.27	47	BCL2L11, GRIK2, TP53	10018, 2898, 7157
positive regulation of biological process	GO:0048518	0.002	0.002	37	24.98	4407	PRKAA1, LSM14A, TRIM6, CUL4B, ESRRG, ZFHX3, MORF4L2, TAC1, MITF, BCL2L11, WWTR1, UBE2D3, FMR1, TOX2, CPNE1, ATXN3, SEPT9, FST, STAT6, RBPMS, MCTS1, GRIK2, CTSB, PDE4D, PLK4, NCOA7, SLC30A8, AHCYL1, BRCA1, ZEB1, NRG1, TP53, CACNA1G, ADGRE2, EYA4, IL15, MAPKAP1	5562, 26065, 117854, 8450, 2104, 463, 9643, 6863, 4286, 10018, 25937, 7323, 2332, 84969, 8904, 4287, 10801, 10468, 6778, 11030, 28985, 2898, 1508, 5144, 10733, 135112, 169026, 10768, 672, 6935, 3084, 7157, 8913, 30817, 2070, 3600, 79109

cellular response to endogenous stimulus	GO:0071495	0.002	0.003	14	6.07	1071	PRKAA1, ESRRG, CPEB2, FBXO32, TAC1, UBE2D3, GABRA1, STRN3, CTSB, PDE4D, AHCYL1, BRCA1, ZEB1, TP53	5562, 2104, 132864, 114907, 6863, 7323, 2554, 29966, 1508, 5144, 10768, 672, 6935, 7157
positive regulation of cellular component biogenesis	GO:0044089	0.003	0.001	8	2.40	423	CUL4B, TAC1, MITF, BCL2L11, FMR1, SEPT9, PLK4, TP53	8450, 6863, 4286, 10018, 2332, 10801, 10733, 7157
regulation of metabolic process	GO:0019222	0.003	0.002	41	28.96	5108	DBNDD2, RBM39, PRKAA1, LSM14A, TRIM6, CUL4B, ESRRG, SUV39H2, CPEB2, ZFHX3, MORF4L2, PKIG, MITF, BCL2L11, WWTR1, UBE2D3, FMR1, TOX2, CPNE1, ATXN3, SYNCRIP, FST, STAT6, STRN3, TGIF1, RBPMS, MCTS1, GRIK2, PDE4D, SCML1, NCOA7, MORF4L1, ZNF345, AHCYL1, BRCA1, ZEB1, NRG1, DACH1, TP53, EYA4, IL15	55861, 9584, 5562, 26065, 117854, 8450, 2104, 79723, 132864, 463, 9643, 11142, 4286, 10018, 25937, 7323, 2332, 84969, 8904, 4287, 10492, 10468, 6778, 29966, 7050, 11030, 28985, 2898, 5144, 6322, 135112, 10933, 25850, 10768, 672, 6935, 3084, 1602, 7157, 2070, 3600
regulation of centriole replication	GO:0046599	0.003	0.002	2	0.08	14	PLK4, BRCA1	10733, 672
histone H2A acetylation	GO:0043968	0.003	0.002	2	0.08	14	MORF4L2, MORF4L1	9643, 10933

cellular macromolecule metabolic process	GO:0044260	0.003	0.003	49	37.18	6558	DBNDD2, RBM39, CALU, PRKAA1, LSM14A, TRIM6, CUL4B, ESRRG, SUV39H2, ORC4, CPEB2, FBXO32, ZFHX3, MORF4L2, PKIG, MITF, BCL2L11, WWTR1, UBE2D3, FMR1, TOX2, RPAIN, CPNE1, ATXN3, SYNCRIP, FST, STAT6, STRN3, TGIF1, RBPMS, PPP2R2B, MCTS1, GRIK2, CTSB, PDE4D, SCML1, PLK4, NCOA7, MORF4L1, ZNF345, AHCYL1, BRCA1, ZEB1, ATG4A, NRG1, DACH1, TP53, EYA4, IL15	55861, 9584, 813, 5562, 26065, 117854, 8450, 2104, 79723, 5000, 132864, 114907, 463, 9643, 11142, 4286, 10018, 25937, 7323, 2332, 84969, 84268, 8904, 4287, 10492, 10468, 6778, 29966, 7050, 11030, 5521, 28985, 2898, 1508, 5144, 6322, 10733, 135112, 10933, 25850, 10768, 672, 6935, 115201, 3084, 1602, 7157, 2070, 3600
regulation of establishment of protein localization to mitochondrion	GO:1903747	0.003	0.003	4	0.60	105	BCL2L11, UBE2D3, NRG1, TP53	10018, 7323, 3084, 7157
positive regulation of organelle organization	GO:0010638	0.003	0.001	9	3.01	531	TAC1, BCL2L11, UBE2D3, FMR1, SEPT9, PLK4, BRCA1, NRG1, TP53	6863, 10018, 7323, 2332, 10801, 10733, 672, 3084, 7157
cellular response to oxygen levels	GO:0071453	0.003	0.004	5	0.98	173	PRKAA1, SUV39H2, CPEB2, UBE2D3, TP53	5562, 79723, 132864, 7323, 7157
regulation of regulated secretory pathway	GO:1903305	0.003	0.004	4	0.60	106	FMR1, PREPL, CACNA1G, ADGRE2	2332, 9581, 8913, 30817
response to nitrogen compound	GO:1901698	0.003	0.003	12	4.89	863	PRKAA1, CPEB2, TAC1, FMR1, ATXN3, GABRA1, STAT6, PDE4D, AHCYL1, BRCA1, ZEB1, TP53	5562, 132864, 6863, 2332, 4287, 2554, 6778, 5144, 10768, 672, 6935, 7157
regulation of SMAD protein import into	GO:0060390	0.003	0.002	2	0.09	15	WWTR1, RBPMS	25937, 11030

thymocyte apoptotic process	GO:0070242	0.003	0.007	2	0.09	15	BCL2L11, TP53	10018, 7157
DNA damage response, signal transduction by p53 class mediator resulting in transcription of p21 class mediator	GO:0006978	0.003	0.004	2	0.09	15	BRCA1, TP53	672, 7157
response to UV	GO:0009411	0.003	0.006	4	0.61	108	PRKAA1, CUL4B, FMR1, TP53	5562, 8450, 2332, 7157
response to endogenous stimulus	GO:0009719	0.003	0.004	15	6.99	1233	PRKAA1, ESRRG, CPEB2, FBXO32, ZFHX3, TAC1, UBE2D3, GABRA1, STRN3, CTSB, PDE4D, AHCYL1, BRCA1, ZEB1, TP53	5562, 2104, 132864, 114907, 463, 6863, 7323, 2554, 29966, 1508, 5144, 10768, 672, 6935, 7157
response to oxygen-containing compound	GO:1901700	0.003	0.006	15	7.00	1234	PRKAA1, TRIM6, GJB6, CPEB2, FBXO32, TAC1, STAT6, STRN3, PDE4D, SLC30A8, AHCYL1, BRCA1, ZEB1, TP53, IL15	5562, 117854, 10804, 132864, 114907, 6863, 6778, 29966, 5144, 169026, 10768, 672, 6935, 7157, 3600
positive regulation of response to stimulus	GO:0048584	0.004	0.001	18	9.24	1630	PRKAA1, LSM14A, TRIM6, TAC1, BCL2L11, UBE2D3, FMR1, CPNE1, ATXN3, STAT6, RBPMS, CTSB, PDE4D, BRCA1, NRG1, TP53, EYA4, IL15	5562, 26065, 117854, 6863, 10018, 7323, 2332, 8904, 4287, 6778, 11030, 1508, 5144, 672, 3084, 7157, 2070, 3600
DNA damage response, signal transduction resulting in transcription	GO:0042772	0.004	0.005	2	0.09	16	BRCA1, TP53	672, 7157

single-organism localization	GO:1902578	0.004	0.002	25	14.97	2640	PRKAA1, TRIM6, G6PC2, TAC1, PKIG, BCL2L11, WWTR1, FMR1, RPAIN, CYB5R3, CPNE1, PREPL, RBPMS, GRIK2, CD302, CTSB, PDE4D, PLK4, SLC30A8, ATP5G3, AHCYL1, NRG1, TP53, CACNA1G, ADGRE2	5562, 117854, 57818, 6863, 11142, 10018, 25937, 2332, 84268, 1727, 8904, 9581, 11030, 2898, 9936, 1508, 5144, 10733, 169026, 518, 10768, 3084, 7157, 8913, 30817
macromolecule metabolic process	GO:0043170	0.004	0.007	51	39.81	7023	DBNDD2, RBM39, CALU, PRKAA1, LSM14A, TRIM6, CUL4B, ESRRG, SUV39H2, ORC4, CPEB2, FBXO32, ZFHX3, MORF4L2, PKIG, MITF, BCL2L11, WWTR1, UBE2D3, FMR1, TOX2, RPAIN, CPNE1, PREPL, ATXN3, SYNCRIP, RHBDL2, FST, STAT6, STRN3, TGIF1, RBPMS, PPP2R2B, MCTS1, GRIK2, CTSB, PDE4D, SCML1, PLK4, NCOA7, MORF4L1, ZNF345, AHCYL1, BRCA1, ZEB1, ATG4A, NRG1, DACH1, TP53, EYA4, IL15	55861, 9584, 813, 5562, 26065, 117854, 8450, 2104, 79723, 5000, 132864, 114907, 463, 9643, 11142, 4286, 10018, 25937, 7323, 2332, 84969, 84268, 8904, 9581, 4287, 10492, 54933, 10468, 6778, 29966, 7050, 11030, 5521, 28985, 2898, 1508, 5144, 6322, 10733, 135112, 10933, 25850, 10768, 672, 6935, 115201, 3084, 1602, 7157, 2070, 3600

response to stimulus	GO:0050896	0.004	0.002	47	35.61	6282	PRKAA1, LSM14A, TRIM6, GJB6, CUL4B, ESRRG, SUV39H2, CPEB2, FBXO32, ZFHX3, MORF4L2, TAC1, PKIG, BCL2L11, WWTR1, UBE2D3, FMR1, RPAIN, CYB5R3, CPNE1, ATXN3, GABRA1, HTR3D, SYNCRIP, FST, STAT6, STRN3, TGIF1, RBPMS, MCTS1, GRIK2, CD302, CTSB, PDE4D, NCOA7, MORF4L1, SLC30A8, AHCYL1, BRCA1, ZEB1, NRG1, TP53, CACNA1G, ADGRE2, EYA4, IL15, MAPKAP1	5562, 26065, 117854, 10804, 8450, 2104, 79723, 132864, 114907, 463, 9643, 6863, 11142, 10018, 25937, 7323, 2332, 84268, 1727, 8904, 4287, 2554, 200909, 10492, 10468, 6778, 29966, 7050, 11030, 28985, 2898, 9936, 1508, 5144, 135112, 10933, 169026, 10768, 672, 6935, 3084, 7157, 8913, 30817, 2070, 3600, 79109
cellular response to organonitrogen compound	GO:0071417	0.004	0.006	8	2.60	459	PRKAA1, CPEB2, GABRA1, PDE4D, AHCYL1, BRCA1, ZEB1, TP53	5562, 132864, 2554, 5144, 10768, 672, 6935, 7157
histone H4 acetylation	GO:0043967	0.005	0.002	3	0.33	59	MORF4L2, MORF4L1, BRCA1	9643, 10933, 672
cellular response to UV	GO:0034644	0.005	0.004	3	0.33	59	CUL4B, FMR1, TP53	8450, 2332, 7157
positive regulation of transport	GO:0051050	0.005	0.004	11	4.47	788	TRIM6, TAC1, BCL2L11, UBE2D3, FMR1, RBPMS, SLC30A8, AHCYL1, NRG1, TP53, CACNA1G	117854, 6863, 10018, 7323, 2332, 11030, 169026, 10768, 3084, 7157, 8913
transcription from RNA polymerase II promoter	GO:0006366	0.005	0.004	19	10.25	1808	ESRRG, SUV39H2, ZFHX3, MORF4L2, PKIG, MITF, WWTR1, UBE2D3, TOX2, FST, STAT6, TGIF1, NCOA7, ZNF345, BRCA1, ZEB1, NRG1, DACH1, TP53	2104, 79723, 463, 9643, 11142, 4286, 25937, 7323, 84969, 10468, 6778, 7050, 135112, 25850, 672, 6935, 3084, 1602, 7157

negative regulation of biological process	GO:0048519	0.005	0.006	33	22.23	3921	DBNDD2, PRKAA1, TRIM6, GJB6, SUV39H2, CPEB2, ZFHX3, MORF4L2, TAC1, PKIG, MITF, WWTR1, UBE2D3, FMR1, CPNE1, SYNCRIP, FST, STAT6, STRN3, TGIF1, GRIK2, PDE4D, NCOA7, MORF4L1, ZNF345, BRCA1, ZEB1, NRG1, DACH1, TP53, EYA4, IL15, MAPKAP1	55861, 5562, 117854, 10804, 79723, 132864, 463, 9643, 6863, 11142, 4286, 25937, 7323, 2332, 8904, 10492, 10468, 6778, 29966, 7050, 2898, 5144, 135112, 10933, 25850, 672, 6935, 3084, 1602, 7157, 2070, 3600, 79109
regulation of response to stimulus	GO:0048583	0.005	0.003	27	16.90	2981	PRKAA1, LSM14A, TRIM6, FBXO32, TAC1, BCL2L11, WWTR1, UBE2D3, FMR1, CPNE1, ATXN3, FST, STAT6, STRN3, RBPMS, GRIK2, CTSB, PDE4D, NCOA7, BRCA1, ZEB1, NRG1, TP53, ADGRE2, EYA4, IL15, MAPKAP1	5562, 26065, 117854, 114907, 6863, 10018, 25937, 7323, 2332, 8904, 4287, 10468, 6778, 29966, 11030, 2898, 1508, 5144, 135112, 672, 6935, 3084, 7157, 30817, 2070, 3600, 79109
regulation of defense response to virus by host	GO:0050691	0.005	0.005	2	0.11	19	TRIM6, IL15	117854, 3600
positive regulation of response to DNA damage stimulus	GO:2001022	0.005	0.002	3	0.35	62	FMR1, BRCA1, EYA4	2332, 672, 2070

protein metabolic process	GO:0019538	0.005	0.006	36	25.07	4422	DBNDD2, CALU, PRKAA1, LSM14A, TRIM6, CUL4B, SUV39H2, CPEB2, FBXO32, MORF4L2, PKIG, MITF, BCL2L11, WWTR1, UBE2D3, FMR1, CPNE1, PREPL, ATXN3, SYNCRIP, RHBDL2, RBPMS, PPP2R2B, MCTS1, GRIK2, CTSB, PDE4D, PLK4, NCOA7, MORF4L1, BRCA1, ATG4A, NRG1, TP53, EYA4, IL15	55861, 813, 5562, 26065, 117854, 8450, 79723, 132864, 114907, 9643, 11142, 4286, 10018, 25937, 7323, 2332, 8904, 9581, 4287, 10492, 54933, 11030, 5521, 28985, 2898, 1508, 5144, 10733, 135112, 10933, 672, 115201, 3084, 7157, 2070, 3600
regulation of biological process	GO:0050789	0.005	0.002	57	46.89	8272	DBNDD2, RBM39, PRKAA1, LSM14A, TRIM6, GJB6, CUL4B, ESRRG, G6PC2, SUV39H2, CPEB2, FBXO32, ZFHX3, MORF4L2, TAC1, PKIG, MITF, BCL2L11, WWTR1, UBE2D3, FMR1, TOX2, CPNE1, PREPL, ATXN3, GABRA1, HTR3D, SYNCRIP, SEPT9, FST, STAT6, STRN3, TGIF1, RBPMS, MCTS1, GRIK2, CD302, CTSB, PDE4D, SCML1, PLK4, NCOA7, MORF4L1, SLC30A8, ZNF345, AHCYL1, BRCA1, ZEB1, NRG1, DACH1, TP53, CACNA1G, ADGRE2, EYA4, GOSR1, IL15, MAPKAP1	55861, 9584, 5562, 26065, 117854, 10804, 8450, 2104, 57818, 79723, 132864, 114907, 463, 9643, 6863, 11142, 4286, 10018, 25937, 7323, 2332, 84969, 8904, 9581, 4287, 2554, 200909, 10492, 10801, 10468, 6778, 29966, 7050, 11030, 28985, 2898, 9936, 1508, 5144, 6322, 10733, 135112, 10933, 169026, 25850, 10768, 672, 6935, 3084, 1602, 7157, 8913, 30817, 2070, 9527, 3600, 79109

regulation of response to stress	GO:0080134	0.006	0.008	13	5.95	1050	LSM14A, TRIM6, TAC1, BCL2L11, UBE2D3, FMR1, ATXN3, GRIK2, CTSB, NCOA7, BRCA1, EYA4, IL15	26065, 117854, 6863, 10018, 7323, 2332, 4287, 2898, 1508, 135112, 672, 2070, 3600
primary metabolic process	GO:0044238	0.006	0.007	55	44.65	7876	DBNDD2, RBM39, CALU, PRKAA1, LSM14A, TRIM6, CUL4B, ESRRG, G6PC2, SUV39H2, ORC4, CPEB2, FBXO32, ZFHX3, MORF4L2, PKIG, MITF, BCL2L11, WWTR1, UBE2D3, FMR1, TOX2, RPAIN, CYB5R3, CPNE1, PREPL, ATXN3, SYNCRIP, RHBDL2, FST, STAT6, ADK, STRN3, TGIF1, RBPMS, PPP2R2B, MCTS1, GRIK2, CTSB, PDE4D, SCML1, PLK4, NCOA7, MORF4L1, ATP5G3, ZNF345, AHCYL1, BRCA1, ZEB1, ATG4A, NRG1, DACH1, TP53,	55861, 9584, 813, 5562, 26065, 117854, 8450, 2104, 57818, 79723, 5000, 132864, 114907, 463, 9643, 11142, 4286, 10018, 25937, 7323, 2332, 84969, 84268, 1727, 8904, 9581, 4287, 10492, 54933, 10468, 6778, 132, 29966, 7050, 11030, 5521, 28985, 2898, 1508, 5144, 6322, 10733, 135112, 10933, 518, 25850, 10768, 672, 6935, 115201, 3084, 1602, 7157, 2070, 3600
regulation of establishment of protein localization	GO:0070201	0.006	0.004	10	3.96	699	TRIM6, G6PC2, PKIG, BCL2L11, WWTR1, UBE2D3, RBPMS, SLC30A8, NRG1, TP53	117854, 57818, 11142, 10018, 25937, 7323, 11030, 169026, 3084, 7157
regulation of secretion	GO:0051046	0.006	0.005	9	3.34	590	TRIM6, G6PC2, TAC1, FMR1, PREPL, SLC30A8, NRG1, CACNA1G, ADGRE2	117854, 57818, 6863, 2332, 9581, 169026, 3084, 8913, 30817
regulation of calcium ion-dependent exocytosis	GO:0017158	0.006	0.009	3	0.37	65	FMR1, PREPL, CACNA1G	2332, 9581, 8913

negative regulation of heart contraction	GO:0045822	0.006	0.008	2	0.12	21	TAC1, PDE4D	6863, 5144
nitrogen compound metabolic process	GO:0006807	0.006	0.009	53	42.65	7523	DBNDD2, RBM39, CALU, PRKAA1, LSM14A, TRIM6, CUL4B, ESRRG, SUV39H2, ORC4, CPEB2, FBXO32, ZFHX3, MORF4L2, PKIG, MITF, BCL2L11, WWTR1, UBE2D3, FMR1, TOX2, RPAIN, CPNE1, PREPL, ATXN3, SYNCRIP, RHBDL2, FST, STAT6, ADK, STRN3, TGIF1, RBPMS, PPP2R2B, MCTS1, GRIK2, CTSB, PDE4D, SCML1, PLK4, NCOA7, MORF4L1, ATP5G3, ZNF345, AHCYL1, BRCA1, ZEB1, ATG4A, NRG1, DACH1, TP53, EYA4, IL15	55861, 9584, 813, 5562, 26065, 117854, 8450, 2104, 79723, 5000, 132864, 114907, 463, 9643, 11142, 4286, 10018, 25937, 7323, 2332, 84969, 84268, 8904, 9581, 4287, 10492, 54933, 10468, 6778, 132, 29966, 7050, 11030, 5521, 28985, 2898, 1508, 5144, 6322, 10733, 135112, 10933, 518, 25850, 10768, 672, 6935, 115201, 3084, 1602, 7157, 2070, 3600
response to inorganic substance	GO:0010035	0.007	0.004	7	2.23	393	PRKAA1, FMR1, CPNE1, STAT6, SLC30A8, AHCYL1, CACNA1G	5562, 2332, 8904, 6778, 169026, 10768, 8913
cellular response to oxygen-containing compound	GO:1901701	0.007	0.006	11	4.72	832	PRKAA1, GJB6, CPEB2, FBXO32, STAT6, PDE4D, AHCYL1, BRCA1, ZEB1, TP53, IL15	5562, 10804, 132864, 114907, 6778, 5144, 10768, 672, 6935, 7157, 3600
regulation of cytoplasmic translation	GO:2000765	0.007	0.006	2	0.12	22	CPEB2, FMR1	132864, 2332
SMAD protein import into	GO:0007184	0.007	0.009	2	0.12	22	WWTR1, RBPMS	25937, 11030

positive regulation of protein oligomerization	GO:0032461	0.007	0.005	2	0.12	22	BCL2L11, TP53	10018, 7157
response to biotic stimulus	GO:0009607	0.007	0.003	9	3.43	605	LSM14A, TRIM6, GJB6, TAC1, BCL2L11, FMR1, PDE4D, TP53, IL15	26065, 117854, 10804, 6863, 10018, 2332, 5144, 7157, 3600
histone deacetylation	GO:0016575	0.007	0.007	3	0.39	69	MORF4L2, MORF4L1, TP53	9643, 10933, 7157
positive regulation of immune system process	GO:0002684	0.007	0.005	10	4.08	720	LSM14A, TRIM6, TAC1, UBE2D3, STAT6, CTSB, PDE4D, ADGRE2, IL15, MAPKAP1	26065, 117854, 6863, 7323, 6778, 1508, 5144, 30817, 3600, 79109
positive regulation of neuron death	GO:1901216	0.007	0.008	3	0.40	70	BCL2L11, GRIK2, TP53	10018, 2898, 7157
cellular protein metabolic process	GO:0044267	0.007	0.007	33	22.74	4011	DBNDD2, CALU, PRKAA1, LSM14A, TRIM6, CUL4B, SUV39H2, CPEB2, FBXO32, MORF4L2, PKIG, MITF, BCL2L11, WWTR1, UBE2D3, FMR1, ATXN3, SYNCRIP, RBPMS, PPP2R2B, MCTS1, GRIK2, CTSB, PDE4D, PLK4, NCOA7, MORF4L1, BRCA1, ATG4A, NRG1, TP53,	55861, 813, 5562, 26065, 117854, 8450, 79723, 132864, 114907, 9643, 11142, 4286, 10018, 25937, 7323, 2332, 4287, 10492, 11030, 5521, 28985, 2898, 1508, 5144, 10733, 135112, 10933, 672, 115201, 3084, 7157, 2070, 3600
regulation of transcription from RNA polymerase III promoter	GO:0006359	0.007	0.008	2	0.13	23	ZNF345, BRCA1	25850, 672
response to virus	GO:0009615	0.008	0.011	5	1.23	217	LSM14A, TRIM6, BCL2L11, FMR1, IL15	26065, 117854, 10018, 2332, 3600
response to lipid	GO:0033993	0.008	0.006	10	4.13	729	PRKAA1, TRIM6, GJB6, ESRRG, FBXO32, TAC1, STRN3, PDE4D, BRCA1, IL15	5562, 117854, 10804, 2104, 114907, 6863, 29966, 5144, 672, 3600

positive regulation of mitochondrion organization	GO:0010822	0.008	0.009	4	0.78	138	BCL2L11, UBE2D3, NRG1, TP53	10018, 7323, 3084, 7157
positive regulation of cytokine production	GO:0001819	0.008	0.007	6	1.74	307	LSM14A, TRIM6, STAT6, PDE4D, BRCA1, IL15	26065, 117854, 6778, 5144, 672, 3600
regulation of vesicle-mediated transport	GO:0060627	0.008	0.007	7	2.30	406	PRKAA1, FMR1, PREPL, SLC30A8, CACNA1G, ADGRE2, GOSR1	5562, 2332, 9581, 169026, 8913, 30817, 9527
cellular response to oxidative stress	GO:0034599	0.008	0.006	5	1.24	219	PRKAA1, CPEB2, STAT6, NCOA7, TP53	5562, 132864, 6778, 135112, 7157
regulation of synaptic vesicle exocytosis	GO:2000300	0.008	0.016	2	0.14	24	FMR1, PREPL	2332, 9581
positive regulation of release of cytochrome c from mitochondria	GO:0090200	0.008	0.005	2	0.14	24	BCL2L11, TP53	10018, 7157
establishment of protein localization to organelle	GO:0072594	0.008	0.006	8	2.92	515	PKIG, BCL2L11, WWTR1, UBE2D3, RPAIN, RBPMS, NRG1, TP53	11142, 10018, 25937, 7323, 84268, 11030, 3084, 7157
positive regulation of defense response	GO:0031349	0.009	0.004	6	1.78	314	LSM14A, TRIM6, TAC1, UBE2D3, CTSB, IL15	26065, 117854, 6863, 7323, 1508, 3600
regulation of cellular component biogenesis	GO:0044087	0.009	0.006	10	4.21	743	PRKAA1, CUL4B, TAC1, MITF, BCL2L11, FMR1, SEPT9, PLK4, BRCA1, TP53	5562, 8450, 6863, 4286, 10018, 2332, 10801, 10733, 672, 7157
positive regulation of histone modification	GO:0031058	0.009	0.007	3	0.43	76	FMR1, BRCA1, TP53	2332, 672, 7157
regulation of protein transport	GO:0051223	0.009	0.005	9	3.59	634	TRIM6, G6PC2, PKIG, WWTR1, UBE2D3, RBPMS, SLC30A8, NRG1, TP53	117854, 57818, 11142, 25937, 7323, 11030, 169026, 3084, 7157
centriole replication	GO:0007099	0.009	0.004	2	0.15	26	PLK4, BRCA1	10733, 672

positive regulation of multicellular organismal process	GO:0051240	0.009	0.007	14	7.08	1249	PRKAA1, LSM14A, TRIM6, TAC1, WWTR1, FMR1, CPNE1, FST, STAT6, PDE4D, BRCA1, ZEB1, NRG1, IL15	5562, 26065, 117854, 6863, 25937, 2332, 8904, 10468, 6778, 5144, 672, 6935, 3084, 3600
regulation of signaling	GO:0023051	0.010	0.010	24	15.12	2667	PRKAA1, LSM14A, TRIM6, G6PC2, TAC1, BCL2L11, WWTR1, FMR1, CPNE1, PREPL, FST, STRN3, RBPMS, GRIK2, PDE4D, SLC30A8, AHCYL1, BRCA1, ZEB1, NRG1, TP53, EYA4, IL15, MAPKAP1	5562, 26065, 117854, 57818, 6863, 10018, 25937, 2332, 8904, 9581, 10468, 29966, 11030, 2898, 5144, 169026, 10768, 672, 6935, 3084, 7157, 2070, 3600, 79109
cellular response to hormone stimulus	GO:0032870	0.010	0.008	8	2.99	528	PRKAA1, ESRRG, CPEB2, FBXO32, STRN3, CTSB, AHCYL1, BRCA1	5562, 2104, 132864, 114907, 29966, 1508, 10768, 672
regulation of heart rate	GO:0002027	0.010	0.009	3	0.45	79	TAC1, PDE4D, CACNA1G	6863, 5144, 8913
regulation of protein insertion into mitochondrial membrane involved in apoptotic signaling pathway	GO:1900739	0.010	0.011	2	0.15	27	BCL2L11, TP53	10018, 7157
positive regulation of protein insertion into mitochondrial membrane involved in apoptotic signaling pathway	GO:1900740	0.010	0.011	2	0.15	27	BCL2L11, TP53	10018, 7157
regulation of system process	GO:0044057	0.010	0.008	7	2.42	427	FBXO32, TAC1, FMR1, PDE4D, AHCYL1, NRG1, CACNA1G	114907, 6863, 2332, 5144, 10768, 3084, 8913
